# STWave: Fine‐Scale Spatial Structure Discovery in Microscopic‐Resolution Spatial Transcriptomics via Patchwise Wavelet Graphs

**DOI:** 10.1002/advs.77240

**Published:** 2026-08-19

**Authors:** Tao Jiang, Songming Zhang, Xiaofeng Chen, Xiongtao Xiao, Wenming Cao, Weikai Li, Tong Zhao, Bing Li, Xinyue Xu, Zhongshan Li

**Affiliations:** ^1^ School of Mathematics and Statistics Chongqing Jiaotong University Chongqing China; ^2^ Research Center on Neural Networks and Machine Learning Chongqing Jiaotong University Chongqing China; ^3^ School of Computer Science Nanjing University Nanjing China; ^4^ Faculty of Computer Engineering and Artificial Intelligence Shenzhen University of Advanced Technology Shenzhen China; ^5^ School of Life Sciences and Technology Xi'an Jiaotong University Xi'an Shaanxi China; ^6^ State Key Lab of Processors, Institute of Computing Technology Chinese Academy of Sciences Beijing China; ^7^ Department of Mathematics and Statistics Georgia State University Atlanta Georgia USA

**Keywords:** microscopic‐resolution spatial transcriptomics, spatial domain identification, wavelet transform

## Abstract

The advent of microscopic‐resolution spatial transcriptomics (μST) enables the mapping of tissue complexity at subcellular resolution. However, the massive increase in data volume introduces a critical “memory wall,” significantly limiting the applicability of existing computational methods on deployable hardware platforms. In addition, traditional single‐scale modeling approaches often struggle to disentangle weak biological signals from technical noise. To overcome these challenges, STWave is proposed as a scalable framework for spatial domain identification. Specifically, STWave first enables efficient computation during both training and inference by leveraging a decoupled patch‐based learning strategy, thereby mitigating hardware limitations without sacrificing global contextual information. Furthermore, STWave adopts a discrete wavelet transform to encode gene expression features across multiple scales, effectively capturing both global trends and fine‐grained details. Extensive experimental results show that STWave attains state‐of‐the‐art clustering performance while maintaining exceptional computational efficiency, enabling scalable analysis of ultra‐large spatial transcriptomics datasets under constrained computational resources. Across platforms including Visium HD, Xenium, and CosMx, the proposed STWave accurately resolves complex tissue structures, enabling the identification of distinct immune niches within the tumor microenvironment and revealing fine‐grained developmental substructures. These results support STWave as a robust and efficient tool for large‐scale μST analysis.

## Graphical abstract

STWave transforms massive microscopic‐resolution spatial transcriptomics into interpretable fine‐scale tissue maps through patch‐wise inference, wavelet‐based multi‐scale encoding, and dual‐domain reconstruction. It reduces noise while preserving weak spatial signals, enabling efficient analysis of 6 40 000 spots of 2.47 GB GPU memory and revealing tumor‐associated immune niches, amplified molecular markers, and developmental brain substructures that conventional methods often overlook.

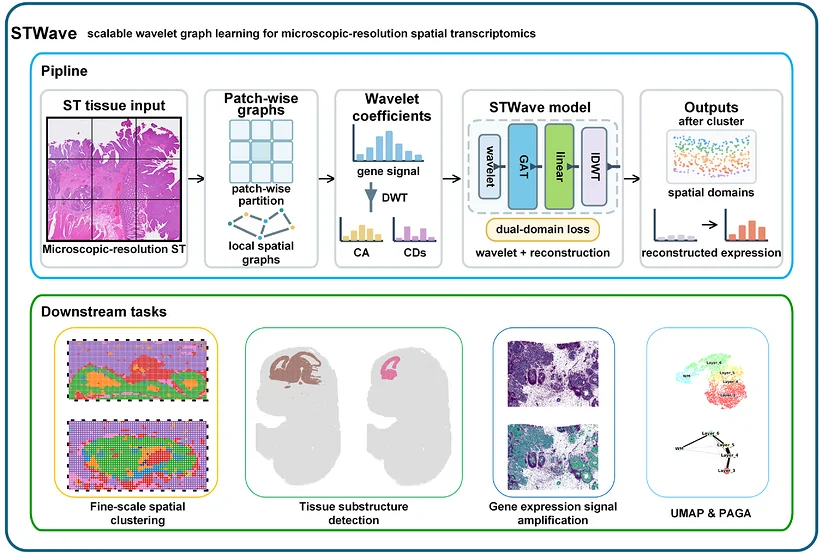

## 1 Introduction

Spatial biology is rapidly entering the era of microscopic‐resolution spatial transcriptomics (μST)[1], which enables whole‐transcriptome mapping at subcellular resolution with unprecedented spatial precision below 1μm[2]. Next‐generation ST platforms, including Visium HD [3], Xenium [4], GeoMx [5], and Stereo‐seq [6], have surpassed physical resolution limits through advanced molecular barcoding and imaging‐based capture strategies. For consistency, we use the term “spot” to denote the smallest addressable spatial sampling unit across all platforms. However, the gain in spatial precision incurs a substantial increase in sampling density [7]. To interrogate an equivalent tissue area, the required number of spatial spots increases exponentially. As a result, the massive scale and pronounced sparsity of μST data have created substantial computational challenges, widening the gap between biological resolution and current analytical feasibility [8].

Under this computational constraint, existing analysis pipelines encounter a practical “memory wall.” Most graph‐based clustering approaches, including Louvain [9] and stLearn [10], encode spatial context using simplified adjacency structures, which hinders both the scalability and reconstruction of fine‐scale cellular neighborhood organization. Similarly, probabilistic modeling approaches, including BayesSpace [11], BASS [12], and SpatialPCA [13], incur prohibitively high computational and memory costs as the number of spatial spots increases, hindering their application to large‐scale, whole‐tissue analyses. In parallel, state‐of‐the‐art deep learning–based methods, such as STAGATE [14], GraphST [15], conST [16], and SEDR [17], generally require the entire tissue‐level graph to be loaded into memory during training and inference. As ST datasets scale to millions of spots, existing approaches encounter severe scalability constraints. Even methods that introduce partitioned or mini‐batch training frequently require full‐graph loading during inference, failing to alleviate the underlying scalability bottleneck. However, the challenge of effective analysis extends beyond computational cost. A more fundamental limitation lies in adequately modeling complex biological heterogeneity across multiple scales [18]. Most existing methods preferentially model single‐scale variation, focusing on dominant high‐variance spatial patterns that reflect global tissue organization [19]. Consequently, fine‐grained tissue substructures and subtle molecular transition regions are often obscured by the high noise level inherent to μST data [7]. The computational limitations of existing methods necessitate the development of a scalable framework capable of handling million‐spot datasets while modeling biological variation across multiple scales and remaining computationally efficient.

To address these challenges, we design a scalable framework for ultra‐large μST data, which is referred to as STWave. By adopting a decoupled patch‐based learning strategy, STWave efficiently processes datasets containing hundreds of thousands of spots on standard, biologist‐accessible hardware. Unlike existing approaches, STWave adopts patch‐based computation during both training and inference, thereby avoiding full‐slice graph loading and maintaining low peak GPU memory usage as tissue size increases. In addition, the framework incorporates a discrete wavelet transform to address the intrinsic sparsity of ST data and perform multi‐resolution analysis. Specifically, it decomposes gene‐expression signals into approximation coefficients, which encode global expression structural patterns, and detail coefficients, which preserve localized high‐frequency signals. These multi‐scale features are integrated using a graph attention network, enabling the amplification of weak signals and attenuation of noise without constructing a massive global graph.

We evaluated STWave through comprehensive benchmarking across multiple platforms and conditions. In ultra‐large‐scale simulation studies, STWave exhibited excellent scalability, processing 6 40 000 spots in less than 20 min with a GPU memory footprint of 2.47 GB, while achieving leading clustering performance. In the 10x Visium DLPFC benchmark, STWave outperformed 17 competing methods, achieving an improvement of about 20% in clustering performance. In addition, STWave resolved fine‐grained biological structures on subcellular‐resolution platforms, including the identification of specific immune niches, such as $SPP1^{+}$ macrophages in Visium HD colorectal cancer samples, and the delineation of the Foxg1‐driven dorsal forebrain in Stereo‐seq mouse embryos. Furthermore, on Xenium and CosMx datasets, STWave improved molecular sensitivity and specificity while substantially reducing computational cost compared with recent partition‐based methods. Collectively, these results indicate that STWave provides a robust, accurate, and computationally efficient solution for analyzing large‐scale datasets characteristic of the μST era.

## 2 Results

### 2.1 Overview of STWave

STWave is a scalable deep learning framework that integrates wavelet representations to delineate spatial domains in μST data. STWave takes gene expression and spatial coordinates as input and partitions tissue sections into localized patches. Within each patch, spatial graphs are constructed and wavelet transforms are applied to extract multi‐scale features. The model is trained with a reconstruction loss (defined in Equation (14)) and subsequently performs batched inference over the full tissue to generate low‐dimensional embeddings that preserve both spatial context and gene expression. This patch‐based design enables scalable analysis by avoiding full‐slice loading. The overall workflow of STWave is illustrated in **Figure** 1.

**FIGURE 1:**
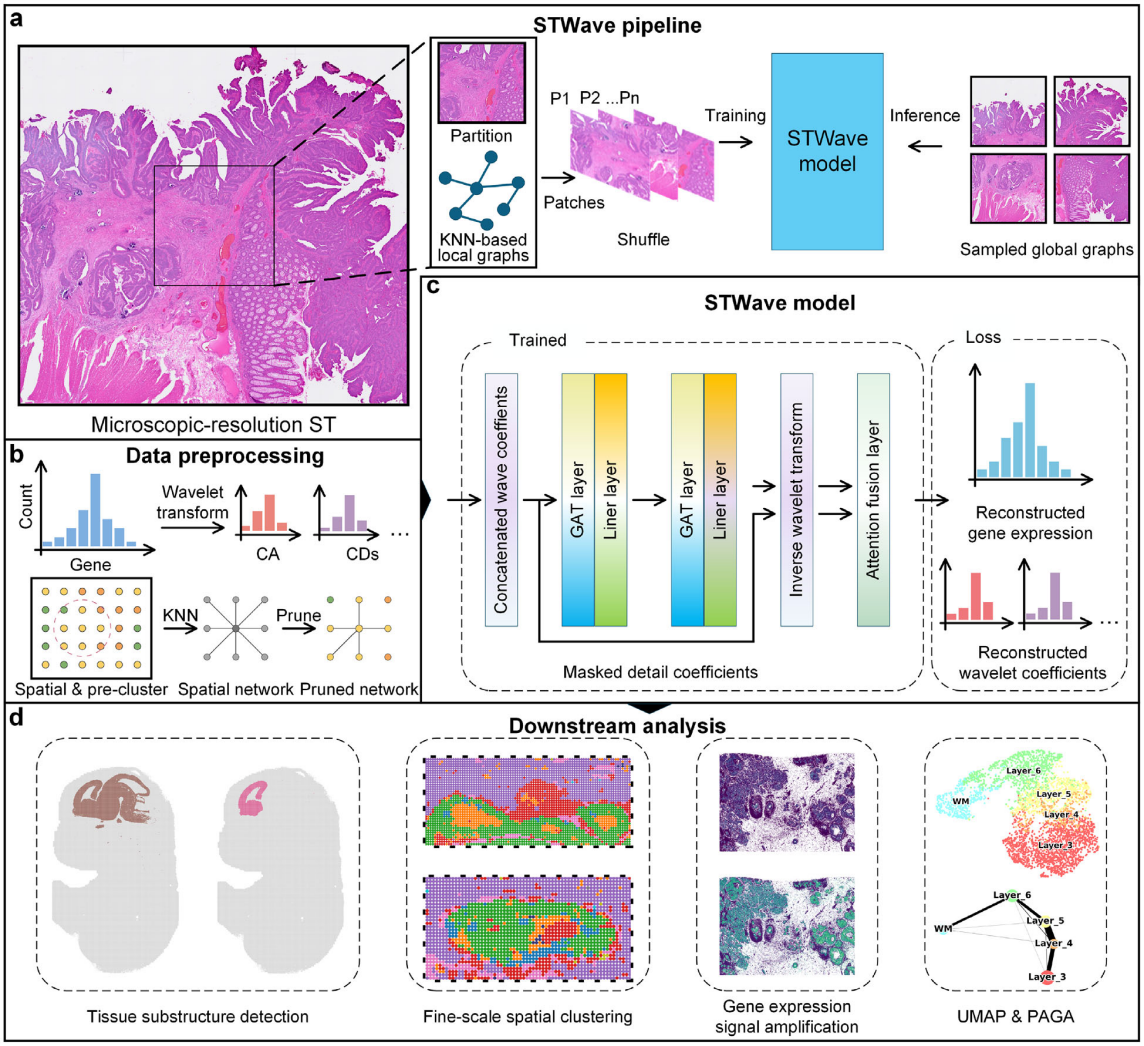
Schematic overview of the STWave framework. (a) STWave pipeline. The framework partitions a microscopic‐resolution ST slice into patches based on spatial coordinates and constructs local graphs. It shuffles these patches for iterative training and then performs patch‐based sampling inference to reconstruct the global result. (b) Data preprocessing. STWave decomposes gene expression signals into approximation coefficients (CA) and detail coefficients (CDs) using a 1D discrete wavelet transform. Simultaneously, it constructs spatial networks and pruned networks based on spatial locations and pre‐clustering results. (c) STWave model architecture. The unsupervised neural network utilizes Graph Attention Network (GAT) layers and linear layers for feature extraction. An attention fusion layer integrates the reconstructed features, while a dual‐term loss function jointly optimizes the model by minimizing reconstruction errors in both the wavelet and gene‐expression domains. (d) Downstream analysis tasks. The learned representations empower various applications, including substructure detection, fine‐scale spatial clustering, amplification of gene expression signals, and visualization via UMAP with PAGA trajectory inference.

To achieve scalability, we first partition each tissue slice into an m×m grid according to spatial coordinates, enabling patch‐wise training that effectively bounds peak memory usage. Within each patch, we construct a spatial neighborhood graph to model local connectivity and a pruned graph that removes noisy edges based on preliminary clustering assignments. In parallel, to capture biological heterogeneity across multiple spatial resolutions, we apply a one‐dimensional (1D) discrete wavelet transform as a multi‐scale encoding step. This operation decomposes the sparse expression profile of each spot into approximation coefficients that encode coarse tissue‐level patterns and detail coefficients that preserve fine‐grained, localized variations. These coefficients, combined with the graph structures, enable the model to learn robust representations. Finally, the network is optimized using a dual‐domain loss strategy: one component reconstructs the wavelet coefficients to preserve signal structure, while the other reconstructs the raw gene expression matrix to ensure biological fidelity.

In addition, STWave improves downstream task performance. With dual‐domain reconstruction, the model suppresses technical noise while amplifying weak yet biologically informative molecular signals. Here, signal amplification refers to an increase in the relative visibility of spatially coherent marker signals in the reconstructed expression map, rather than an absolute increase in raw transcript counts. Mechanistically, the wavelet‐domain reconstruction preserves multi‐scale signal structure, the expression‐domain reconstruction maintains gene‐level fidelity, and graph‐based aggregation reinforces signals that are spatially consistent across neighboring spots while reducing isolated technical noise. This results in refined gene expression profiles that preserve intricate biological patterns. Consequently, the learned embeddings support high‐precision spatial clustering, enabling the detection of fine‐grained tissue substructures and distinct boundaries. These spatially aware representations provide a foundation for downstream analyses, enabling visualization using methods such as UMAP and reliable trajectory inference with PAGA. Overall, STWave integrates patch‐based computation with multi‐scale modeling to efficiently analyze ultra‐large spatial datasets, eliminating the need for complex or specialized hardware configurations.

### 2.2 STWave Outperforms Seven Algorithms in Simulation

To evaluate the performance of STWave on ultra‐large ST data, we generated simulated datasets [20] based on the Human Lung Cell Atlas and benchmarked STWave against seven baselines: STAGATE, conST, GraphST, SEDR, SpaceFlow [21], PAST [22], and SpaSEG [23]. We conducted a two‐part evaluation. To assess computational efficiency, we recorded CPU memory usage, GPU memory usage, and wall‐clock time. To evaluate clustering performance, we measured the Adjusted Rand Index (ARI) [24], Normalized Mutual Information (NMI) [25], and Homogeneity Score (HS) [26]. All experiments were performed on the same hardware to ensure fair comparison, using an H20 GPU with 96 GB of memory and a CPU with 256 GB of memory.

We evaluated scalability under constrained computational resources to determine whether STWave can analyze ultra‐large ST data when other methods fail. Across all simulated datasets, STWave achieved the highest clustering fidelity, as indicated by the mean ARI (0.625±0.076) and NMI (0.701±0.061), while maintaining low GPU memory consumption (**Figure** 2a,d and Figure S1). Notably, STWave analyzed the 640000‐spot dataset within 20 min, using 2.47 GB of GPU memory and 30.34 GB of CPU memory. In contrast, although SpaSEG achieved the shortest runtime under the same setting, it required higher GPU (19.67 GB) and CPU (75.76 GB) memory and yielded lower ARI, NMI, and HS (Figure 2b,d, Figures S1 and S2 and Tables S2–S4). Across all scales, STWave maintained low and stable GPU memory usage, a critical advantage for analyzing ultra‐large datasets. By comparison, conST, GraphST, and SEDR failed when the number of spots exceeded 80000. Other methods performed adequately on smaller datasets but deteriorated beyond 240000 spots. For instance, PAST achieved an ARI of 0.209 at 640000 spots (Figure 2d and Figure S2). Collectively, these results demonstrate that STWave delivers accurate clustering with modest computational and memory requirements, enabling the analysis of ultra‐large ST datasets where competing methods either fail or demand substantial resources.

**FIGURE 2:**
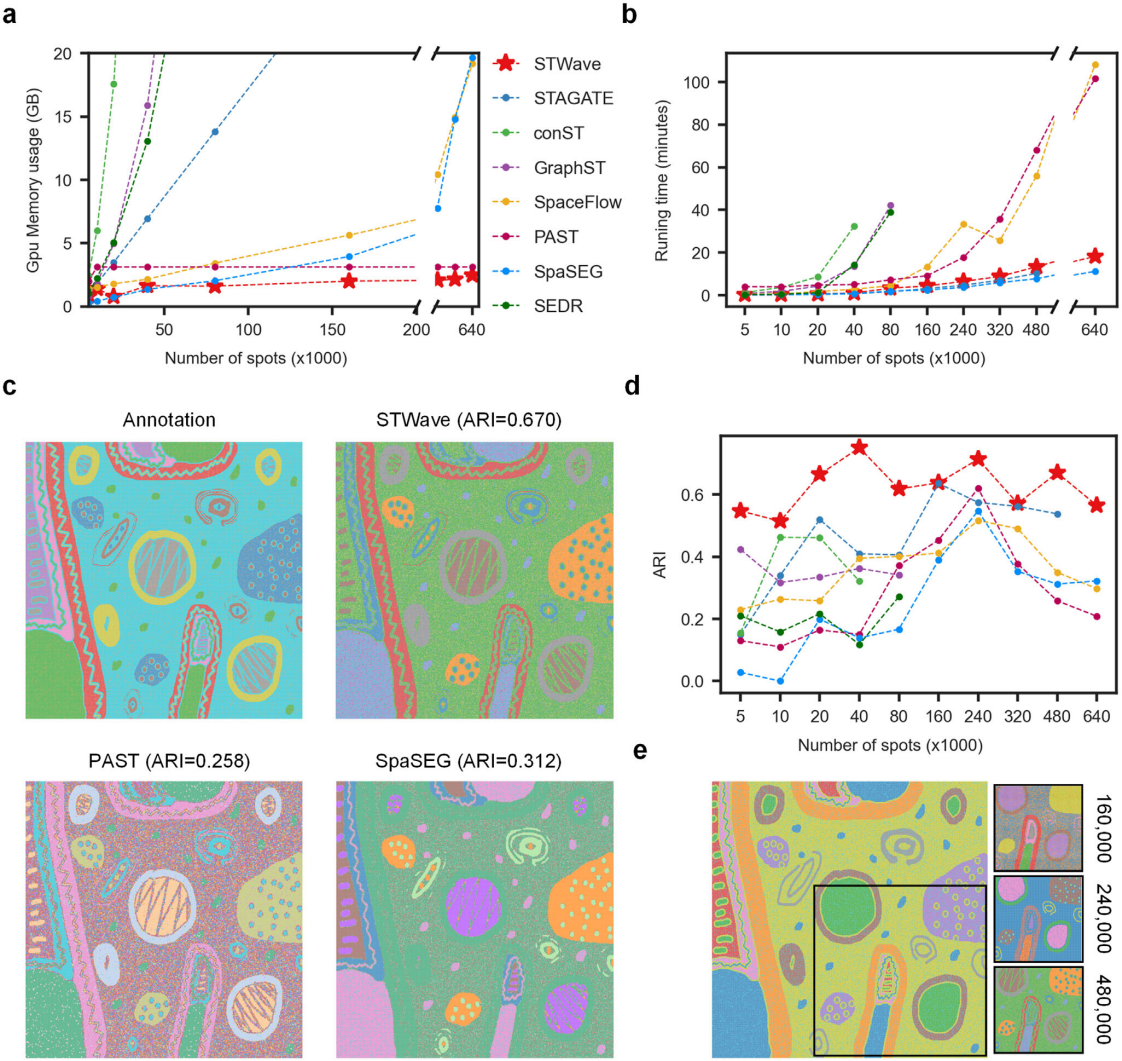
STWave shows robust performance on simulated datasets. **(a)** GPU usage of deep learning methods at different data sizes. **(b)** Runtime of different methods at different data sizes. **(c)** The top left panel shows an annotated spatial pattern in a setting with 480,000 spots. Spatial clustering results are shown for STWave (top right), PAST (bottom left), and SpaSEG (bottom right). Results for all methods are provided in the supplementary file. **(d)** ARI performance of each method at different data sizes. **(e)** The left panel shows an annotated spatial pattern in the 320000‐spot setting. The boxed region indicates the selected area. The right panels show STWave clustering within the selected region at 160000, 240000, and 480000 spots, arranged from top to bottom.

We next compared clustering visualizations to assess robustness across scales. STWave maintained stable performance and produced well‐defined spatial domains. At 480000 spots, SpaceFlow and SpaSEG achieved ARI values of 0.350 and 0.312, respectively, with noticeably over‐smoothed spatial domains. In contrast, STWave recovered finer structural details and achieved an ARI of 0.670, approximately 100% higher than SpaSEG (Tables S5 and S6). To examine scalability in a controlled setting, we used the 320,000‐spot dataset as a reference and generated datasets with 160000, 240000, and 480000 spots to mimic different resolutions. As dataset size increased, STWave progressively recovered finer spatial patterns (Figure 2c,e and Figures S3–S8). We further identified a practical operating point: when each patch contained approximately 10,000 spots, STWave achieved a favorable balance among GPU memory usage, CPU memory usage, runtime, and clustering accuracy (Table S7). Together, these results demonstrate that STWave is robust to scale and maintains consistent performance on ultra‐large ST datasets.

### 2.3 STWave Deciphers the Tumor Microenvironment in Colorectal Cancer

As one of the most prevalent cancers, colorectal cancer poses a significant global health challenge [27, 28]. Recent evidence increasingly highlights the pivotal role of immune cells in modulating the trajectory of the disease [29]. Tumor‐associated macrophages are a prominent and highly active component of the tumor microenvironment, where they play a critical role in tumor initiation and the progression of colorectal cancer [30]. ST captures gene expression in its native tissue context, enabling a more comprehensive characterization of the tumor microenvironment [31]. In this study, we analyzed a Visium HD colorectal cancer dataset from 10x Genomics aggregated at an 8μm×8μm spatial resolution. The dataset comprised 545913 spatial spots and 18,085 genes [32]. To evaluate spatial domain identification, we benchmarked STWave against STAGATE as a representative spatial method and principal component analysis (PCA) as a standard non‐spatial baseline.

Across spatial resolutions, STWave reconstructed tissue architecture with high fidelity and robustness, preserving the morphology and continuity of colonic glands while accurately resolving cord‐like and tubular glandular structures. In contrast, STAGATE and PCA produced fragmented and noisy partitions, exhibiting oversegmentation and undersegmentation within the same regions (**Figure** 3a,c). The improvements achieved by STWave were systematic and included sharper boundaries, sustained connectivity of fine glandular units, and reduced cross‐region leakage. Additionally, STWave revealed biologically informative cell populations that were not detected by competing approaches. Notably, STWave identified a distinct cluster of $SPP1^{+}$ macrophages encircling a calcified region (Figure 3b), consistent with prior observations [20]. The calcified regions in the H&E panels and the corresponding STWave‐identified spatial domains are delineated by blue outlines in Figure 3b, providing direct localization of the calcified foci and their surrounding macrophage‐enriched structures. The same regions were further compared with the matched PCA and STAGATE results in Figure S25, where the calcification‐associated domains were clearly preserved by STWave but were fragmented or spatially mismatched in the baseline results. $SPP1^{+}$ macrophages are associated with immune regulation and patient prognosis [33, 34, 35]. These cells assemble into spatially organized clusters, chains, or gland‐like rings surrounding tumor cells, patterns that are difficult to capture without robust spatial representations. Their absence in the results produced by STAGATE and PCA highlights STWave's superior ability to preserve low‐abundance yet biologically meaningful spatial signals.

**FIGURE 3:**
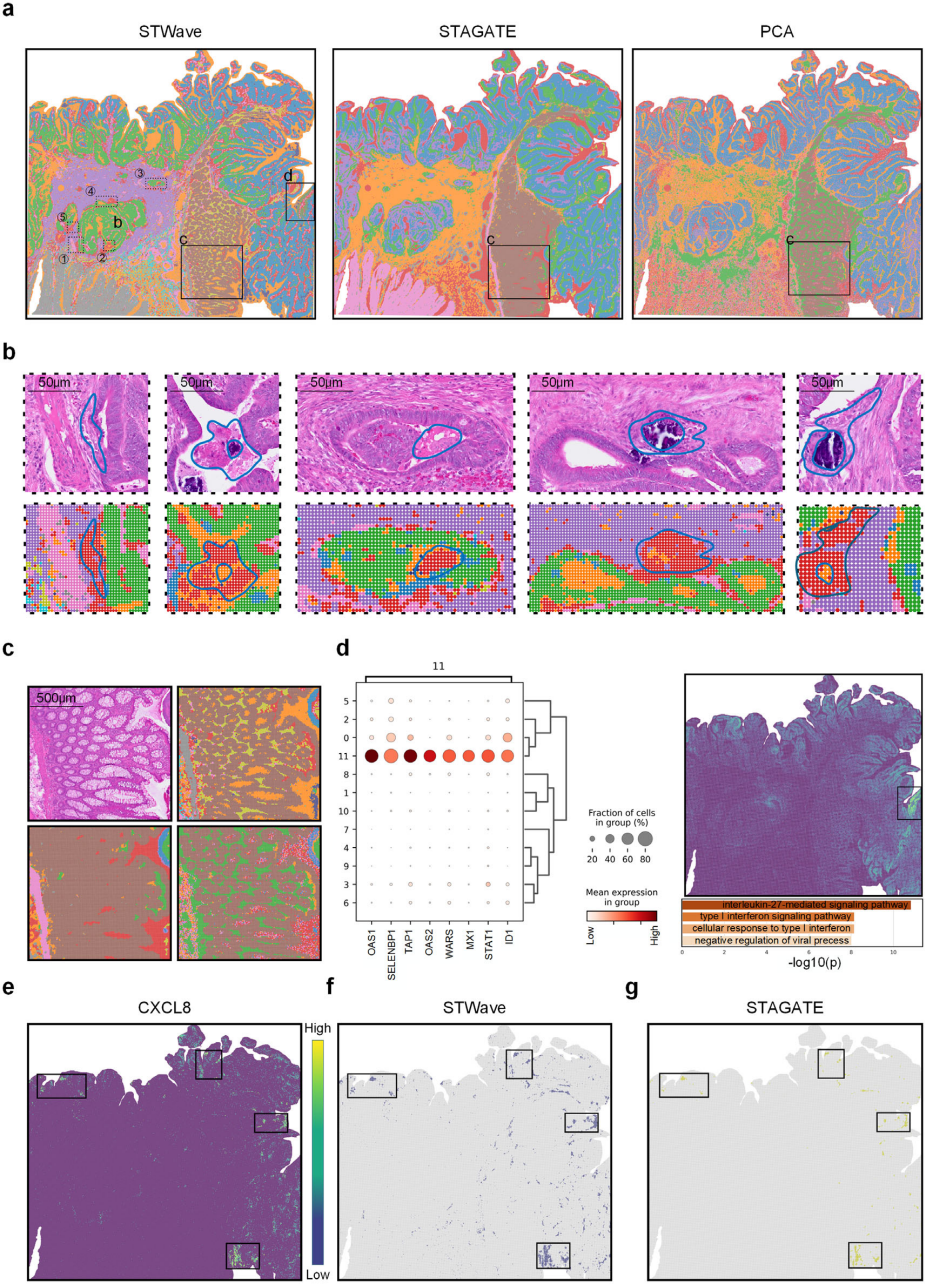
STWave enables the identification of fine structures in a human colorectal cancer dataset. **(a)** Spatial clustering visualizations of STWave (left), STAGATE (middle), and PCA (right). **(b)** H&E‐stained images of five selected regions and their corresponding STWave spatial clustering results, taken from region b boxed by a black dashed line in Figure 3a. Blue outlines in the upper H&E panels mark calcified regions, and blue outlines in the corresponding lower STWave clustering panels mark the spatial domains identified by STWave around these regions. **(c)** H&E‐stained images (top left) of region c boxed by a black solid line in Figure 3a, with the corresponding clustering result of STWave (top right), STAGATE (bottom left), and PCA (bottom right). **(d)** Dot plot (left) of genes with high expression in domain 11 identified by STWave, as shown for region d boxed with a black solid line in Figure 3a, showing spatial mean expression (top right) and pathway enrichment (bottom right) computed with the Python package gseapy. **(e)** Spatial expression plot of CXCL8. The blue box indicates the region of interest defined by the overlap between the CXCL8‐high region and the corresponding STWave‐identified spatial domain. **(f)** Separate spatial clustering result of STWave, consistent with Figure 3e. The box marks the same overlap region of interest. **(g)** Separate spatial clustering result of STAGATE, consistent with Figure 3e. The box marks the same region for comparison.

Moreover, STWave uncovered tumor subregions with distinct transcriptional programs and well‐defined spatial organization (Figure 3d). Highly expressed genes were organized into coherent spatial modules, and enrichment analyses indicated significant upregulation of immune‐associated pathways. These patterns are consistent with an enhanced anti‐tumor immune response driven by active immune cell infiltration within the tumor microenvironment [36]. In addition, STWave identified a spatially localized subregion with elevated CXCL8 expression (Figure 3e,f), indicative of a pro‐tumor inflammatory niche. The boxes in Figure 3e–g indicate the region of interest defined by the overlap between the CXCL8‐high region and the corresponding STWave‐identified spatial domain. Further analysis of this domain showed enrichment of inflammatory and cytokine‐related processes, including inflammatory response, regulation of interleukin‐1 beta production, regulation of inflammatory response, positive regulation of cytokine production, cellular response to cytokine stimulus, and regulation of tumor necrosis factor production. Additional pro‐tumor inflammatory markers, including S100A9, S100A8, IL1B, OSM, CSF3R, PTGS2, and PLAUR, also showed elevated expression in this region (Figure S26). This pattern is consistent with known mechanisms involving neutrophil recruitment, neutrophil extracellular trap formation, angiogenesis, and epithelial‐to‐mesenchymal transition, processes linked to tumor invasion, metastasis, and poor clinical outcomes [37, 38, 39]. By contrast, PCA and STAGATE failed to resolve these spatial domains (Figure 3a,g). Collectively, these results demonstrate that STWave robustly captures subtle yet clinically relevant spatial patterns that are overlooked by standard baseline methods, which tend to oversmooth fine‐grained structures or fragment domain boundaries, limiting their effectiveness for large‐scale spatial analyses.

### 2.4 STWave Detects Critical Molecular Signatures With High Sensitivity and Specificity

High‐resolution ST technologies, such as Xenium and NanoString CosMx SMI, provide unprecedented insight into the complex molecular landscape of cancer tissues, enabling substantial gains in both spatial resolution and transcriptomic coverage. However, these datasets are prone to signal saturation from highly expressed genes, which can obscure biologically meaningful signals from low‐abundance transcripts [4]. To address this limitation, we applied STWave to capture multi‐scale gene expression patterns in ultra‐large ST datasets and to amplify signals that exhibit intrinsic spatial structure.

We evaluated STWave on a Xenium human breast cancer dataset comprising 1 67 780 spots and 313 genes [4], and compared its performance with HERGAST [20], STAGATE, and PCA. Specifically, STWave outperformed all competing methods in terms of Adjusted Rand Index (ARI: 0.546) and Homogeneity Score (HS: 0.656), demonstrating a clear advantage in discriminating closely related cell types. Although HERGAST achieved a marginally higher Normalized Mutual Information (0.580 versus 0.547), STWave generated clusters with superior histological concordance and more faithful boundary delineation. By contrast, STAGATE exhibited pronounced oversmoothing and cluster merging in this dataset, leading to the loss of biologically meaningful local structures. In addition, STAGATE produced fragmented and irregular domain boundaries across tissue regions, thereby reducing overall spatial coherence (**Figure** 4a,b). To keep the main figure readable and highlight comparisons with PCA as a non‐spatial baseline and STAGATE as a spatial baseline, the complete HERGAST spatial visualization is provided in Figure S9, and the quantitative comparison is summarized in Figure S12. To verify that STWave enhances biologically meaningful signals in reconstructed expression, we examined the spatial patterns of key breast cancer markers in both the raw and reconstructed data, including CDH1, CEACAM6, EPCAM, and ERBB2 (Figure 4c; extended feature plots across multiple baselines are shown in Figures S10 and S11). The reconstructed expression patterns more clearly delineated a triple‐positive region, consistent with prior observations [4]. This observation was further supported by a quantitative assessment using the region‐specific relative marker signal gain (RSG), defined in Equations (S14)–(S18) as the ratio of the mean min–max‐normalized marker signal in the reconstructed map to that in the raw map within the corresponding H&E‐defined region. Across the selected marker‐region pairs in the Xenium human breast cancer dataset, the reconstructed maps showed consistently increased relative normalized marker signals, with RSG values ranging from 2.08× to 4.42× (Table S15). These results indicate that STWave increases region‐specific relative marker signal intensity while avoiding interpretation as an absolute expression fold change.

**FIGURE 4:**
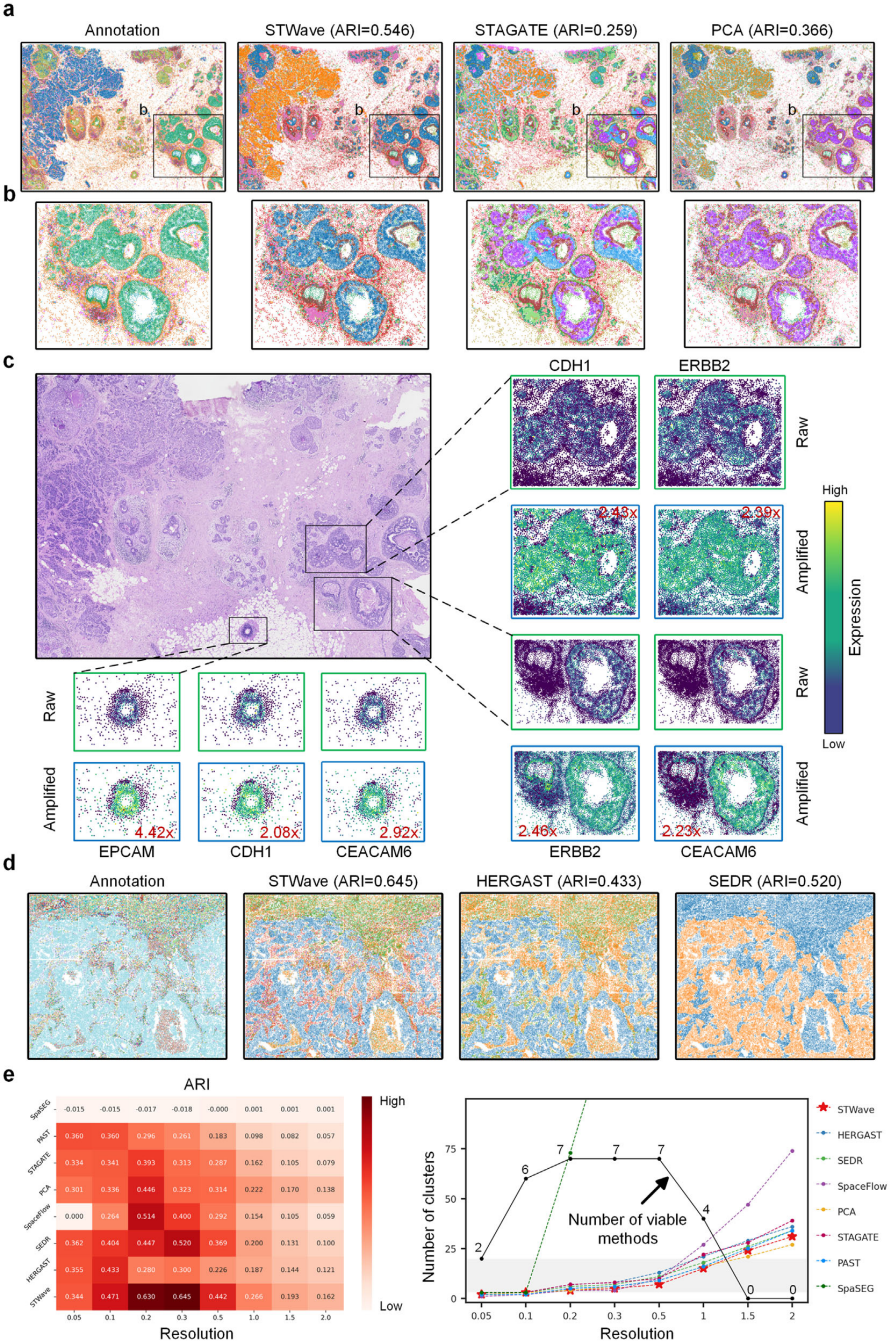
STWave performance on Xenium human breast cancer and CosMx SMI human lung cancer datasets. **(a)** From left to right are the annotation and the spatial clustering results from STWave, STAGATE, and PCA for Xenium human breast cancer, together with the corresponding ARI values. **(b)** Annotations and spatial clustering results for selected regions, taken from region b boxed with a black solid line in subpart (a). The complete Xenium spatial visualization for HERGAST is provided in Figure S9, with the quantitative comparison summarized in Figure S12. **(c)** H&E‐stained images of Xenium human breast cancer (top left). In the selected region we show raw and reconstructed spatial expression of ERBB2, EPCAM, CDH1 and CEACAM6. The annotated “×” values indicate the region‐specific relative marker signal gain (RSG), calculated as the ratio of the mean min–max‐normalized marker signal in the reconstructed map to that in the raw map within the corresponding H&E‐defined region. These values quantify relative normalized signal enhancement and do not represent absolute expression fold changes. **(d)** From left to right are the annotation and the spatial clustering maps from STWave, HERGAST, and SEDR for the CosMx SMI human lung cancer dataset, together with the corresponding ARI values. **(e)** Left: For the CosMx SMI human lung cancer dataset, we report ARI under Leiden clustering for different methods and parameters. Right: the corresponding number of clusters. The solid black line indicates the total number of methods that successfully identified between 3 and 20 clusters under the specified parameter settings.

As an additional evaluation in a disease tissue with fine‐grained spatial organization, STWave was applied to a Xenium human kidney cancer dataset. The analysis resolved multiple spatial domains and identified a localized domain enriched for vascular and endothelial signatures. This domain showed elevated expression of canonical endothelial markers, including PECAM1, VWF, CD34, ANGPT2, ADGRL4, and CLEC14A. Gene Ontology enrichment analysis of its marker genes further highlighted vascular‐related biological processes, including vasculogenesis, endothelial cell development, and glomerulus vasculature development. These results provide additional evidence that STWave can delineate fine‐scale disease‐associated structures beyond tissues with clearly defined domain architectures (Figure S24).

To further validate whether this enhanced sensitivity translates into robust and specific structural identification, we extended our analysis to a NanoString CosMx SMI single‐cell dataset (Lung 9‐1) comprising 87 589 spots and 960 genes [40]. We compared STWave against seven baselines (i.e., PCA, STAGATE, HERGAST, SEDR, SpaceFlow, SpaSEG, and PAST) across a wide range of Leiden [41] resolution parameters (0.05–2.0) to evaluate the stability and specificity of the identified spatial domains. SEDR was included in Figure 4d because it was one of the stronger baseline methods in this CosMx comparison, whereas the complete set of baseline visualizations is provided in Figure S13. STWave achieved the highest ARI of 0.645 and produced partitions that consistently aligned with histological annotations. Notably, STWave maintained biologically plausible cluster numbers across a broad range of intermediate resolution settings. By contrast, baseline methods frequently exhibited under‐segmentation at low resolution settings and fragmented over‐segmentation at higher resolutions, reflecting limited specificity in resolving true biological boundaries (Figure 4e; complete resolution‐wise evaluations and clustering results for all baselines are provided in Figures S13 and S14). Finally, we confirmed that these performance gains are achieved without incurring substantial computational overhead. Compared with HERGAST (5×5 patch), STWave markedly reduced resource consumption, decreasing GPU memory usage from 42.83 to 2.81 GB, CPU memory usage from 292.81 to 6.45 GB, and runtime from 13.56 to 4.85 min (Figure S14). Collectively, these results demonstrate that STWave supports accurate, specific, and computationally efficient analysis of ultra‐large ST datasets.

### 2.5 STWave Reveals Fine‐Grained Brain Substructures and Reconstructs Tissue Architecture in Mouse Embryos

To assess the ability of STWave to resolve complex developmental architectures, we analyzed Stereo‐seq mouse embryo datasets spanning embryonic stages E13.5 to E16.5[6]. We conducted detailed benchmarking on the E16.5 embryo dataset (**Figure** 5a), comprising 155741 spots and 28579 genes, to evaluate spatial domain resolution. In systematic comparisons with SpaceFlow, STAGATE, SpaSEG, and PAST, STWave achieved the highest histological concordance, with an adjusted rand index (ARI) of 0.547, exceeding the second‐best method, SpaceFlow (0.485), by more than 10%.

**FIGURE 5:**
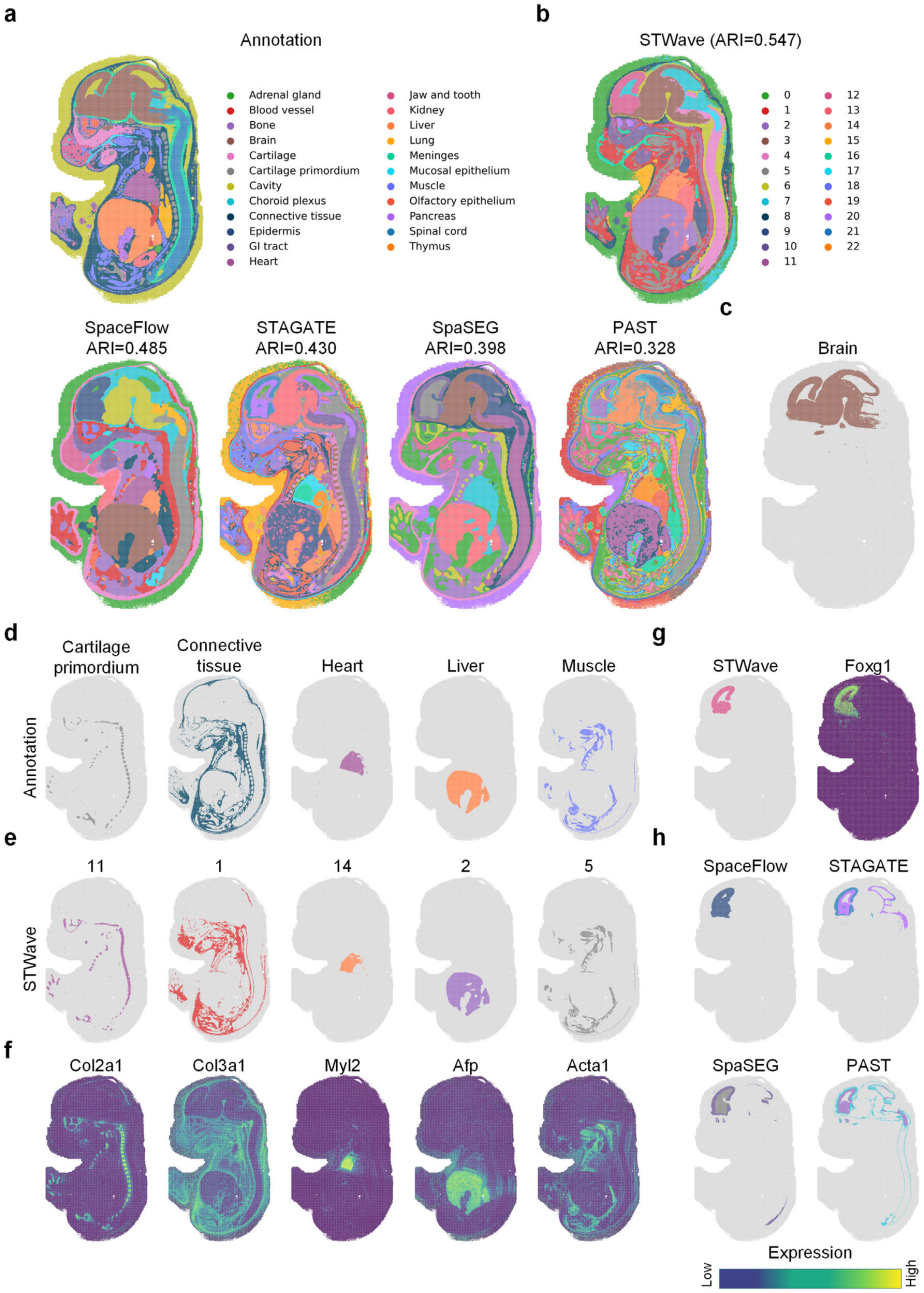
STWave accurately identifies spatial domains in mouse embryo tissues. **(a)** Visualization of reference spatial annotations on the mouse embryo E16.5 ST dataset. **(b)** Spatial clustering visualizations of STWave and competing methods; all results are in Figure S15. **(c)** Standalone visualization of the region annotated as Brain. **(d)** Standalone visualizations of several reference annotations. **(e)** Standalone visualizations of spatial domains identified by STWave that correspond to the annotations in Figure 5d. More examples can be found in Figure S16. (**f)** Spatial expression of representative genes corresponding to Figure 5d,e. **(g)** Standalone visualization of the dorsal forebrain identified by STWave (left) and the spatial expression of Foxg1 (right). **(h)** Spatial domains identified by competing methods that include the dorsal forebrain.

Although all methods successfully delineated major organ structures, STWave exhibited superior boundary delineation and structural continuity. It accurately resolved key tissue regions—including the cartilage primordium, connective tissue, heart, liver, and muscle—without the fragmentation or cross‐region merging observed in baseline methods such as STAGATE and SpaSEG (Figure 5b,d and Figures S15 and S16). To validate the biological relevance of the identified domains, we examined their correspondence with the spatial expression of canonical marker genes, including Col2a1 (cartilage), Myl2 (heart), and Afp (liver). The strong concordance between STWave‐defined clusters and these molecular signatures confirms the robustness of STWave in preserving tissue identity (Figure 5d–f and Figures S16–S18).

Notably, STWave demonstrated a distinct advantage in resolving fine‐grained brain substructures. It specifically identified a dorsal forebrain subregion that spatially coincides with a localized domain of high Foxg1 expression (Figure 5c,g,h). Foxg1 functions as a key regulator of telencephalon development and cortical patterning, with a central role in forebrain formation [42, 43]. In contrast to other methods, which failed to distinguish this region, STWave accurately captured this biologically distinct domain. These results are consistent with the established functional architecture of the embryonic brain and indicate that STWave enables not only the identification of broad tissue types but also the fine‐grained resolution required for mechanistic studies of organogenesis.

### 2.6 Superior Accuracy of STWave in Decoding the Spatial Architecture of the Human Dorsolateral Prefrontal Cortex

The quality of learned representations is critical for accurately decoding complex spatial architectures. To rigorously evaluate this aspect, we assessed STWave on the 10x Visium DLPFC benchmark, which comprises 12 tissue sections from three donors [44]. We performed a comparison against 17 baselines spanning diverse methodological classes, including deep learning‐based approaches (e.g., STAGATE, GraphST, and DeepGFT [45]) and probabilistic modeling frameworks (e.g., BayesSpace). Quantitatively, STWave achieved state‐of‐the‐art performance, attaining the highest mean Adjusted Rand Index (ARI: 0.642) and Normalized Mutual Information (NMI: 0.654) across all tissue sections, outperforming all competing methods (**Figure** 6b). The complete slice‐specific metric comparison is provided in Tables S8 and S9, and the corresponding summary plots and clustering visualizations are shown in Figures S19 and S20. Notably, STWave yielded an improvement of approximately 20% in mean ARI over the next best‐performing method, DeepGFT (0.519).

**FIGURE 6:**
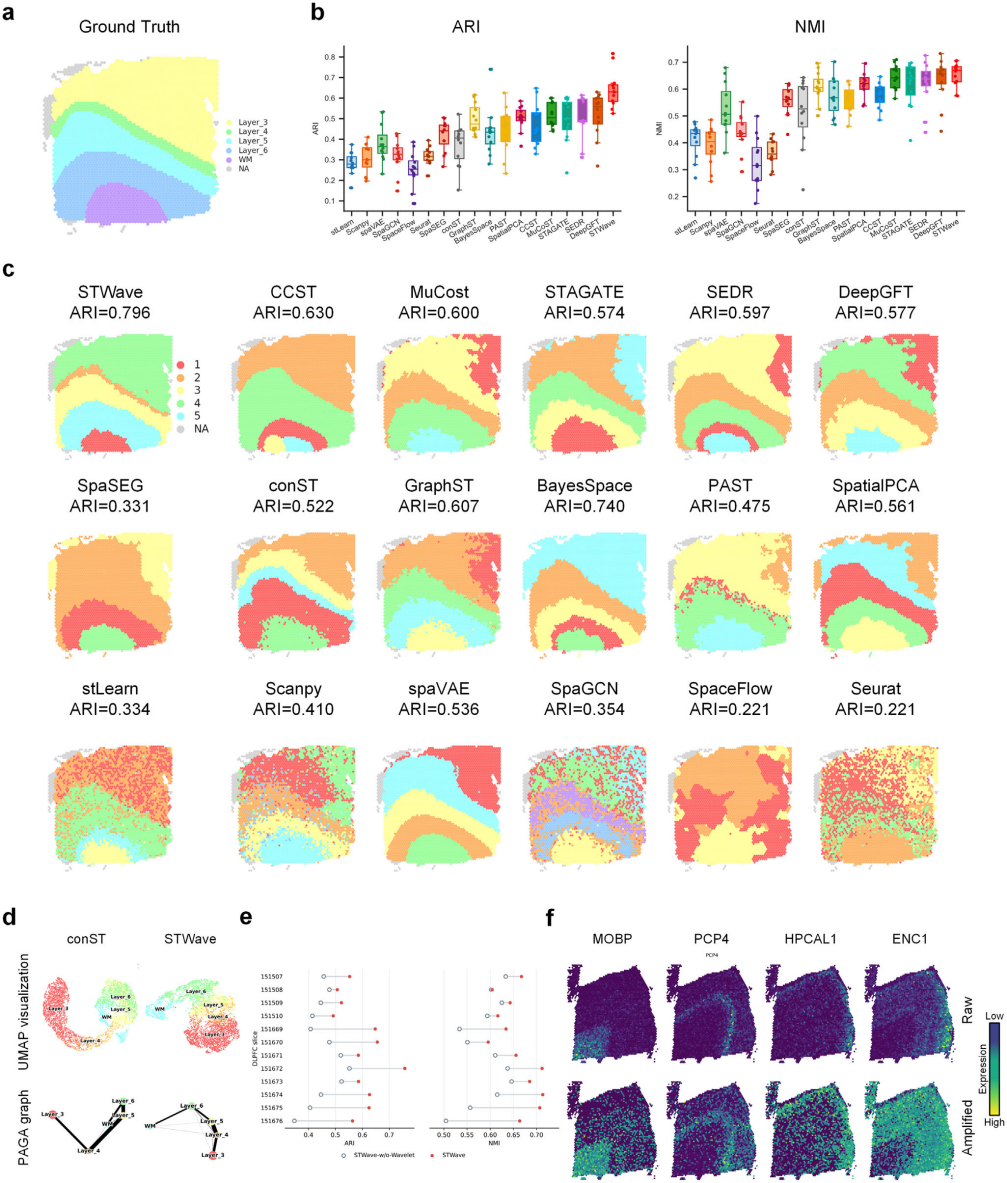
STWave performance on 10x Visium DLPFC ST datasets. **(a)** Visualization of reference annotations on slice 151672. **(b)** Box plots of ARI and NMI across 12 DLPFC slices. **(c)** Clustering visualizations for STWave and competing methods. **(d)** UMAP visualization and PAGA graph for STWave and conST on slice 151672. **(e)** Dumbbell plots comparing the ARI and NMI of STWave and STWave without the wavelet transform across 12 DLPFC slices. **(f)** Spatial expression of MOBP, PCP4, HPCAL1, and ENC1, illustrating a comparison between raw and STWave‐amplified signals.

To provide a stricter clustering benchmark beyond ARI and NMI, the DLPFC comparison was further expanded to six additional metrics: Adjusted mutual information (AMI), homogeneity score (HS), Completeness, V‐measure, Fowlkes–Mallows Index (FMI), and Purity. Among all evaluated methods, STWave ranked first across all eight metrics, including ARI (0.642±0.089), NMI (0.654±0.045), FMI (0.727±0.085), and Purity (0.810±0.054), with the complete metric panel reported in Figure S29. These results indicate that the advantage of STWave is not restricted to a single clustering metric.

Building on these benchmark results, we used the same controlled ablation to determine whether the contribution of the wavelet‐transform module extended beyond downstream clustering. In the ablation variant, only the wavelet‐transform module was removed, while the remaining neural‐network architecture and training settings were kept unchanged. On the DLPFC benchmark, STWave outperformed the ablation variant under Mclust clustering, increasing ARI from 0.456±0.080 to 0.593±0.074 and NMI from 0.592±0.055 to 0.658±0.041 across 12 slices and three random trials (Figure 6e, Figure S28 and Table S17). In the clustering‐agnostic evaluation, STWave showed improved preservation of global distance structure and local neighborhoods in the learned embeddings across the 12 DLPFC slices (Figure S30c). In the Xenium human breast cancer regions, STWave generally showed closer agreement with the observed marker expression and lower reconstruction errors across seven region‐of‐interest–marker pairs (Figure S30a,b). Collectively, these results indicate that the contribution of the wavelet‐transform module is evident in both the learned embeddings and reconstructed expression and is not limited to improvements in clustering metrics.

To further assess whether performance remains stable when more genes are used, STWave was independently evaluated on the DLPFC dataset with 1000, 2000, 3000, 4000, 5000, and 6000 highly variable genes. The ARI and NMI distributions remained broadly comparable across these settings, indicating that STWave remained stable when the input gene set was expanded beyond the 1K‐level targeted panels used by Xenium and CosMx SMI (Figure S27). At the individual‐slice level, we next analyzed slice #151672, which exhibits well‐defined cortical layers and white matter (Figure 6a). STWave achieved an adjusted rand index (ARI) of 0.796 and a normalized mutual information (NMI) of 0.678, accurately delineating all laminar boundaries in close agreement with manual annotations (Figure 6c and Tables S8 and S9). By contrast, baseline methods exhibited distinct topological failure modes. Although BayesSpace achieved a relatively high normalized mutual information (NMI: 0.702), it failed to resolve the biological boundary between Layers 4 and 5 and erroneously subdivided Layer 6 into two domains. Similarly, MuCoST, STAGATE, SEDR, and DeepGFT misclassified Layer 3 by fragmenting it into discontinuous regions. Further validation using UMAP and PAGA analyses confirmed that STWave can capture continuous biological trajectories underlying tissue organization. The learned embeddings distinctly separated layer‐specific spots while preserving smooth transitions that recapitulate the physical continuity of the tissue. In contrast, methods such as conST failed to recover this connectivity or to accurately resolve laminar boundaries. This improved performance is likely attributable to the integration of a discrete wavelet transform with a graph attention autoencoder, which enhances layer‐specific molecular signals while attenuating noise (Figure 6d,f and Figures S21 and S22).

## 3 Conclusion

STWave represents a scalable computational paradigm designed to bridge the gap between the massive data volumes of the μST era and constrained hardware resources. As ST technologies advance toward subcellular resolution, the exponential increase in spot numbers poses a substantial memory barrier for existing analytical tools. Our framework addresses this challenge by introducing a unified pipeline tailored for ultra‐large ST datasets. It enables routine analysis of hundreds of thousands of spots on standard computing platforms without compromising biological resolution. By formulating spatial domain identification as a joint problem of computational efficiency and signal enhancement, STWave offers a robust solution for decoding tissue heterogeneity at microscopic scales.

The effectiveness of STWave arises from two core mechanisms that jointly balance scalability and accuracy. First, the partition‐and‐sample strategy addresses the memory bottleneck by enabling patch‐based computation during both training and inference. Second, the integration of a discrete wavelet transform mitigates extreme sparsity and noise characteristic of subcellular‐resolution data. Specifically, approximation coefficients capture global tissue patterns, whereas detail coefficients preserve sharp spatial boundaries. The dual‐domain reconstruction loss further encourages the model to learn representations that are consistent across the wavelet and gene‐expression domains, effectively suppressing technical noise while retaining weak but biologically meaningful signals.

Extensive validation across diverse ST platforms underscores STWave's ability to uncover biologically meaningful patterns that are overlooked by existing approaches. Beyond improvements in standard quantitative metrics, STWave consistently resolved subtle yet clinically relevant structures. For example, in colorectal cancer, it delineated the spatial niche of $SPP1^{+}$ tumor‐associated macrophages and pro‐tumor inflammatory regions enriched for CXCL8. In mouse embryonic development, STWave uniquely identified a dorsal forebrain subregion defined by Foxg1, in close agreement with established histological knowledge. Moreover, STWave outperformed 17 baselines on the Visium DLPFC benchmark, demonstrating superior resolution of fine‐grained cortical layers. These results indicate that the combination of multi‐scale modeling and efficient partitioning enables robust generalization across diverse biological contexts and ST technologies.

Despite these advantages, several limitations suggest directions for future improvement. Fixed‐size patching may disrupt continuous spatial structures at boundaries, which could be alleviated by adaptive or overlapping strategies. To quantify this effect, boundary and interior regions were compared on the Xenium human breast cancer dataset under different boundary ranges. The boundary‐region ARI and NMI remained comparable to the corresponding interior‐region values, suggesting that the patch‐based strategy did not introduce substantial performance degradation at patch boundaries in this dataset (Table S14). In addition, representing gene expression as a 1D sequence does not fully capture gene‐gene regulatory relationships; graph‐based embeddings or order‐invariant transforms may provide more expressive alternatives. Finally, extending STWave from two‐dimensional (2D) sections to three‐dimensional (3D) reconstruction or multi‐modal integration would further enhance its applicability.

## 4 Experimental Section

### 4.1 Data Description

We evaluated STWave on a collection of ST datasets spanning multiple technologies and spatial resolutions. The study encompasses five platforms: 10x Genomics Visium HD, 10x Genomics Xenium, NanoString CosMx SMI, BGI Stereo‐seq, and 10x Genomics Visium. Among these, Visium HD achieves near‐cellular resolution with 2×2μm features across a 6.5mm×6.5mm tissue area, with measurements that can be aggregated into coarser spatial spots ranging from 8 to 16μm. Xenium is an imaging‐based in situ platform that provides single‐cell to subcellular resolution using ready‐to‐use panels of 5000 genes, with options for small add‐ons or fully custom designs. NanoString CosMx SMI similarly achieves single‐cell to subcellular resolution and supports RNA panels targeting about 6000 genes in both FFPE and fresh‐frozen tissues. Stereo‐seq employs DNA nanoball–patterned arrays on flexible chip formats, enabling spatial binning from subcellular to multicellular scales. In contrast, the Visium platform profiles transcriptomes using 55 μm circular spots across a 6.5mm×6.5mm capture area, providing multicellular resolution with one to ten cells per spot. Collectively, these platforms span spatial resolutions from multicellular to subcellular scales, motivating the development of methods that preserve fine‐grained biological detail while maintaining computational efficiency.

To evaluate the accuracy of spatial domain identification, we benchmarked STWave on a human dorsolateral prefrontal cortex (DLPFC) dataset comprising 12 tissue slices from three donors, with each capture area containing about 4000 spots and 33 538 genes. To assess performance at single‐cell and subcellular resolutions, we analyzed a diverse array of high‐resolution ST datasets: a Visium HD human colorectal cancer slice (P2) containing 5 45 913 spots and 18 085 genes; two Xenium datasets, including a human breast cancer dataset featuring 1 67 780 spots across 313 genes and a human kidney cancer dataset containing 56 510 spots across 377 genes; and a CosMx SMI lung cancer dataset consisting of 87 589 spots and 960 genes. The longitudinal Stereo‐seq mouse embryo series (stages E13.5–E16.5) was further utilized, with the E16.5 sample totaling 1 55 741 spots and 28 579 genes. To test high‐resolution scalability under resource‐constrained environments, we generated a series of simulated datasets from the Human Lung Cell Atlas, spanning $5\times 10^{3}$ to $6.4\times 10^{5}$ spots. This comprehensive collection covers a continuous spectrum from small‐scale to ultra‐large‐scale data. Collectively, these benchmarks evaluate whether STWave preserves spatial fidelity and maintains manageable resource consumption as data volume increases, while highlighting where baseline methods succumb to oversmoothing of fine structures, boundary fragmentation, or computational failure.

### 4.2 Data Preprocessing

Gene expression data were processed using Scanpy. For datasets containing at least 3000 genes, highly variable genes were identified from the raw counts using the Seurat v3 method, and the top 3000 highly variable genes were retained. The resulting expression matrix was then normalized on a per‐spot basis and log‐transformed. For datasets with fewer than 3000 genes, all genes were retained before normalization and log transformation, as in the Xenium human breast cancer and CosMx SMI human lung cancer datasets.

### 4.3 Spatial Neighbor Graph Construction

We construct a spatial graph based on the Euclidean distances between spot coordinates. Spatial relationships are encoded as an undirected k‐nearest‐neighbor graph, denoted $G_{\mathrm{spatial}}=\left(V_{\mathrm{spatial}},E_{\mathrm{spatial}}\right)$, where $V_{\mathrm{spatial}}$ denotes the set of spots and $E_{\mathrm{spatial}}$ denotes the set of undirected edges connecting neighboring spots. The adjacency matrix is denoted $A_{\mathrm{spatial}}\in {\left\{0,1\right\}}^{n\times n}$, where n is the number of spots. For vertices i and j, we set $a_{\mathrm{spatial}}^{ij}=1$ if j belongs to the set of k nearest neighbors of i, and $a_{\mathrm{spatial}}^{ij}=0$ otherwise. Each spot is connected to its k nearest neighbors, and the resulting graph is treated as undirected. In our experiments, we set k=8 for simulated data, Visium HD, Xenium, and Stereo‐seq datasets, and k=6 for standard 10x Visium datasets.

Based on the constructed spatial graph, STWave prunes edges that are unlikely to convey meaningful local information. We first embed the gene expression matrix using PCA and obtain an initial partition of spots via the Louvain algorithm. Edge pruning is then performed in two stages. In the first stage, edges connecting spots assigned to different clusters are removed to prevent premature information propagation across heterogeneous regions. In the second stage, spurious long‐range connections are eliminated using distance‐based outlier detection with an Isolation Forest [46]. Specifically, edges with unusually large Euclidean lengths (e.g., exceeding the global mean plus one standard deviation) are flagged and removed. The resulting pruned graph is denoted $G_{\mathrm{pruned}}=\left(V_{\mathrm{pruned}},E_{\mathrm{pruned}}\right)$.

### 4.4 Wavelet Representation of Gene Expression

ST data are high‐dimensional and sparse, characterized by pronounced zero inflation, technical noise, multi‐scale texture, and fragile tissue boundaries. These properties often lead to over‐smoothing, attenuation of weak biological signals, and unstable alignment across tissue sections. To disentangle background variation from edge information across multiple scales, we represent the expression profile of each spot as a 1D discrete signal ordered by gene index and apply a 1D discrete wavelet transform [47]. This 1D representation is used as a computationally efficient multi‐scale feature transform rather than as an explicit model of gene regulatory interactions. To assess whether the imposed gene order affects downstream performance, randomized gene‐order experiments were conducted on the DLPFC dataset, where ARI and NMI remained stable across five random orders (Figure S23).

Let $X\in R^{n\times m}$ denote the gene expression matrix, where n is the number of spots and m is the number of genes. For each spot‐specific expression vector $x_{i}\in R^{m}$, with i=1,…,n, we initialize $a_{0}=x_{i}$ and apply the discrete wavelet transform as follows:

(1) $$\begin{array}{cc} a_{j+1}\left[r\right] & =\sum_{k=0}^{N-1}h\left[k-2r\right]\cdot a_{j}\left[k\right], \end{array}$$

(2) $$\begin{array}{cc} d_{j+1}\left[r\right] & =\sum_{k=0}^{N-1}g\left[k-2r\right]\cdot a_{j}\left[k\right], \end{array}$$

where $a_{l}$ denotes the approximation coefficients at the decomposition level l, capturing low‐frequency trends obtained via low‐pass filtering and downsampling. $d_{l}$ denotes the corresponding detail coefficients, representing high‐frequency components extracted through high‐pass filtering and downsampling. The filters h and g correspond to low‐pass and high‐pass kernels, respectively. The index k denotes the convolution offset, N is the length of the input signal at the current level, and r indexes the resulting output coefficients. After j levels of wavelet decomposition, each expression vector is represented by a multiscale coefficient set $\left(a_{j},d_{j},\text{…},d_{2},d_{1}\right)$.

We construct a cross‐scale representation by concatenating wavelet coefficients across decomposition levels and frequency bands. Specifically,

(3) $$\begin{array}{cc} c_{i} & =\mathrm{concat}(a_{j},d_{j},\text{…},d_{1}), \end{array}$$

(4) $$\begin{array}{cc} C & =\mathrm{concat}\;{\left(c_{1}^{T},c_{2}^{T},\text{…},c_{n}^{T}\right)}^{T}, \end{array}$$

where $c_{i}$ denotes the wavelet coefficient vector for spot i, and $C\in R^{n\times d_{\mathrm{wc}}}$ aggregates all spots along the sample dimension, with $d_{\mathrm{wc}}$ representing the total number of coefficients across all scales.

To further enhance representation learning in the setting of limited computational resources, we construct a masked coefficient matrix $C_{\mathrm{mask}}$ as a complementary view to the complete wavelet features. Let $c_{i}=\left[a_{j},d_{j},\text{…},d_{1}\right]\in R^{d_{\mathrm{wc}}}$ denote the wavelet coefficient vector for spot i. A binary mask $m\in {\left\{0,1\right\}}^{d_{\mathrm{wc}}}$ is applied to retain approximation coefficients ($a_{j}$) while suppressing high‐frequency detail components, yielding

(5) $$c_{i}^{\mathrm{mask}}=c_{i}⊙m,$$

where ⊙ denotes element‐wise multiplication. Stacking these vectors forms $C_{\mathrm{mask}}\in R^{n\times d_{\mathrm{wc}}}$, which emphasizes low‐frequency global expression patterns and reduces sensitivity to technical noise.

### 4.5 Subgraph Partitioning and Sampling

To ensure the scalability of STWave while minimizing GPU and CPU overhead, we implement a partition‐and‐sample subgraph strategy. The global ST dataset is first decomposed into spatially contiguous patches based on coordinates, with the original spot indices preserved to maintain data integrity. The granularity of this partitioning is governed by a tunable hyperparameter, m, which represents the number of patches along each dimension. To optimize the trade‐off between computational efficiency and neighborhood preservation, we recommend an m×m grid configuration where $m=\mathrm{round}\left(\sqrt{n/10,000}\right)$ (Table S7). This heuristic ensures that each patch contains approximately 10 000 spots on average, preventing the loss of local structural information associated with overly fine partitioning while avoiding the prohibitive memory demands of excessively large patches. Leveraging the stored indices, we utilize the PyTorch Geometric (PyG) framework to sample subgraphs from both the initial spatial graph $G_{\text{spatial}}$ and the pruned graph $G_{\text{pruned}}$. This sampling mechanism is designed to preserve internal patch topology and maintain critical boundary connectivity across the spatial domain.

### 4.6 Graph Attention Autoencoder

Inspired by STAGATE [14], we employ a four‐layer autoencoder composed of two graph attention blocks and two linear layers to learn graph‐informed embeddings for ST analysis. The encoder consists of two layers. The first layer is a multi‐head graph attention layer that aggregates features from local neighborhoods, while the second layer applies a linear projection to generate low‐dimensional spot embeddings. We denote $H^{0}=C\in R^{n\times d_{\mathrm{wc}}}$ as the input feature matrix, where n denotes the number of spots and $d_{\mathrm{wc}}$ is the dimension of the wavelet coefficient representation. The adjacency matrix $A\in R^{n\times n}$ defines candidate spatial neighbors, and $S_{i}$ denotes the neighborhood of spot i, including the spot itself. The first graph attention layer is defined as

(6) $$\begin{array}{cc} h_{i}^{1} & =\sigma_{1}\;\left(\frac{1}{Q}\sum_{q=1}^{Q}\;\sum_{j\in S_{i}}att_{ij}^{q}\;W_{1}^{q}\;h_{j}^{0}\right), \end{array}$$

(7) $$\begin{array}{cc} att_{ij}^{q} & =\frac{\exp\;\left({\left(a^{q}\right)}^{T}\;\sigma_{2}\;\left(W_{1}^{q}\left[\;h_{i}^{0}∥h_{j}^{0}\;\right]\right)\right)}{\sum_{k\in S_{i}}\exp\;\left({\left(a^{q}\right)}^{T}\;\sigma_{2}\;\left(W_{1}^{q}\left[\;h_{i}^{0}∥h_{k}^{0}\;\right]\right)\right)}, \end{array}$$

where $h_{j}^{0}$ denotes the initial feature representation for spot j at the first layer. Q denotes the number of independent attention heads, where $W_{1}^{q}$ denotes the trainable weight matrices for each head q. $att_{ij}^{q}$ refers to the normalized attention coefficients that quantify the interaction between spots i and j, computed via the attention weight vectors $a^{q}$. Non‐linear transformations are governed by activation functions $\sigma_{1}$ and $\sigma_{2}$, which ensure the model captures complex spatial dependencies.

We encode spatial structural information using both the original spatial adjacency matrix $A_{\mathrm{spatial}}$ and the pruned adjacency matrix $A_{\mathrm{pruned}}$. Propagating the first‐layer representation $H^{1}$ through graph convolution under each adjacency yields two intermediate feature matrices, $H_{\mathrm{spatial}}^{1}$ and $H_{\mathrm{pruned}}^{1}$. These representations are subsequently concatenated and projected by the second layer:

(8) $$\begin{array}{cc} h_{i}^{2} & =W_{2}\;\left[\;h_{i}^{1\_\mathrm{spatial}}∥h_{i}^{1\_\mathrm{pruned}}\;\right], \end{array}$$

where $W_{2}$ is a learnable weight matrix. The vector $h_{i}^{2}$ is the embedding of spot i.

The decoder mirrors the encoder architecture and reconstructs the wavelet coefficient representation. Taking $H^{2}$ as input, it first applies a graph attention–based aggregation to propagate neighborhood information, followed by a linear projection that maps the features back to wavelet coefficient space:

(9) $$\begin{array}{cc} h_{i}^{3} & =\sigma_{1}\;\left(\frac{1}{Q}\sum_{q=1}^{Q}\;\sum_{j\in S_{i}}att_{ij}^{q}\;W_{3}^{q}\;h_{j}^{2}\right), \end{array}$$

(10) $$\begin{array}{cc} h_{i}^{4} & =W_{4}\;\left[\;h_{i}^{3\_\mathrm{spatial}}∥h_{i}^{3\_\mathrm{pruned}}\;\right], \end{array}$$

where $W_{3}$ and $W_{4}$ are trainable parameters. $H^{3}$ and $H^{4}$ are the corresponding layer outputs. The final reconstruction is $\hat{C}=H^{4}\in R^{n\times d_{\mathrm{wc}}}$. This design couples attention‐driven neighborhood aggregation with linear projection to achieve graph‐informed reconstruction.

### 4.7 Inverse Wavelet Transform

To more accurately recover spot‐level gene expression patterns, we apply the inverse discrete wavelet transform (IDWT) to both the reconstructed coefficient matrix $\hat{C}$ and masked coefficient matrix $C_{\mathrm{mask}}$. Specifically, we reverse the concatenation defined in Equations (3) and (4) to recover scale‐specific wavelet components. For each spot i (i=1,…,n), a split operator maps the reconstructed coefficient vector ${\hat{c}}_{i}$ to the set $\left(a_{J},d_{J},\text{…},d_{1}\right)$, where J denotes the number of wavelet decomposition levels.

We then perform the IDWT to recover the signal. The single‐level reconstruction is

(11) $$\begin{array}{cc} a_{J-1}\left[r\right] & =\sum_{k=0}^{N-1}\tilde{h}\left[\;r-2k\;\right]\;a_{J}\left[k\right]\;+\;\sum_{k=0}^{N-1}\tilde{g}\left[\;r-2k\;\right]\;d_{J}\left[k\right], \end{array}$$

where $a_{J}$ and $d_{J}$ denote the approximation and detail coefficients at decomposition level J, respectively; $\tilde{h}$ and $\tilde{g}$ are the reconstruction low‐pass and high‐pass filters; N is the signal length at the current level; and r indexes the reconstructed samples. Iteratively applying Equation (11) from level J down to level 1 yields the reconstructed gene expression vector ${\hat{x}}_{i}\in R^{m}$ for spot i.

Stacking the reconstructed vectors ${\hat{x}}_{i}$ across all spots yields the reconstructed expression matrix ${\hat{X}}_{\mathrm{wc}}\in R^{n\times m}$. Applying the same inverse discrete wavelet transform to the masked coefficient matrix $C_{\mathrm{mask}}$ produces ${\hat{X}}_{\mathrm{mask}}$, which preserves low‐frequency approximation components while suppressing high‐frequency noise. Together, the pair $\left({\hat{X}}_{\mathrm{wc}},{\hat{X}}_{\mathrm{mask}}\right)$ provides complementary reconstructions that support robust downstream analysis.

### 4.8 Attention Fusion Mechanism

We integrate ${\hat{X}}_{\mathrm{wc}}$ and ${\hat{X}}_{\mathrm{mask}}$ using an attention‐based fusion module. The reconstruction ${\hat{X}}_{\mathrm{wc}}$ retains high‐frequency details but exhibits attenuated low‐frequency structure, while ${\hat{X}}_{\mathrm{mask}}$ preserves global low‐frequency trends while discarding fine‐scale variations. To balance these complementary properties, we employ spot‐level or gene‐level attention to adaptively weight the two reconstructions, yielding a more accurate and biologically faithful expression profile. Formally,

(12) $$\begin{array}{cc} \gamma^{m} & =\frac{\exp\;\left(a_{m}^{⊤}\left[\;{\hat{X}}_{\mathrm{wc}}∥{\hat{X}}_{\mathrm{mask}}\;\right]\right)}{\sum_{j=1}^{2}\exp\;\left(a_{j}^{⊤}\left[\;{\hat{X}}_{\mathrm{wc}}∥{\hat{X}}_{\mathrm{mask}}\;\right]\right)},\;m\in \left\{1,2\right\}, \end{array}$$

(13) $$\begin{array}{cc} \hat{X} & =\gamma^{1}⊙{\hat{X}}_{\mathrm{wc}}\;+\;\gamma^{2}⊙{\hat{X}}_{\mathrm{mask}}, \end{array}$$

where $\gamma^{m}$ denotes the learned attention coefficient, and the vectors $a_{m}$ are trainable parameters, while ⊙ represents element‐wise multiplication. This fusion mechanism enables complementary integration of low‐frequency structural information and high‐frequency detailed signals.

### 4.9 Loss Function

The objective function of STWave is formulated as a dual‐term loss that jointly optimizes reconstruction fidelity in both the wavelet and gene‐expression domains. The first term measures the reconstruction error of wavelet coefficients, preserving essential multiscale structural features, while the second term enforces accurate recovery of gene expression in the original space. The overall loss L is defined as

(14) $$\min L=\alpha \frac{1}{n}\sum_{i=1}^{n}∥C_{i}-{\hat{C}}_{i}∥+\frac{1}{n}\sum_{i=1}^{n}∥X_{i}-{\hat{X}}_{i}∥,$$

where $C_{i}$ and ${\hat{C}}_{i}$ denote the original and reconstructed wavelet representations for spot i, respectively; $X_{i}$ and ${\hat{X}}_{i}$ are the corresponding original and reconstructed gene expression vectors; n is the total number of spots; and α is a tunable hyperparameter controlling the contribution of the wavelet‐domain reconstruction term.

### 4.10 Training and Inference

After partitioning the original tissue slice into spatial patches, we randomize the patch order and train the model iteratively on each patch. As described above, the spot‐level expression vector is treated as a 1D discrete signal and transformed via a 1D discrete wavelet transform to obtain multiscale coefficients. These coefficients are processed by the autoencoder to generate reconstructed wavelet representations. The inverse discrete wavelet transform is then applied to map the reconstructed coefficients back to gene expression space, yielding a patch‐level reconstructed profile. In parallel, detail coefficients are masked and passed through the same inverse transform to produce a complementary reconstruction emphasizing low‐frequency structure. An attention‐based fusion module adaptively integrates the two reconstructions. Model training is guided by the loss function in Equation (14) and proceeds for a predefined number of epochs.

After training, inference is performed at the patch level to minimize GPU and CPU memory usage. Using the stored spot index sets, patch‐wise predictions are mapped back to their original spatial coordinates on the full tissue slice. This design substantially reduces computational overhead while preserving reconstruction fidelity (Figure 2a and Figure S1). The reconstructed expression profiles, together with the learned graph‐informed embeddings, are subsequently used for downstream tasks such as clustering, visualization, and spatial analysis.

### 4.11 Spatial Clustering

Following the above procedure, each spot is represented by a graph‐informed embedding and a reconstructed gene expression profile. We use the learned embeddings for spatial domain identification with standard clustering algorithms, including Mclust [48], Louvain [9], and Leiden [41]. For tissue slices with manual annotations, we apply Mclust and set the number of clusters to match the annotated domains. When annotations are unavailable or when latent spatial structure is to be discovered, we employ Louvain or Leiden clustering with an appropriately selected resolution. To ensure fair evaluation on annotated benchmarks, we follow the recommended settings for each method and tune the resolution parameters such that the inferred number of domains equals the annotation count, enabling balanced comparisons across methods.

### 4.12 Parameter Sensitivity Analysis

To evaluate the impact of the dual‐domain reconstruction loss weight α (defined in Equation (14)) on model performance, we conducted a sensitivity analysis on the 12 human DLPFC slices. We varied the hyperparameter α from 5 to 30 in increments of 5 to assess its impact on clustering performance. As summarized in Tables S10 and S11, STWave exhibits a clear performance trend, achieving optimal results at α=20. At this setting, the model attains the highest clustering accuracy, with a mean ARI of 0.642 and a mean NMI of 0.654.

Further analysis of mean and variance across different α underscores the robustness of STWave. Although performance peaks at α=20, clustering accuracy remains stable throughout the evaluated range, indicating limited sensitivity to moderate hyperparameter variations. Notably, at α=20, the standard deviation of NMI is minimized (0.045), while the standard deviation of ARI remains low (0.089). This consistently low variance across 12 tissue slices suggests that a fixed setting of α=20 generalizes well across samples without requiring slice‐specific tuning. To further assess whether this setting transfers beyond DLPFC, the same sensitivity analysis was performed on the Xenium human breast cancer dataset and the mouse embryo dataset using three independent random seeds. On the Xenium dataset, α=20 achieved the highest ARI (0.441±0.026) and a comparable NMI (0.549±0.001) across the tested range. On the mouse embryo dataset, α=20 achieved the highest ARI (0.415±0.018) and NMI (0.612±0.001) (Tables S12 and S13). These results demonstrate that STWave exhibits a stable optimization landscape and strong generalization performance.

## Conflicts of Interest

The authors declare no conflicts of interest.

## Supporting information


**Supporting File**: advs77240‐sup‐0001‐SuppMat.pdf.

## Data Availability

All datasets analyzed in this study are publicly available and listed in Supplementary Table S1. Simulated data are provided in the HERGAST [20] GitHub repository (https://github.com/GYQ‐form/HERGAST/tree/main/analysis/simulation_clustering). The human colorectal cancer Visium HD CytAssist gene expression libraries can be downloaded from the 10x Genomics data portal https://www.10xgenomics.com/datasets/visium‐hd‐cytassist‐gene‐expression‐libraries‐of‐human‐crc. The human breast cancer Xenium dataset is available from 10x Genomics (https://www.10xgenomics.com/products/xenium‐in‐situ/preview‐dataset‐human‐breast). The human kidney cancer Xenium dataset can be downloaded from 10x Genomics (https://www.10xgenomics.com/datasets/human‐kidney‐preview‐data‐xenium‐human‐multi‐tissue‐and‐cancer‐panel‐1‐standard). The non–small cell lung cancer CosMx SMI FFPE dataset can be obtained from NanoString: https://nanostring.com/products/cosmx‐spatial‐molecular‐imager/ffpe‐dataset/nsclc‐ffpe‐dataset(Lung‐9‐1). The Stereo‐seq mouse embryo dataset includes raw data [6] at the China National GeneBank Database (CNGBdb, accession CNP0001543) and processed data [49] at the MOSAT database. The human DLPFC 10x Visium dataset is accessible through the Bioconductor package spatialLIBD [50]. The source code for STWave is available GitHub at https://github.com/TaoJiang999/STWave.
