# Supplementary material for: STWave: Fine‐Scale Spatial Structure Discovery in Microscopic‐Resolution Spatial Transcriptomics via Patchwise Wavelet Graphs
Source: Adv Sci (Weinh). 2026 Aug 19:e77240. Online ahead of print. doi: 10.1002/advs.77240 (PMC13487875; doi:10.1002/advs.77240)
Supplement: Supplementary file 1 [file ADVS-9999-e77240-s001.pdf]

Supplementary Information

1 Supplementary Notes

1.1 Parameter setting of methods

Seurat

Seurat[1] is a computational method that integrates single cell RNA sequencing data with a limited set of in situ landmark gene maps to reconstruct cellular spatial positions. The Scanpy pipeline was followed according to the spatial data tutorial. Leiden clustering was applied, and the resolution parameter was tuned to match the number of annotated domains when annotations were available; when annotations were not available, the *resolution* was set to 0.6, 0.8, and 1.0. All other parameters were kept at their default settings.

Scanpy

Scanpy[2] is a comprehensive toolbox for the processing and analysis of single cell RNA sequencing data and spatial transcriptomics (ST) data. The Scanpy pipeline was implemented following the spatial data tutorial. Dimensionality reduction with *scanpy.pp.pca* was performed to 50 principal components. Leiden clustering was applied, and the resolution parameter was set to 0.6, 0.8, and 1.0. All other parameters were kept at their default settings.

stLearn

stLearn[3] leverages spatial coordinates, tissue morphology, and gene expression to analyze ST data with a normalization scheme based on Spatial Morphology Enrichment. The recommended pipeline in the official tutorial includes DLPFC cases and was therefore adopted for benchmarking. Specifically, *st.pp.tiling* was used to partition the tissue into patches; *st.pp.extract_feature* was used to derive morphology and intensity features; *st.em.run_pca* was applied with 50 principal components; *st.spatial.SME.SME_normalize* was executed with default weights; and *st.tl.clustering.kmeans* was used for clustering with the number of clusters set to the annotated domain count when available. All remaining parameters were kept at their default settings.

SpaGCN

SpaGCN[4] integrates gene expression, spatial coordinates, and morphology with a graph convolutional network to identify spatial domains and prioritize domain specific genes. The spatial clustering analysis was conducted in accordance with the SpaGCN tutorial; *SpaGCN.SpaGCN.predict* was used with the number of clusters set to the annotation label count, and all other parameters were kept at their default settings.

BayesSpace

BayesSpace[5], an Bayesian model was used to integrate local neighborhood information, spatial clustering was performed, and Visium and related datasets were upgraded to subspot resolution without external single cell references. The analysis was conducted in R. *runPCA* was applied with 50 principal components. *spatialCluster* was executed with *gamma* set to 3 and the number of clusters matched to the annotation label count. All remaining parameters were kept at default settings.

STAGATE

STAGATE[6] is a graph attention autoencoder for learning low dimensional embeddings. The procedure consists of three stages: construction of a spatial neighbor network, perception of cell type aware

networks, and an encode–decode phase. The analysis was conducted in accordance with the official STAGATE tutorial, and all other parameters were kept at their default settings. For datasets with manual annotations, Mclust was applied and the number of clusters was set to the annotation label count; otherwise, Louvain clustering was used.

CCST

CCST[7] integrates gene expression and spatial neighborhood information into a graph convolutional network to learn graph informed low dimensional embeddings, thereby improving unsupervised clustering and revealing functional cell subpopulations with enhanced biological interpretability. Workflow: a mixed adjacency matrix is constructed by combining spatial proximity and expression similarity with a balancing parameter lambda; a Deep Graph Infomax style self supervised objective trains a graph convolutional network on the original graph and a perturbed graph to obtain embeddings; the embeddings are reduced by PCA and clustered with k-means to produce cell groups and subgroups. Implementation details: according to the official CCST README tutorial, $lambda\_I$ was set to 0.3 and num_epoch was set to 5000, while all remaining parameters were kept at default settings.

SpaceFlow

SpaceFlow[8] is a deep learning framework that leverages embedded spatial consistency to learn low dimensional embeddings for ST and to support downstream clustering and trajectory analysis. The workflow consists of constructing a spatial representation graph, regularizing the encoder with a spatial consistency term, and training iteratively until convergence. The implementation followed the official tutorial. In the *train* function, z_dim was set to 50, *epochs* was set to 1000, and $spatial\_regularization\_strength$ was set to 0.1. All remaining parameters were kept at default settings.

SpatialPCA

SpatialPCA[9] is a spatially aware dimensionality reduction method that extracts a low dimensional representation of ST data and performs clustering. SpatialPCA was implemented in accordance with the official tutorial. In $SpatialPCA\_EstimateLoading$, the parameter $SpatialPCnum$ was set to 20. In $walktrap\_clustering$, the parameter knearest was set to 70. All remaining parameters were kept at their default settings.

conST

conST[10] integrates gene expression, spatial coordinates, and histology to learn a graph informed embedding. The workflow constructs a k nearest neighbor graph, pretrains an autoencoder and a variational graph autoencoder, and applies contrastive learning, thereby enabling clustering and related analyses. The implementation followed the official conST notebook tutorial. For datasets with manual annotations, the number of clusters was fixed to the annotation label count. The function $res\_search\_fixed\_clus$ was used to select the resolution that matches this cluster number, after which Leiden clustering was performed. All remaining parameters were kept at default settings.

PAST

PAST[11] addresses the challenge of jointly leveraging reference priors and local spatial dependence in ST. The workflow constructs a k nearest neighbor spatial graph, introduces a Bayesian Neural Network prior to disentangle shared and dataset specific variation, aggregates neighborhood information with self attention and a graph residual module, obtains low dimensional embeddings via a variational graph autoencoder with reparameterization, reconstructs expression, and trains with a Ripple Walk Sampler for subgraph scalability; the embeddings are used for clustering and visualization. Implementation followed the official tutorial with d_lat set to 50 and *epochs* set to 50. When manual annotations were available, Mclust was applied with the number of clusters equal to the label count; otherwise Louvain clustering was used.

GraphST

GraphST[12] is a graph self supervised contrastive learning framework. In the clustering module, three steps are executed: genes are randomly shuffled to create perturbations, a GNN encoder is constructed

to obtain spot embeddings, and contrastive learning is applied to strengthen agreement between each spot embedding and embeddings of its spatial neighbors. For implementation, *dim_output* was kept at the default value 64, and *GraphST.utils.clustering* was used with the argument method set to Mclust, Louvain, or Leiden to perform clustering. All other parameters were kept at their default settings.

spaVAE

spaVAE[13] is a spatial variational autoencoder with dependency awareness that models count data in a probabilistic manner while capturing spatial correlations. The spaVAE pipeline was implemented according to the official GitHub tutorials. The parameter KL_loss was set to 0.025 and $Normal\_dim$ was set to 8. All remaining parameters were kept at their default settings.

SEDR

SEDR[14] addresses joint modeling of gene expression and spatial context with denoising and yields a unified embedding for clustering, trajectory inference, data integration, and imputation. Workflow: a masked autoencoder compresses expression features; a k nearest neighbor spatial graph is constructed from coordinates and embedded with a variational graph autoencoder; the two latent representations are concatenated; joint reconstruction loss and a Deep Embedding Clustering objective optimize the representation. Implementation followed the official tutorial: the network was instantiated with $SEDR.Sedr$, and $SEDR.mclust\_R$ was applied with *n_clusters* set to the annotation label count when available; all other parameters were kept at default settings.

MuCoST

MuCoST[15] addresses the limitation that local neighborhood assumptions fail to capture long range coexpression, thereby improving spatial domain delineation. A spatial adjacency graph, a gene coexpression graph, and a shuffled control graph are constructed. A GCN autoencoder is trained with an InfoNCE objective to align representations from the spatial and coexpression graphs while repelling the random view, and clustering on the spatial view embedding is performed to obtain spatial domains. The official tutorial was followed. The parameter *mode_his* was set to *noh*, *n_domain* was set to the annotation label count for labeled datasets, *epoch* was set to 1000, and Mclust was applied for clustering. All remaining parameters were kept at default settings.

SpaSEG

SpaSEG[16] is designed to robustly delineate spatial domains across platforms, detect spatially variable genes, and achieve cross slice alignment for ST. PCA features are reshaped into an image like tensor, a convolutional neural network is trained with a joint objective of cross entropy loss and edge strength loss for iterative segmentation, spatial domains are obtained, and subsequent analyses include SVG detection, cell-cell interaction inference, and multi slice integration. Implementation followed the official tutorial. In *sc_processing*, the parameter *compons* was set to 15. The function *_add_seg_label* was used to generate clustering labels. All remaining parameters were kept at their default settings.

DeepGFT

DeepGFT[17] addresses noise in ST and the limitation of relying only on spot level proximity, thereby improving spatial domain detection and denoising. The workflow constructs a gene coexpression graph and a spatial adjacency graph, performs a graph Fourier transform followed by low pass filtering, learns embeddings with a graph attention autoencoder, fuses reconstructions with attention, applies an inverse transform, and clusters the resulting embeddings to obtain spatial domains. Implementation followed the official tutorial. The function $f2s\_gene$ was used with *gene_signal* set to 1500, and $f2s\_spot$ was used with *spot_signal* set to 1500. Mclust was applied with the number of clusters matched to the annotation label count. All remaining parameters were kept at default settings.

HERGAST

HERGAST[18] is designed for spatial structure identification and signal amplification in ultra large and ultra high resolution ST. The slice is partitioned into small patches and trained iteratively;

a heterogeneous graph attention network that integrates spatial proximity and expression similarity is constructed; embeddings are obtained with an encoder and reconstructed with a linear decoder; whole slide inference is then performed and the embeddings are clustered to delineate spatial domains. Implementation followed the official tutorial: in $HERGAST.Train\_HERGAST$ the parameter $num\_batch\_x\_y$ was set to $round(\sqrt{n/10000})$ and $dim\_reduction$ was set to HVG, and all remaining parameters were kept at default settings.

STWave

STWave is proposed to address excessive GPU and CPU consumption on ultra large ST datasets. A divide and sample subgraph strategy is adopted. The tissue is partitioned into patches by spatial coordinates. Subgraphs for each patch are sampled with the Python PYG library. Training is performed iteratively on each patch. Inference is also conducted on each patch, and predictions are mapped back to the full dataset through stored indices. GPU memory usage was observed to remain low across the evaluated data scales. Given that raw ST expression is discrete, high dimensional, and sparse with many zeros, each spot expression vector is treated as a one dimensional discrete signal and a wavelet transform is applied to obtain multi scale coefficients. Two reconstruction branches produce gene expression profiles. An attention mechanism fuses the reconstructions and yields a high fidelity recovery of the original expression profile. For the patch grid size $m \times m$, m was set to $round(\sqrt{n/10000})$. Clustering was performed with Mclust or Louvain.

1.2 Metrics used in all experiments

In addition to spatial clustering visualization, the performance of STWave on ST datasets was evaluated using the following criteria:

Clustering metric definitions

For all label-based evaluations, let $C = \{C_1, \ldots, C_r\}$ denote the reference classes and $K = \{K_1, \ldots, K_s\}$ denote the predicted clusters. The contingency table is denoted by $N = \{n_{ck}\}$, where $n_{ck} = |C_c \cap K_k|$, $n_{c\cdot} = \sum_k n_{ck}$, $n_{\cdot k} = \sum_c n_{ck}$, and $n = \sum_{c,k} n_{ck}$ is the total number of spots. Larger values indicate better agreement for all metrics reported below, except that chance-adjusted metrics can take values below 0 when agreement is worse than expected by chance.

Adjusted Rand Index

The Rand Index (RI) evaluates pairwise agreement between two partitions[19]. The Adjusted Rand Index (ARI) corrects RI for chance agreement and is widely used for comparing clustering results with reference annotations[20]. It is defined as

$$\mathrm{ARI} = \frac{\sum_{c,k}\binom{n_{ck}}{2} - \frac{\sum_c \binom{n_{c\cdot}}{2}\sum_k \binom{n_{\cdot k}}{2}}{\binom{n}{2}}}{\frac{1}{2}\left[\sum_c \binom{n_{c\cdot}}{2} + \sum_k \binom{n_{\cdot k}}{2}\right] - \frac{\sum_c \binom{n_{c\cdot}}{2}\sum_k \binom{n_{\cdot k}}{2}}{\binom{n}{2}}}. \tag{1}$$

ARI equals 1 for identical partitions, is close to 0 for random agreement, and may be negative when the agreement is lower than expected by chance.

Normalized Mutual Information

Normalized Mutual Information (NMI) measures the shared information between reference classes and predicted clusters after normalization[21]. Let

$$I(C;K) = \sum_{c=1}^{r}\sum_{k=1}^{s} \frac{n_{ck}}{n} \log \frac{n\, n_{ck}}{n_{c\cdot} n_{\cdot k}}, \tag{2}$$

$$H(C) = -\sum_{c=1}^{r} \frac{n_{c\cdot}}{n} \log \frac{n_{c\cdot}}{n}, \quad H(K) = -\sum_{k=1}^{s} \frac{n_{\cdot k}}{n} \log \frac{n_{\cdot k}}{n}. \tag{3}$$

Using the arithmetic normalization adopted in the experiments, NMI is

$$\mathrm{NMI} = \frac{I(C;K)}{\left[H(C)+H(K)\right]/2}. \tag{4}$$

NMI ranges from 0 to 1, where higher values indicate stronger information-theoretic concordance between predicted clusters and reference classes.

Adjusted Mutual Information

Adjusted Mutual Information (AMI) further adjusts mutual information for chance agreement[22]. With $\mathbb{E}[I(C;K)]$ denoting the expected mutual information under a random model with fixed cluster sizes, AMI is

$$\mathrm{AMI} = \frac{I(C;K) - \mathbb{E}[I(C;K)]}{\left[H(C)+H(K)\right]/2 - \mathbb{E}[I(C;K)]}. \tag{5}$$

AMI equals 1 for identical partitions and is close to 0 for random labelings, making it useful when the number or size distribution of clusters differs across methods.

Homogeneity, Completeness, and V-measure

Homogeneity, Completeness, and V-measure are conditional entropy-based external clustering metrics[23]. Homogeneity measures whether each predicted cluster contains samples from a single reference class:

$$h = \begin{cases} 1, & H(C)=0, \\ 1-\frac{H(C|K)}{H(C)}, & H(C)>0, \end{cases} \tag{6}$$

where

$$H(C|K) = -\sum_{c=1}^{r}\sum_{k=1}^{s} \frac{n_{ck}}{n} \log \frac{n_{ck}}{n_{\cdot k}}. \tag{7}$$

Completeness measures whether all samples from the same reference class are assigned to the same predicted cluster:

$$c = \begin{cases} 1, & H(K)=0, \\ 1-\frac{H(K|C)}{H(K)}, & H(K)>0, \end{cases} \tag{8}$$

where

$$H(K|C) = -\sum_{c=1}^{r}\sum_{k=1}^{s} \frac{n_{ck}}{n} \log \frac{n_{ck}}{n_{c\cdot}}. \tag{9}$$

V-measure combines Homogeneity and Completeness as their weighted harmonic mean:

$$V_\beta = \frac{(1+\beta)hc}{\beta h + c}. \tag{10}$$

The experiments used $\beta = 1$, giving equal weight to Homogeneity and Completeness.

Fowlkes–Mallows Index

The Fowlkes–Mallows Index (FMI) measures pairwise clustering agreement by combining pairwise precision and recall[24]. Let

$$\mathrm{TP} = \sum_{c,k}\binom{n_{ck}}{2}, \quad \mathrm{FP} = \sum_{k}\binom{n_{\cdot k}}{2} - \mathrm{TP}, \quad \mathrm{FN} = \sum_{c}\binom{n_{c\cdot}}{2} - \mathrm{TP}. \tag{11}$$

Then

$$\mathrm{FMI} = \frac{\mathrm{TP}}{\sqrt{(\mathrm{TP}+\mathrm{FP})(\mathrm{TP}+\mathrm{FN})}}. \tag{12}$$

FMI ranges from 0 to 1, with larger values indicating stronger pairwise consistency between predicted clusters and reference classes.

Purity

Purity measures the extent to which each predicted cluster is dominated by a single reference class and is commonly used in clustering evaluation[25]. It is defined as

$$\mathrm{Purity}(C,K) = \frac{1}{n}\sum_{k=1}^{s}\max_{c} n_{ck}. \tag{13}$$

Purity ranges from 0 to 1, and a larger value indicates that predicted clusters contain more label-consistent spots. Because Purity can increase when many small clusters are produced, it was used as a complementary metric rather than as the sole evaluation criterion.

Implementation

ARI, NMI, AMI, Homogeneity, Completeness, V-measure, and FMI were computed using the corresponding functions in `sklearn.metrics`. Purity was computed from the contingency table according to Equation 13.

Region-specific relative marker signal gain

To quantitatively assess the enhancement of marker signals within selected H&E-defined regions after amplification, we calculated a region-specific relative marker signal gain. Because raw and amplified expression maps may have different numerical scales, the raw and amplified maps were independently normalized to the range $[0, 1]$ for each marker gene using robust min–max scaling based on the 1st and 99th percentiles. For gene g and spot i, the normalized expression value in map $m \in \{\mathrm{raw}, \mathrm{amp}\}$ was defined as

$$\tilde{x}_{i,g}^{(m)} = \mathrm{clip}\left(\frac{x_{i,g}^{(m)} - Q_{0.01}\left(X_{\cdot,g}^{(m)}\right)}{Q_{0.99}\left(X_{\cdot,g}^{(m)}\right) - Q_{0.01}\left(X_{\cdot,g}^{(m)}\right) + \epsilon}, 0, 1\right), \tag{14}$$

where $x_{i,g}^{(m)}$ denotes the expression value of gene g at spot i in map m, $X_{\cdot,g}^{(m)}$ denotes the expression values of gene g across all spots in map m, $Q_{0.01}$ and $Q_{0.99}$ denote the 1st and 99th percentiles, respectively, and ϵ is a small constant used to avoid division by zero. The function $\mathrm{clip}(\cdot, 0, 1)$ restricts normalized values to the interval $[0, 1]$.

For each H&E-defined region R_k, the mean normalized marker signal of gene g was calculated as

$$S_{k,g}^{(m)} = \frac{1}{|R_k|}\sum_{i\in R_k}\tilde{x}_{i,g}^{(m)}, \tag{15}$$

where $|R_k|$ is the number of spots within region R_k. The region-specific relative marker signal gain for gene g in region R_k was defined as

$$RSG_{k,g} = \frac{S_{k,g}^{(\mathrm{amp})} + \epsilon}{S_{k,g}^{(\mathrm{raw})} + \epsilon}. \tag{16}$$

A value of $RSG_{k,g} > 1$ indicates that the amplified map has a higher mean normalized marker signal within the corresponding H&E-defined region than the raw map.

For a marker gene set G_k associated with region R_k, the region-level relative signal gain was summarized by averaging the log-transformed gains across marker genes:

$$\log_2 RSG_{k,G_k} = \frac{1}{|G_k|}\sum_{g\in G_k}\log_2 RSG_{k,g}, \tag{17}$$

and the corresponding fold gain was obtained as

$$RSG_{k,G_k} = 2^{\log_2 RSG_{k,G_k}}. \tag{18}$$

This gene-set summary quantifies the overall region-specific relative marker signal gain within each H&E-defined region. The RSG metric was used to evaluate relative normalized signal enhancement and was not interpreted as an absolute expression fold change.

Embedding structure preservation

To provide a clustering-agnostic evaluation of the learned representation, embedding quality was assessed by quantifying the preservation of transcriptomic and spatial structures. These measures follow the quantitative evaluation framework used for spatially resolved embeddings in SpaSNE[26]. Let $X \in \mathbb{R}^{n\times p}$ denote the reference expression representation of n spatial locations, $S \in \mathbb{R}^{n\times 2}$ denote their spatial coordinates, and $Z^{(m)} \in \mathbb{R}^{n\times d}$ denote the embedding produced by method m. For locations i and j, the corresponding pairwise Euclidean distances were defined as

$$\begin{aligned} d_{ij}^{X} &= \|X_i - X_j\|_2, \\ d_{ij}^{S} &= \|S_i - S_j\|_2, \\ d_{ij}^{Z,(m)} &= \|Z_i^{(m)} - Z_j^{(m)}\|_2. \end{aligned} \tag{19}$$

Gene-expression distance preservation, r_g, and spatial-distance preservation, r_s, were calculated as the Pearson correlations between the upper-triangular elements of the corresponding distance matrices:

$$\begin{aligned} r_g^{(m)} &= \mathrm{corr}_{\mathrm{P}}\left[\mathrm{vec}_{\triangle}(D^X), \mathrm{vec}_{\triangle}(D^{Z,(m)})\right], \\ r_s^{(m)} &= \mathrm{corr}_{\mathrm{P}}\left[\mathrm{vec}_{\triangle}(D^S), \mathrm{vec}_{\triangle}(D^{Z,(m)})\right]. \end{aligned} \tag{20}$$

Both measures range from -1 to 1, with larger values indicating better preservation of the global transcriptomic or spatial distance structure, respectively.

Local neighborhood preservation was evaluated using Trustworthiness, an established measure of dimensionality-reduction quality[27, 28]. For location i, let $U_k(i)$ contain locations that appear among its k nearest neighbors in the embedding but not among its k nearest neighbors in the reference space, and let $r(i,j)$ be the rank of location j relative to i in the reference space. Trustworthiness was calculated as

$$T(k) = 1 - \frac{2}{nk(2n-3k-1)} \sum_{i=1}^{n} \sum_{j \in U_k(i)} \left[r(i,j) - k\right]. \tag{21}$$

Using the expression representation and spatial coordinates as the respective reference spaces yielded transcriptomic Trustworthiness and spatial Trustworthiness:

$$T_g^{(m)}(k) = T\left(X, Z^{(m)}; k\right), \qquad T_s^{(m)}(k) = T\left(S, Z^{(m)}; k\right). \tag{22}$$

T_g and T_s range from 0 to 1, with larger values indicating more faithful preservation of transcriptomic and spatial neighborhoods, respectively. The neighborhood size was set to $k = 15$ in the DLPFC analysis. For each metric, STWave and STWave without the wavelet-transform module were compared across the 12 matched DLPFC slices using a two-sided Wilcoxon signed-rank test. The four resulting p values were adjusted using the Benjamini–Hochberg procedure.

Reconstruction fidelity metrics

Reconstruction fidelity within the Xenium human breast cancer regions of interest (ROIs) was evaluated using correlation- and error-based measures that are commonly used in spatial gene-expression denoising, prediction, and reconstruction studies[29–31]. Let $x_{i,g}$ and $\hat{x}_{i,g}^{(m)}$ denote the observed and reconstructed expression values, respectively, for marker gene g in cell i and method m. Both were evaluated on the same library-size-normalized and log-transformed scale. For each ROI–marker pair, the evaluation subset was $I_g = \{i : x_{i,g} > 0\}$, comprising the $N_g = |I_g|$ cells with detected raw

expression. The Pearson correlation coefficient (PCC) was defined as

$$\mathrm{PCC}_g^{(m)} = \frac{\sum_{i \in I_g} (x_{i,g} - \bar{x}_g)(\hat{x}_{i,g}^{(m)} - \bar{\hat{x}}_g^{(m)})}{\sqrt{\sum_{i \in I_g} (x_{i,g} - \bar{x}_g)^2} \sqrt{\sum_{i \in I_g} (\hat{x}_{i,g}^{(m)} - \bar{\hat{x}}_g^{(m)})^2}}, \tag{23}$$

where $\bar{x}_g$ and $\bar{\hat{x}}_g^{(m)}$ are the corresponding means over I_g. Spearman's rank correlation coefficient was calculated as the Pearson correlation between the ranked expression values:

$$\rho_g^{(m)} = \mathrm{corr}_{\mathrm{P}} \left[\mathrm{rank}(x_{\cdot,g}), \mathrm{rank}(\hat{x}_{\cdot,g}^{(m)}) \right], \qquad i \in I_g. \tag{24}$$

PCC and Spearman's ρ range from -1 to 1, and larger values indicate greater concordance between the observed and reconstructed expression patterns.

The root mean squared error (RMSE) and mean absolute error (MAE) were defined as

$$\begin{aligned} \mathrm{RMSE}_g^{(m)} &= \sqrt{\frac{1}{N_g} \sum_{i \in I_g} \left(\hat{x}_{i,g}^{(m)} - x_{i,g} \right)^2}, \\ \mathrm{MAE}_g^{(m)} &= \frac{1}{N_g} \sum_{i \in I_g} \left| \hat{x}_{i,g}^{(m)} - x_{i,g} \right|. \end{aligned} \tag{25}$$

To facilitate comparisons across marker genes with different expression ranges, the errors were additionally normalized by the robust raw-expression range

$$A_g = Q_{0.99}(x_{\cdot,g}) - Q_{0.01}(x_{\cdot,g}) + \epsilon, \qquad i \in I_g, \tag{26}$$

yielding

$$\mathrm{NRMSE}_g^{(m)} = \frac{\mathrm{RMSE}_g^{(m)}}{A_g}, \qquad \mathrm{NMAE}_g^{(m)} = \frac{\mathrm{MAE}_g^{(m)}}{A_g}. \tag{27}$$

RMSE, MAE, NRMSE, and NMAE are nonnegative, and smaller values indicate higher fidelity to the observed expression values. These measures quantify consistency with the observed data and were not interpreted as accuracy against an unobserved noise-free ground truth.

2 Supplementary Figures

Figure S. 1. Resource consumption and runtime were assessed on simulated datasets.
Figure S. 2. Clustering accuracy metrics on simulated datasets were evaluated across methods and data scales.
Figure S. 3. Spatial clustering results of STWave and comparator methods are presented on the simulated dataset with 80, 000 spots.
Figure S. 4. Spatial clustering results of STWave and comparator methods are presented on the simulated dataset with 160, 000 spots.
Figure S. 5. Spatial clustering results of STWave and comparator methods are presented on the simulated dataset with 240, 000 spots.
Figure S. 6. Spatial clustering results of STWave and comparator methods are presented on the simulated dataset with 320, 000 spots.
Figure S. 7. Spatial clustering results of STWave and comparator methods are presented on the simulated dataset with 480, 000 spots.
Figure S. 8. Spatial clustering results of STWave and comparator methods are presented on the simulated dataset with 640, 000 spots.
Figure S. 9. Spatial clustering visualizations on the Xenium human breast cancer dataset.
Figure S. 10. Spatial expression maps on the Xenium human breast cancer dataset.
Figure S. 11. Spatial expression in the selected region is shown for the raw signal and for the signal amplified by STWave on the Xenium human breast cancer dataset.

Figure S. 12. Performance of STWave and comparator methods was evaluated, and genes with high expression in two specific spatial domains were visualized on the Xenium human breast cancer dataset.
Figure S. 13. Spatial clustering visualizations of STWave and comparator methods were generated on the CosMx SMI human lung cancer dataset.
Figure S. 14. Performance of STWave was evaluated on the CosMx SMI human lung cancer dataset.
Figure S. 15. Spatial clustering visualizations of STWave and comparator methods were generated on the mouse embryo E16.5 dataset.
Figure S. 16. Selected spatial domains identified by STWave and the corresponding reference annotations are displayed separately.
Figure S. 17. Performance of STWave and comparator methods was evaluated, and genes with high expression in several specific spatial domains were visualized on the mouse embryo E16.5 dataset.
Figure S. 18. Spatial clustering visualizations produced by STWave, together with reference annotation visualizations, were generated on three mouse embryo datasets.
Figure S. 19. Clustering accuracy metrics on the 10x Visium DLPFC dataset.
Figure S. 20. Spatial clustering visualizations on the human DLPFC dataset were generated for STWave and other methods.
Figure S. 21. PAGA graphs and UMAP visualizations on the first six human DLPFC slices were generated for STWave and other methods.
Figure S. 22. PAGA graphs and UMAP visualizations on the last six human DLPFC slices were generated for STWave and other methods.
Figure S. 23. Distributions of ARI and NMI on the DLPFC dataset under different randomized gene orders.
Figure S. 24. Spatial visualization and spatial expression analysis on the Xenium human kidney cancer dataset.
Figure S. 25. Microenvironmental comparison of calcified regions in the Visium HD human colorectal cancer dataset.
Figure S. 26. Spatial and functional characterization of the $CXCL8$-high inflammatory region in the Visium HD human colorectal cancer dataset.
Figure S. 27. Sensitivity of STWave to the number of highly variable genes on the DLPFC dataset.
Figure S. 28. Difference heatmap between STWave and STWave without the wavelet transform on the DLPFC and Xenium human breast cancer datasets.
Figure S. 29. Comprehensive clustering metric comparison on the human DLPFC dataset.
Figure S. 30. Clustering-agnostic evaluation of the wavelet-transform module on the Xenium human breast cancer and human DLPFC datasets.

3 Supplementary Tables

Table S. 1. ST datasets used in our study.

Table S. 2. GPU memory usage of STWave and comparator methods across simulated datasets at multiple scales.

Table S. 3. CPU memory usage of STWave and comparator methods across simulated datasets at multiple scales.

Table S. 4. Runtime of STWave and comparator methods across simulated datasets at multiple scales.

Table S. 5. Spatial clustering performance was evaluated by comparing STWave with other methods using the Adjusted Rand Index on ten simulated datasets.

Table S. 6. Spatial clustering performance was evaluated by comparing STWave with other methods using the Normalized Mutual Information on ten simulated datasets

Table S. 7. GPU usage, CPU usage, Training time, and Adjusted Rand Index performance of STWave were evaluated on three simulated datasets across multiple patch configurations with varying spot counts.

Table S. 8. Spatial clustering performance was evaluated by comparing STWave with other methods using the Adjusted Rand Index on twelve human DLPFC slices.

Table S. 9. Spatial clustering performance was evaluated by comparing STWave with other methods using the Normalized Mutual Information on twelve human DLPFC slices.

Table S. 10. Adjusted Rand Index performance of STWave was evaluated on twelve human DLPFC slices across different values of the loss weight α.

Table S. 11. Normalized Mutual Information performance of STWave was evaluated on twelve human DLPFC slices across different values of the loss weight α.

Table S. 12. Mean and standard deviation of ARI and NMI for STWave on the Xenium human breast cancer dataset across different α values from three independent runs with different random seeds.

Table S. 13. Mean and standard deviation of ARI and NMI for STWave on the mouse embryo dataset across different α values from three independent runs with different random seeds.

Table S. 14. Clustering performance on boundary and interior regions under different boundary ranges using the Xenium human breast cancer dataset.

Table S. 15. Quantification of region-specific relative marker signal gain on the Xenium human breast cancer dataset.

Table S. 16. Number of clusters produced by each method on the CosMx SMI human lung cancer dataset under different Leiden resolution parameters.

Table S. 17. Evaluation of clustering metrics for STWave and STWave without the wavelet transform on the DLPFC and Xenium human breast cancer datasets.

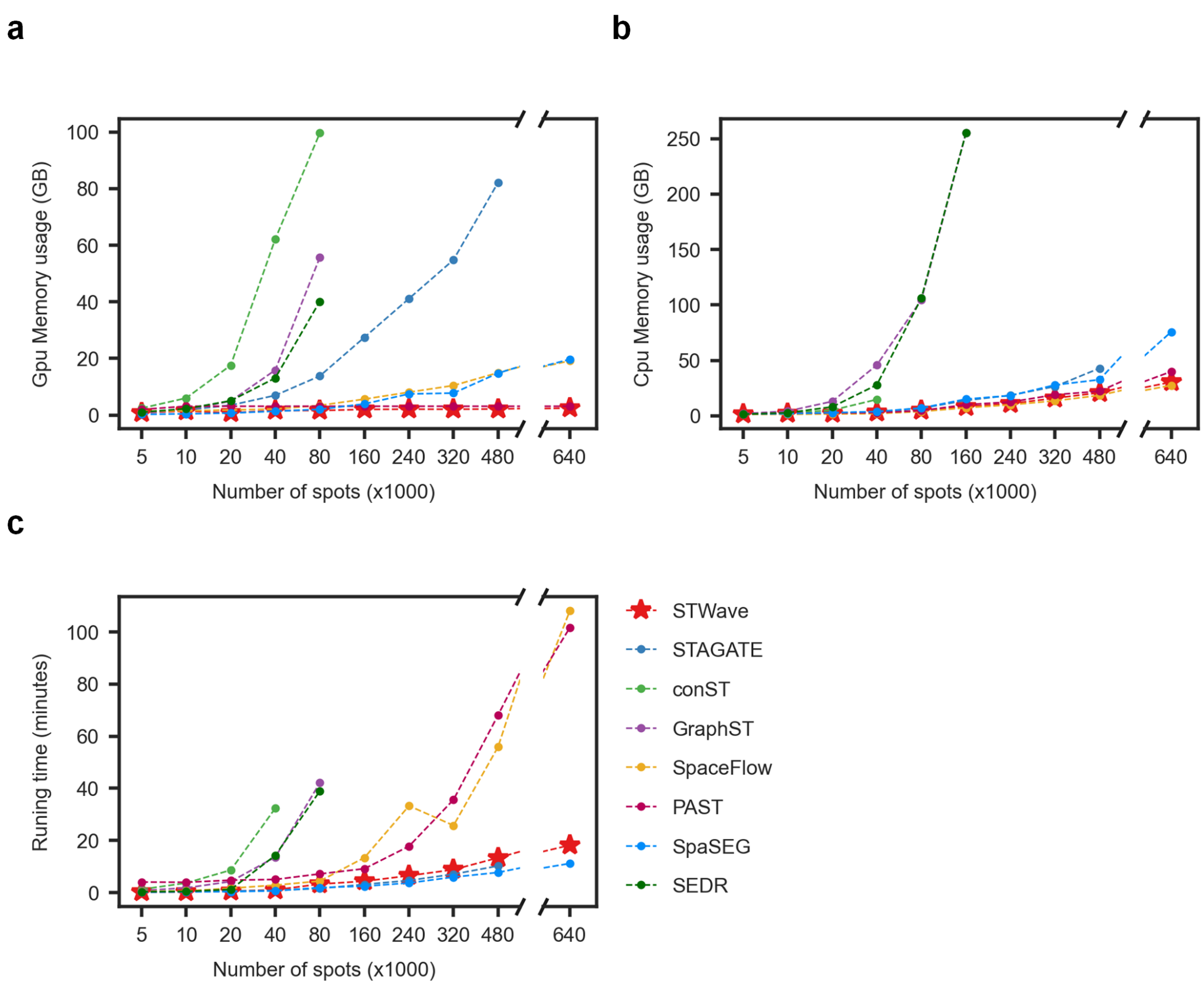

Figure S. 1 Resource consumption and runtime were assessed on simulated datasets. a. GPU usage across methods at multiple data scales. **b.** CPU usage across methods at multiple data scales. **c.** Runtime across methods at multiple data scales.

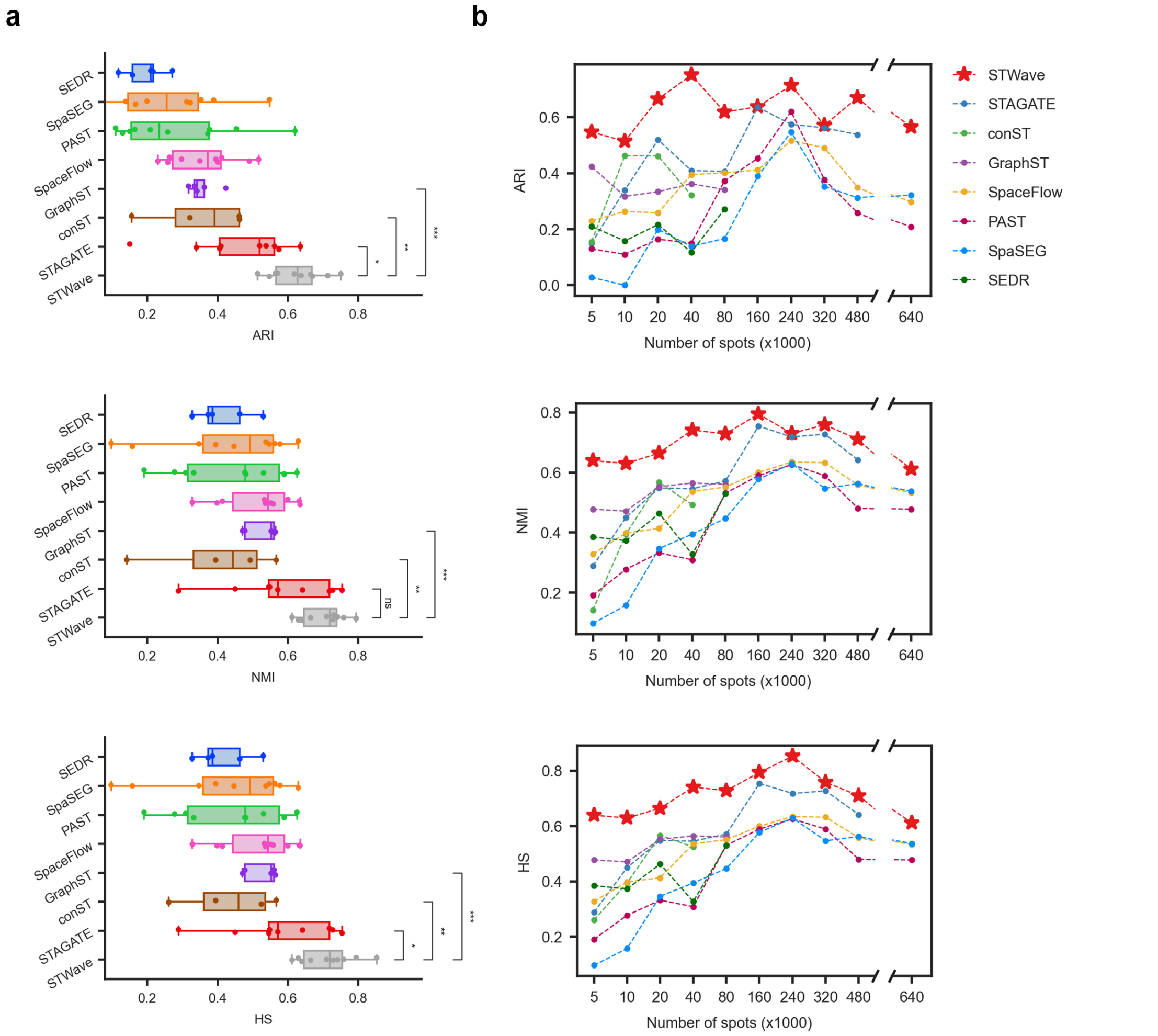

Figure S. 2 Clustering accuracy metrics on simulated datasets were evaluated across methods and data scales. a. Box plots of Adjusted Rand Index (top), Normalized Mutual Information (middle), and Homogeneity Score (bottom) across methods at multiple simulation scales. Statistical significance indicated as: ns $p \geq 0.05$, $^{*}p < 0.05$, $^{**}p < 0.01$, $^{***}p < 0.001$, $^{****}p < 0.0001$. **b.** Line plots of Adjusted Rand Index (top), Normalized Mutual Information (middle), and Homogeneity Score (bottom) across methods at multiple simulation scales.

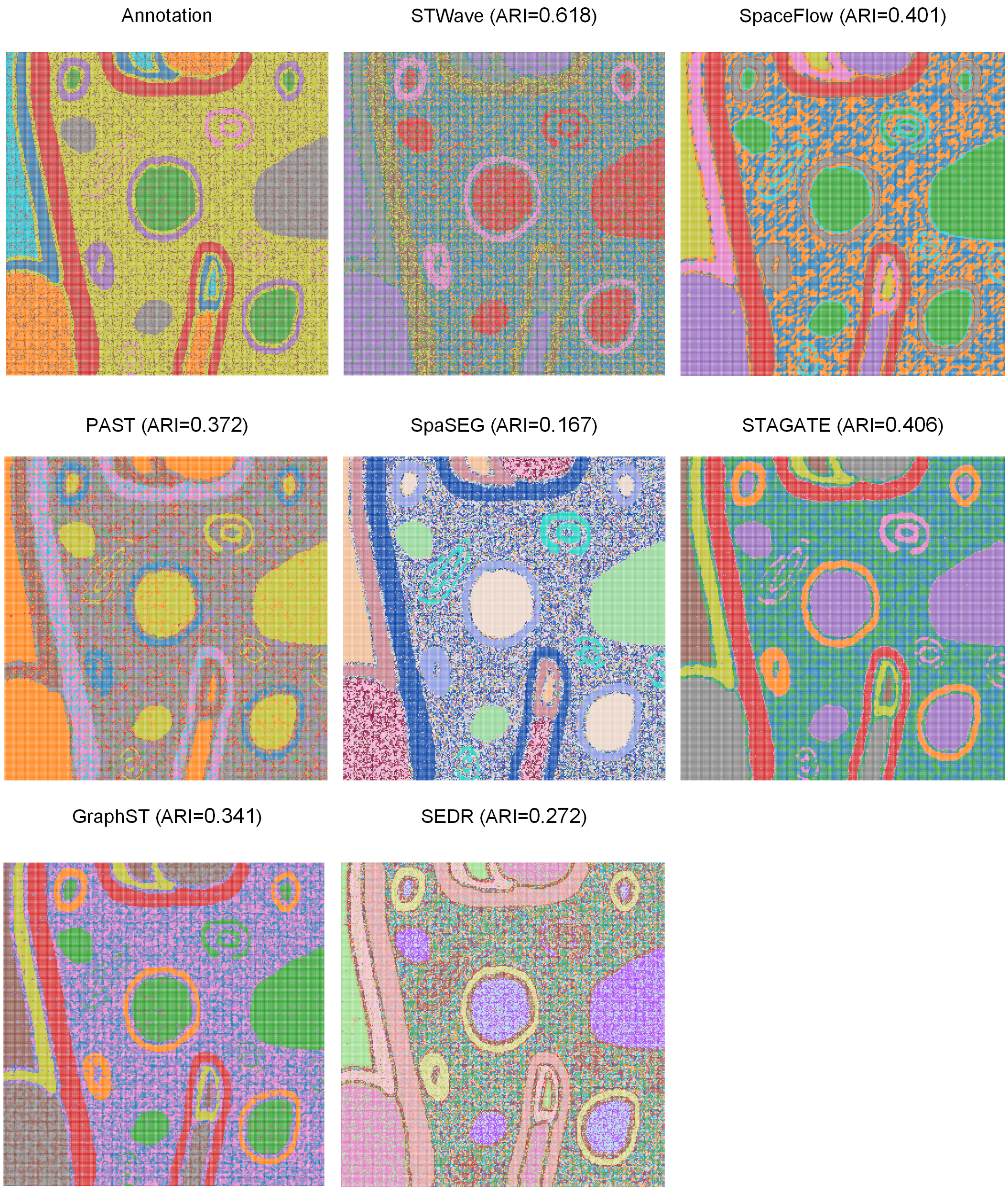

Figure S. 3 Spatial clustering results of STWave and comparator methods are presented on the simulated dataset with $80,000$ **spots.**

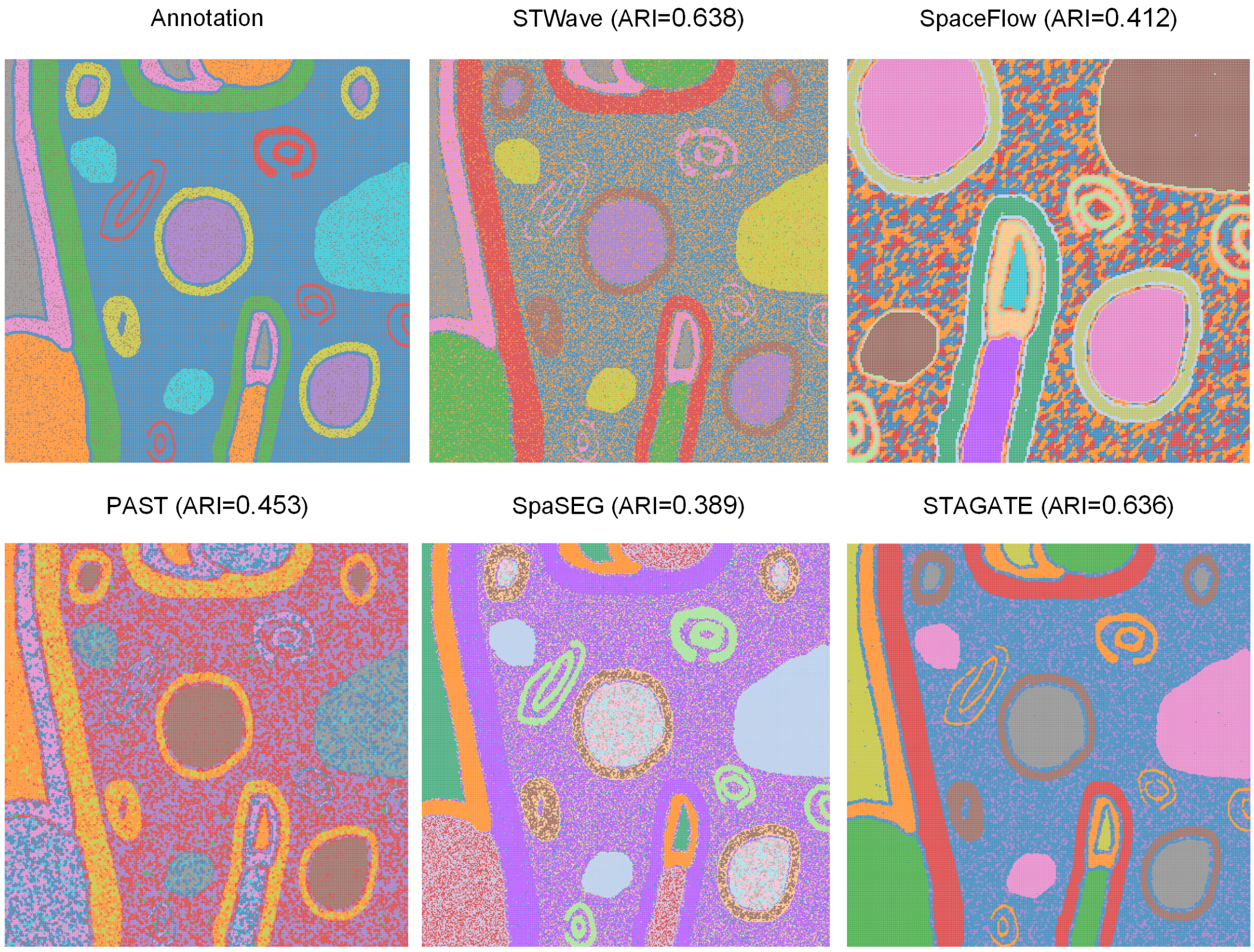

Figure S. 4 Spatial clustering results of STWave and comparator methods are presented on the simulated dataset with $160,000$ spots.

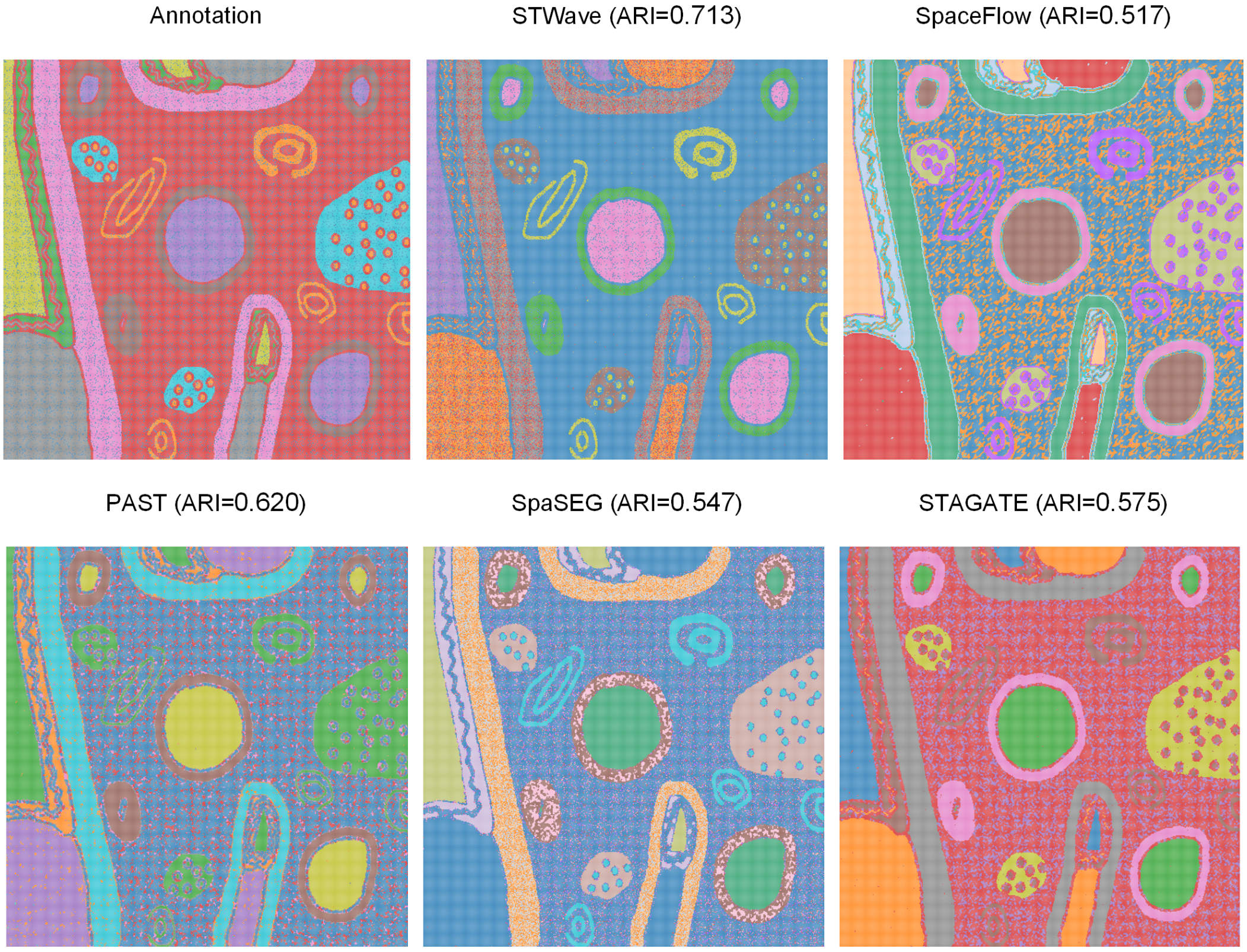

Figure S. 5 Spatial clustering results of STWave and comparator methods are presented on the simulated dataset with 240, 000 spots.

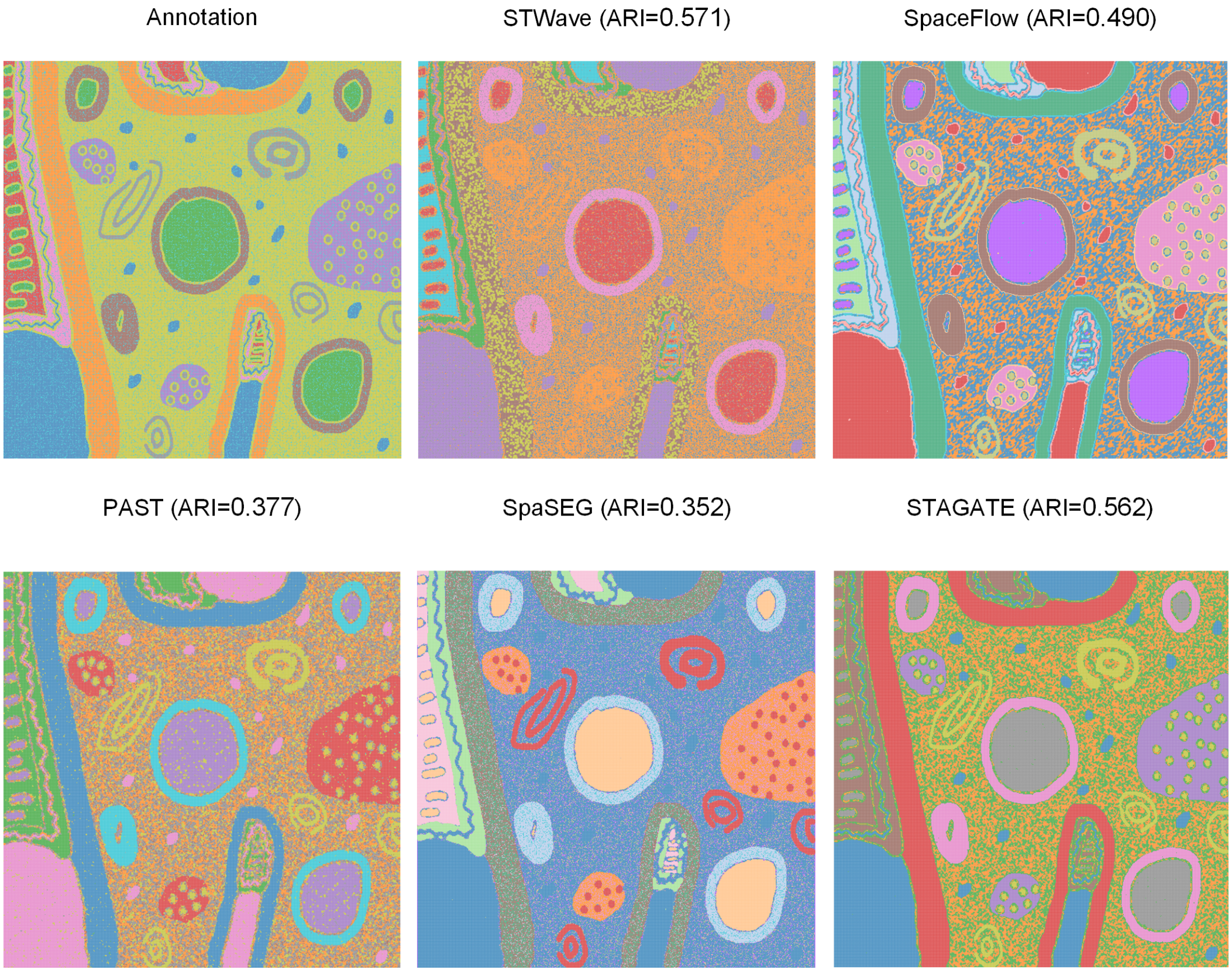

Figure S. 6 Spatial clustering results of STWave and comparator methods are presented on the simulated dataset with 320, 000 spots.

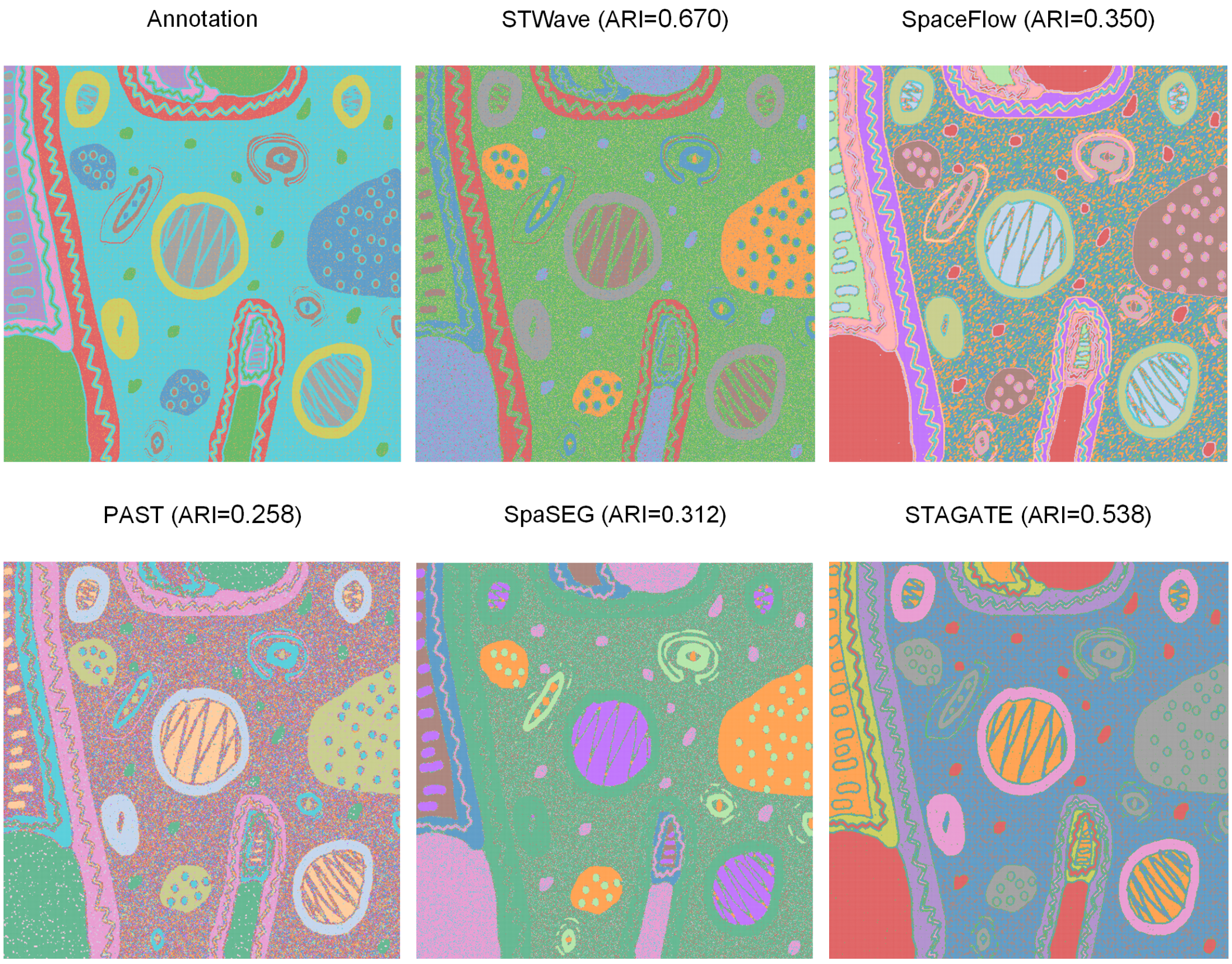

Figure S. 7 Spatial clustering results of STWave and comparator methods are presented on the simulated dataset with $480,000$ spots.

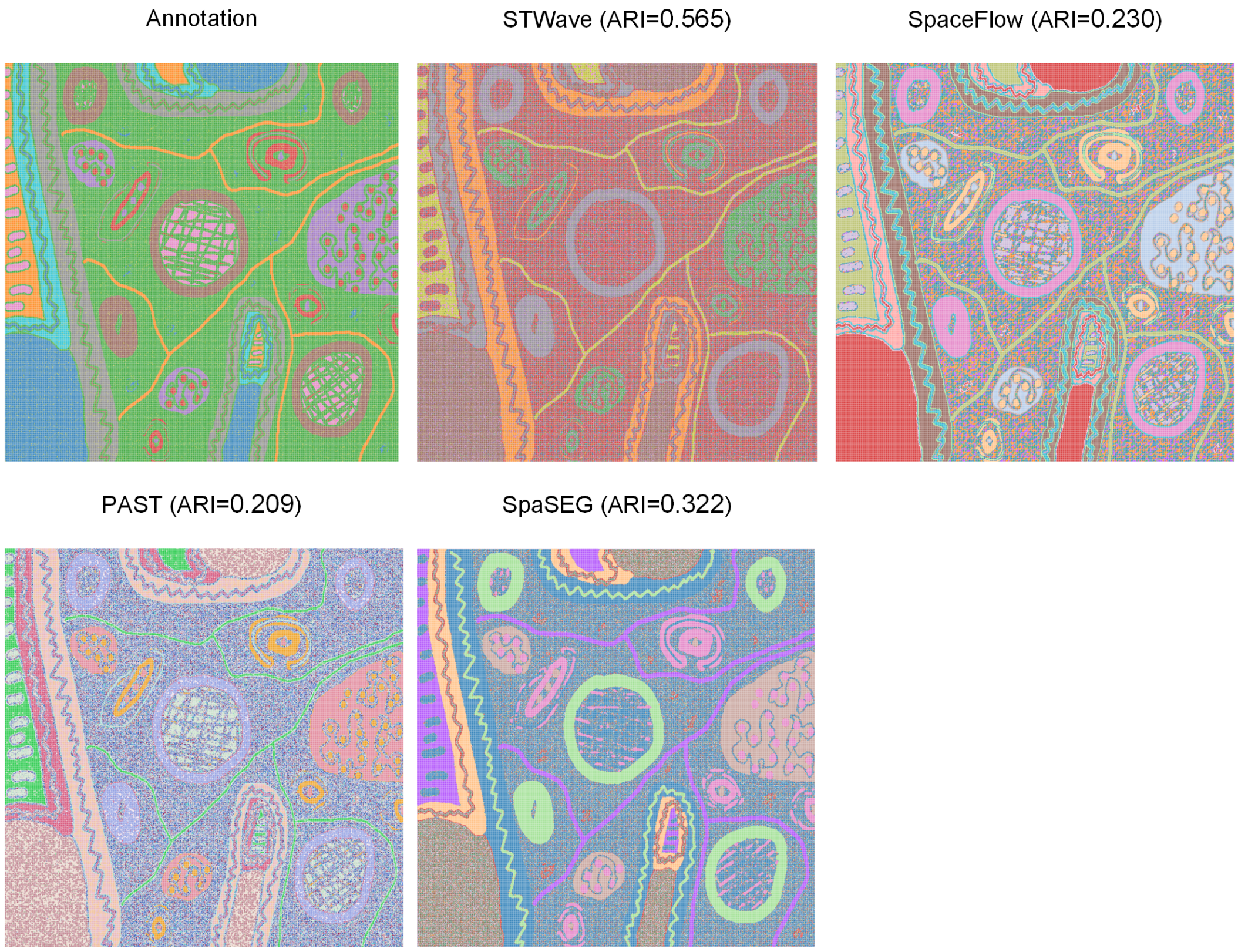

Figure S. 8 Spatial clustering results of STWave and comparator methods are presented on the simulated dataset with $640,000$ spots.

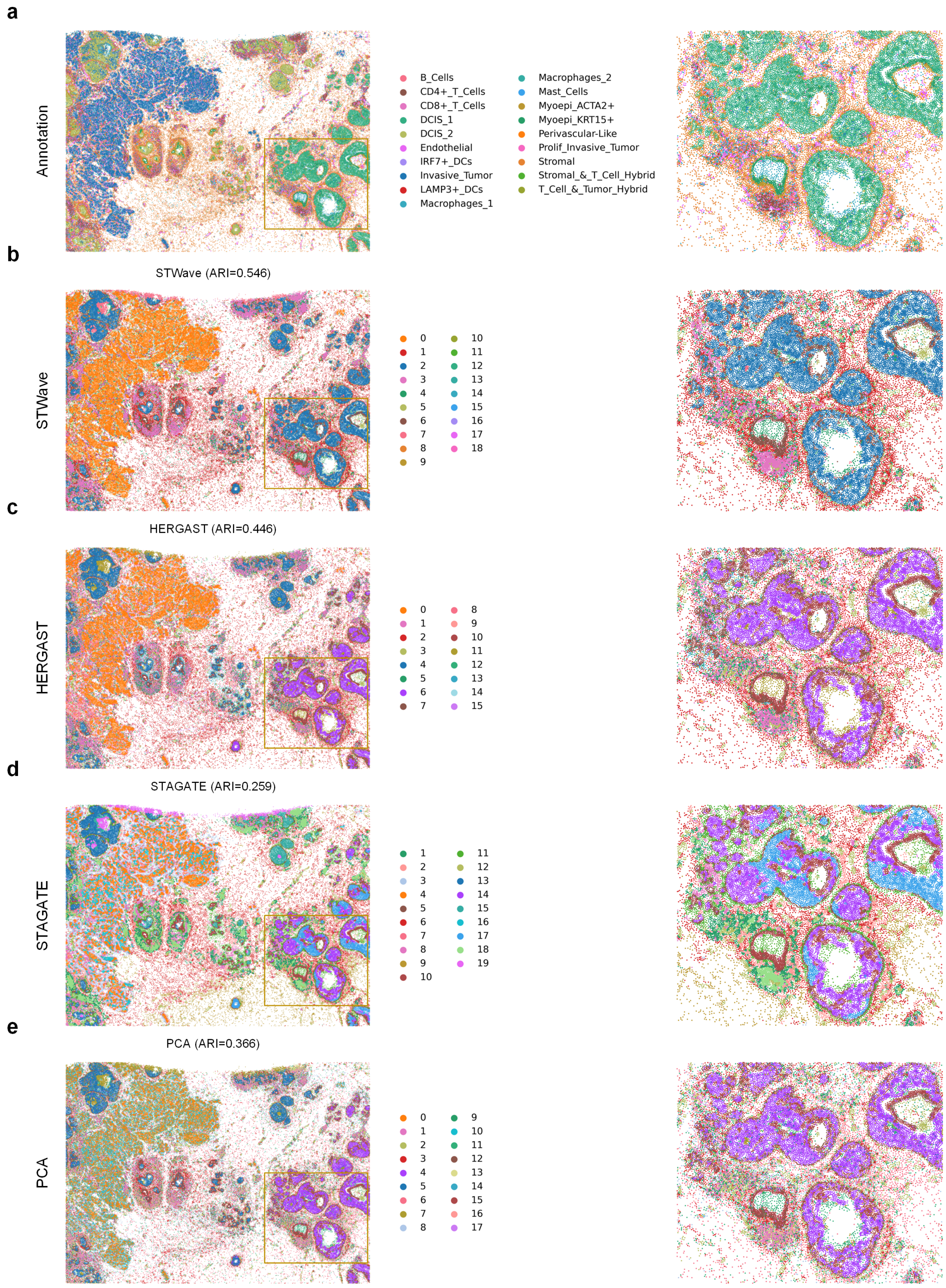

Figure S. 9 Spatial clustering visualizations on the Xenium human breast cancer dataset. a. Visualization of reference spatial annotations (left) and a magnified view of the region indicated by the red bounding box is shown (right). **b.** Visualization of the STWave spatial clustering result (left). A magnified view of the region indicated by the red bounding box is shown (right). **c.** Visualization of the HERGAST spatial clustering result (left). A magnified view of the region indicated by the red bounding box is shown (right). **d.** Visualization of the STAGATE spatial clustering result (left). A magnified view of the region indicated by the red bounding box is shown (right). **e.** Visualization of the PCA clustering result (left). A magnified view of the region indicated by the red bounding box is shown (right).

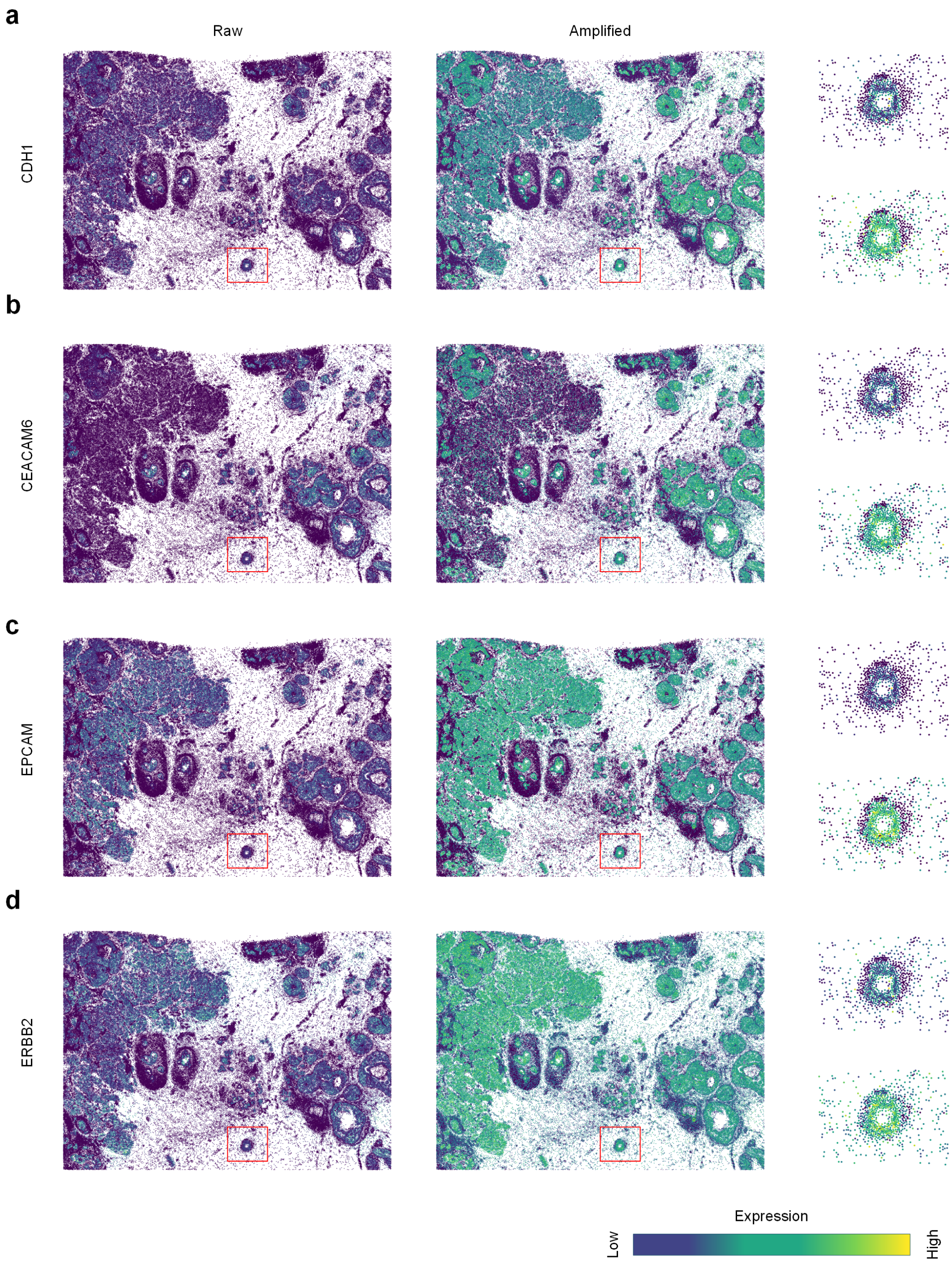

Figure S. 10 Spatial expression maps on the Xenium human breast cancer dataset. Shown for CDH1 (**a**), CEACAM6 (**b**), EPCAM (**c**), and ERBB2 (**d**) are the raw signal and the signal amplified by STWave. The final column presents the method specific original and amplified spatial expression.

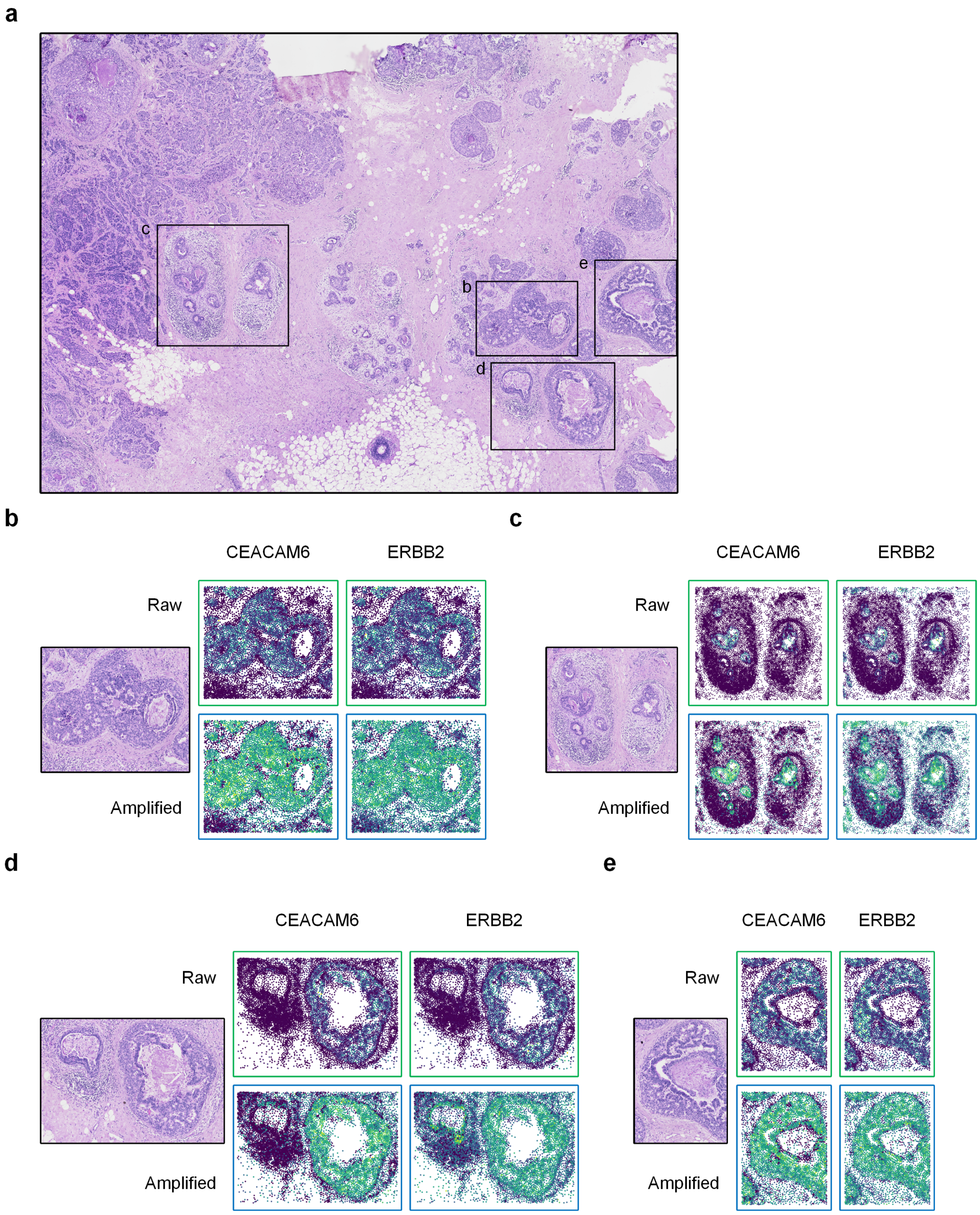

Figure S. 11 Spatial expression in the selected region is shown for the raw signal and for the signal amplified by STWave on the Xenium human breast cancer dataset. a. H&E stained image from the Xenium human breast cancer dataset. **b.** Left: H&E stained image of the region indicated by the bounding box labeled b in Figure 11a. Right: spatial expression maps of CEACAM6 and ERBB2 for the same region, showing the original signals and the signals amplified by STWave, consistent with the histology. **c.** Left: H&E stained image of the region indicated by the bounding box labeled c in Figure 11a. Right: spatial expression maps of CEACAM6 and ERBB2 for the same region, showing the original signals and the signals amplified by STWave, consistent with the histology. **d.** Left: H&E stained image of the region indicated by the bounding box labeled d in Figure 11a. Right: spatial expression maps of CEACAM6 and ERBB2 for the same region, showing the original signals and the signals amplified by STWave, consistent with the histology. **e.** Left: H&E stained image of the region indicated by the bounding box labeled e in Figure 11a. Right: spatial expression maps of CEACAM6 and ERBB2 for the same region, showing the original signals and the signals amplified by STWave, consistent with the histology.

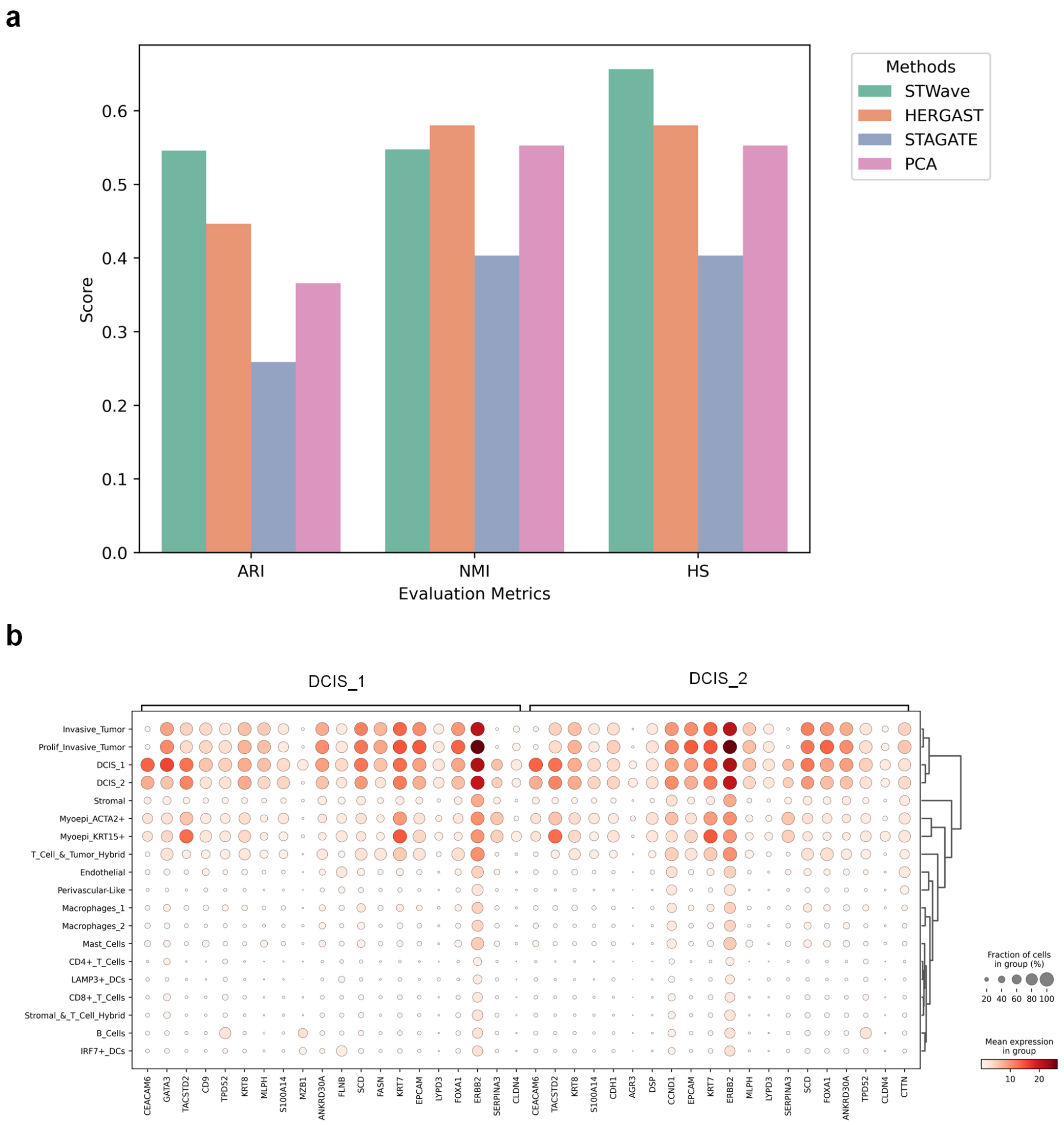

Figure S. 12 Performance of STWave and comparator methods was evaluated, and genes with high expression in two specific spatial domains were visualized on the Xenium human breast cancer dataset. **a.** Bar charts of Adjusted Rand Index, Normalized Mutual Information, and Homogeneity Score for STWave and comparator methods on the Xenium human breast cancer dataset. **b.** Dot plots of genes with high expression in the regions annotated as DCIS_1 and DCIS_2 on the Xenium human breast cancer dataset.

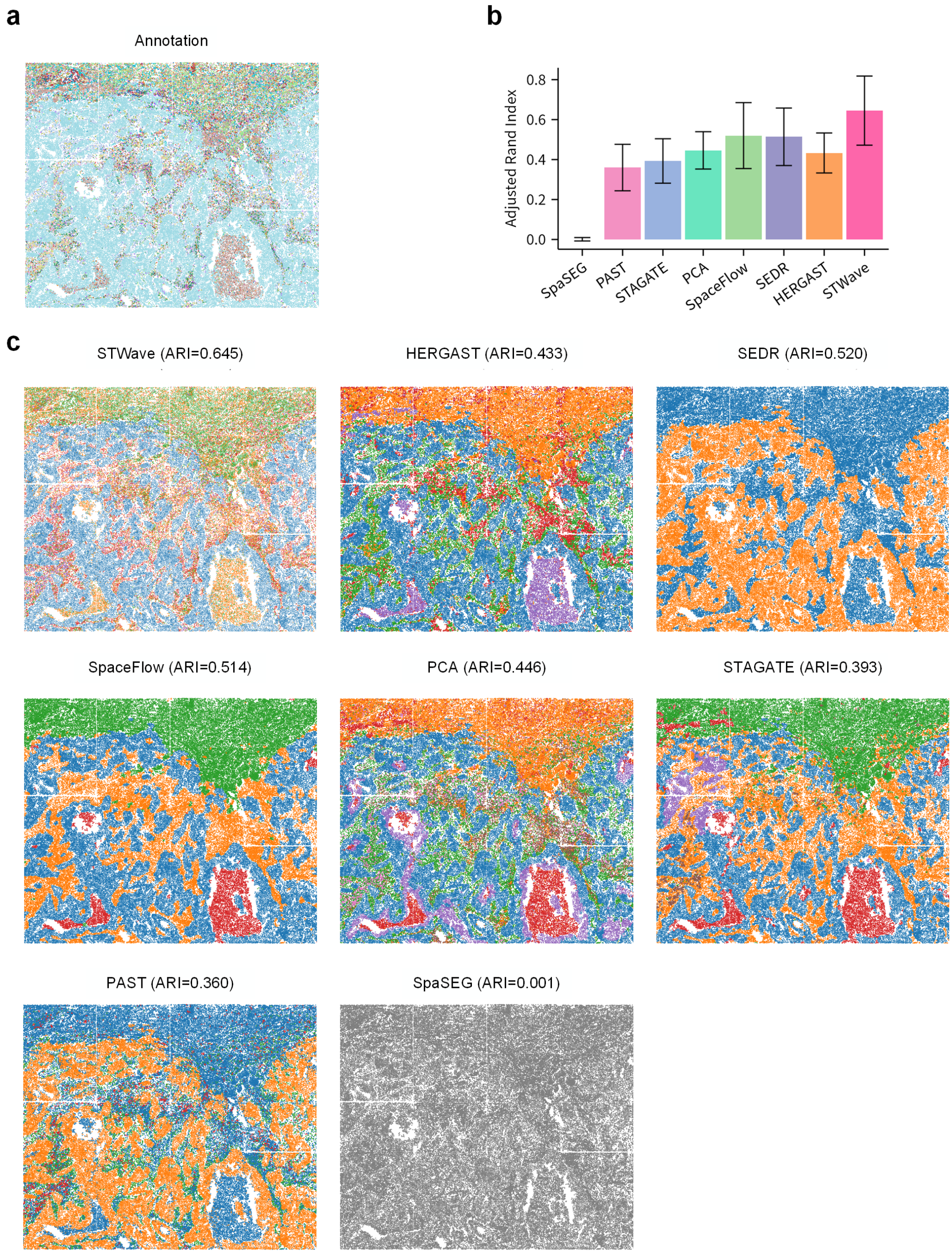

Figure S. 13 Spatial clustering visualizations of STWave and comparator methods were generated on the CosMx SMI human lung cancer dataset. a. Visualization of reference annotations on the CosMx SMI human lung cancer dataset. **b.** Bar charts of Adjusted Rand Index for different methods on the CosMx SMI human lung cancer dataset; the black line indicates the standard deviation across resolution settings. **c.** Spatial clustering visualizations produced by STWave and comparator methods with the Adjusted Rand Index reported on the CosMx SMI human lung cancer dataset.

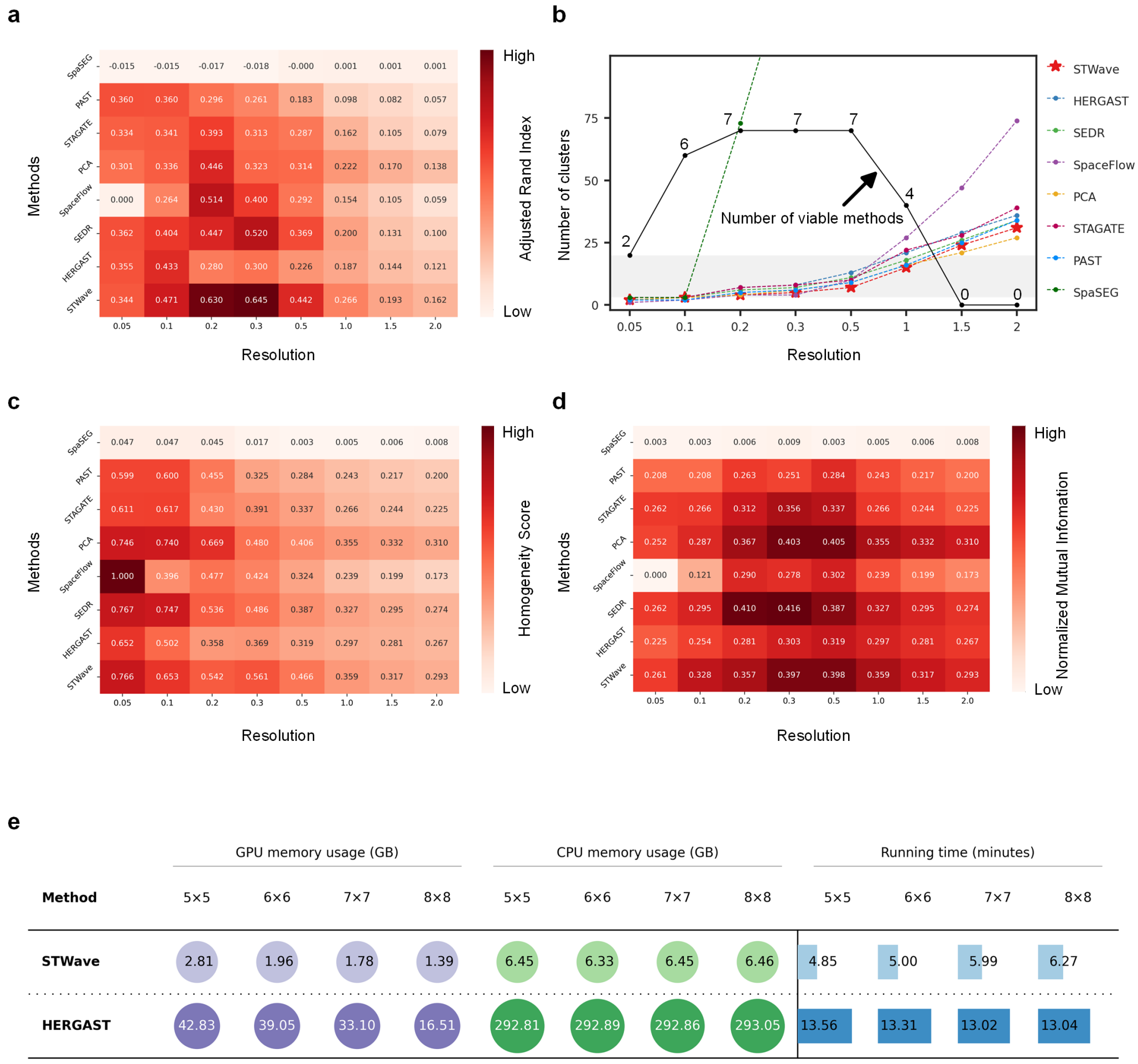

Figure S. 14 Performance of STWave was evaluated on the CosMx SMI human lung cancer dataset. **a.** Adjusted Rand Index of different methods under Leiden clustering with varying resolution settings on the CosMx SMI human lung cancer dataset. **b.** Number of clusters produced by different methods under Leiden clustering with varying resolution settings on the CosMx SMI human lung cancer dataset; gray rectangles indicate methods that are available when the cluster count lies between 3 and 20; the black solid line reports the number of available methods. **c.** Normalized Mutual Information of different methods under Leiden clustering with varying resolution settings on the CosMx SMI human lung cancer dataset. **d.** Homogeneity Score of different methods under Leiden clustering with varying resolution settings on the CosMx SMI human lung cancer dataset. **e.** GPU usage, CPU usage, and runtime for STWave and HERGAST when the tissue is divided into 5×5, 6×6,7×7, and 8×8 patches on the CosMx SMI human lung cancer dataset.

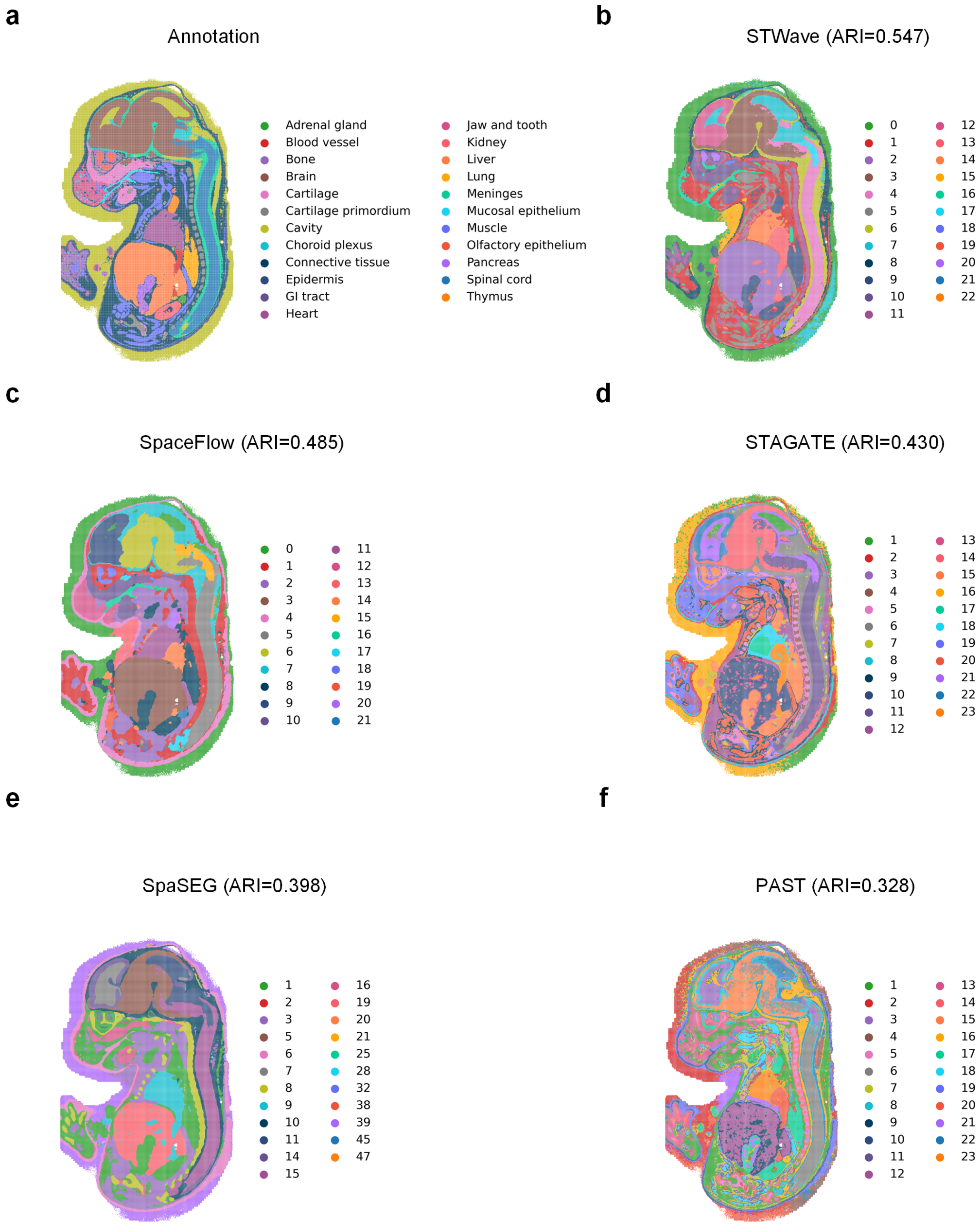

Figure S. 15 Spatial clustering visualizations of STWave and comparator methods were generated on the mouse embryo E16.5 dataset. a. Reference spatial annotations on the mouse embryo E16.5 dataset. **b–f.** Spatial clustering results of STWave, SpaceFlow, STAGATE, SpaSEG, and PAST, with the corresponding Adjusted Rand Index reported.

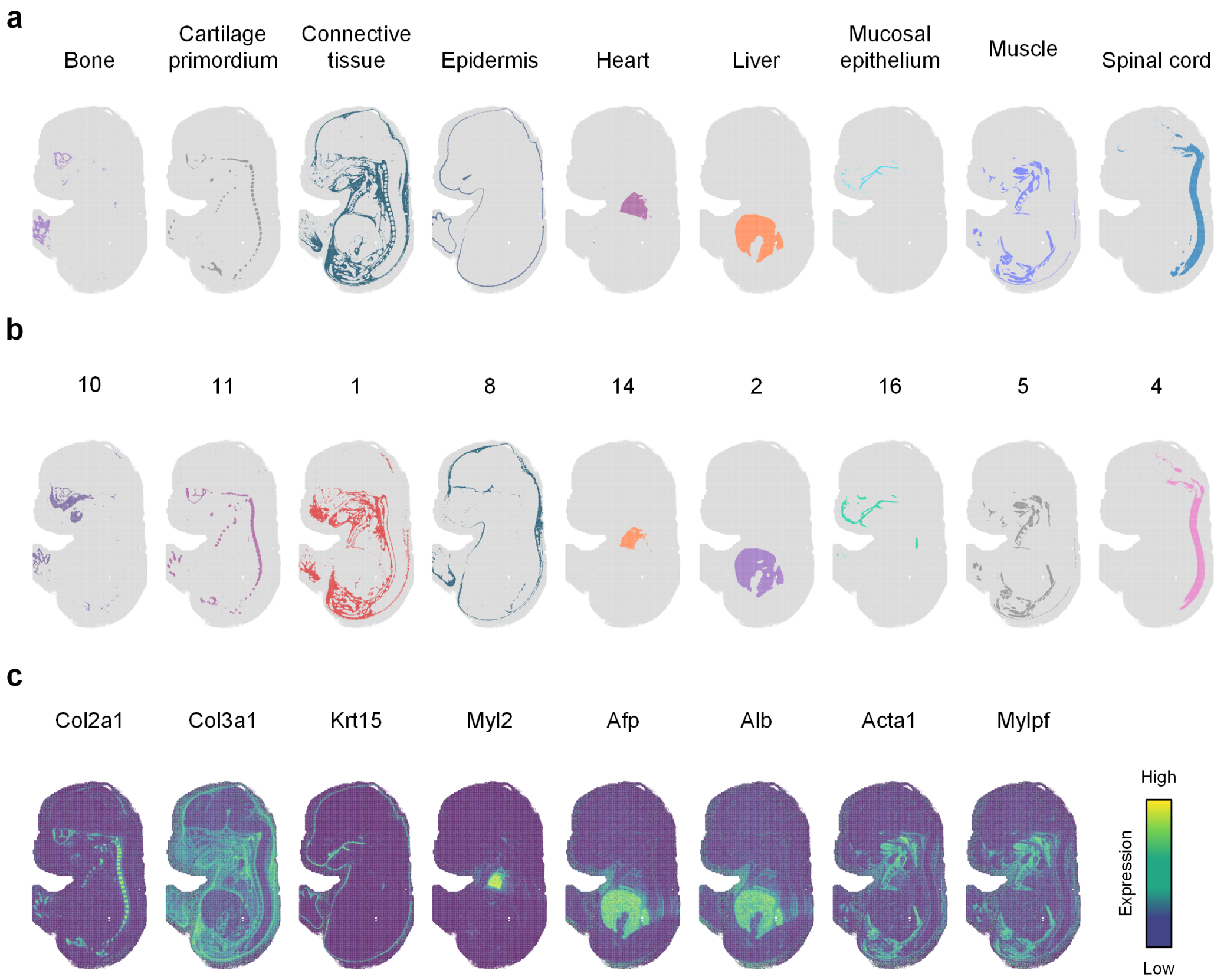

Figure S. 16 Selected spatial domains identified by STWave and the corresponding reference annotations are displayed separately. a. Visualization of reference annotations on the mouse embryo E16.5 dataset. **b.** Spatial domains identified by STWave that are consistent with the annotations in Figure 16a. **c.** Spatial expression patterns of representative marker genes that are consistent with the annotations in Figure 16a.

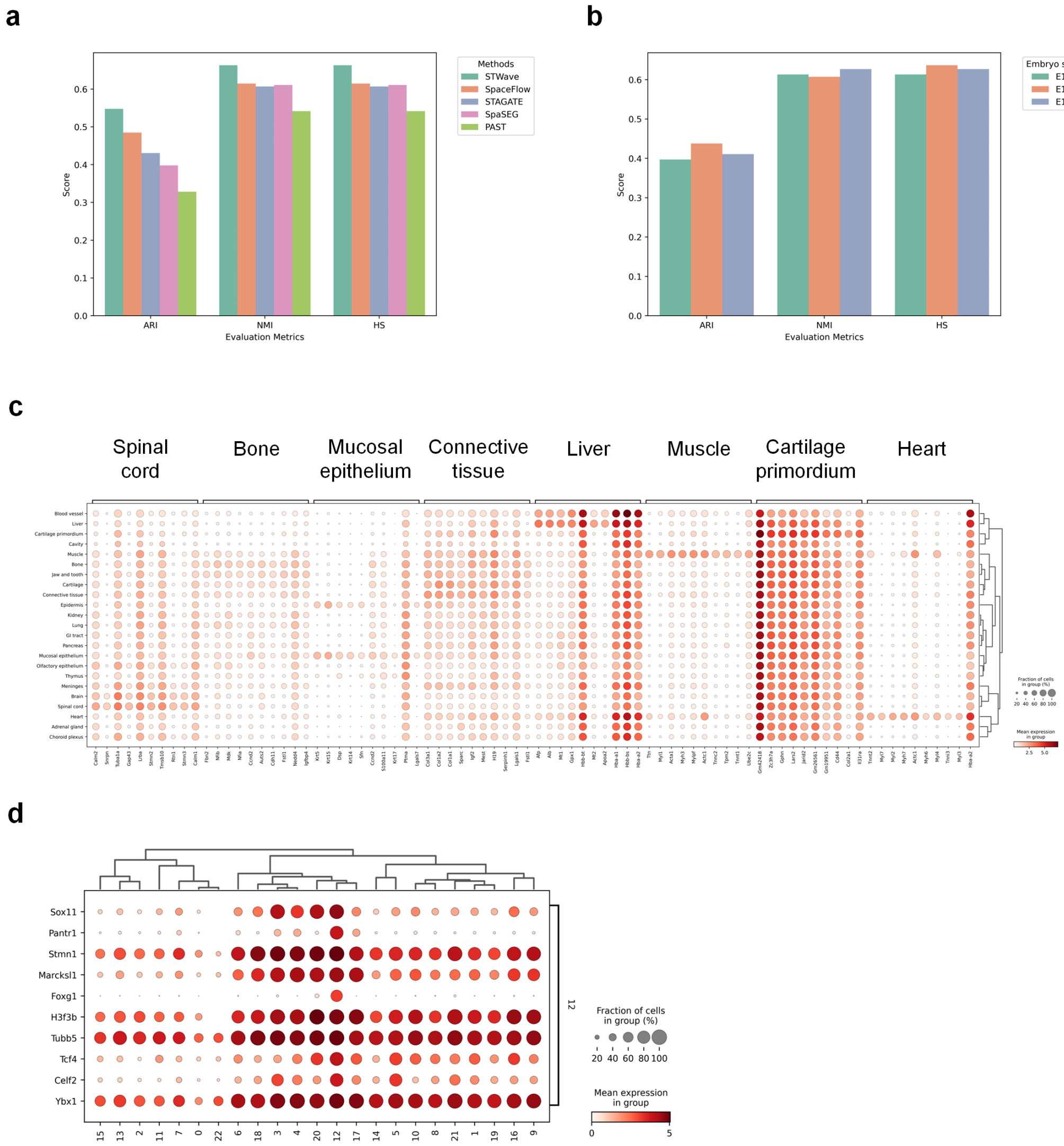

Figure S. 17 Performance of STWave and comparator methods was evaluated, and genes with high expression in several specific spatial domains were visualized on the mouse embryo E16.5 dataset. a. Bar plots of Adjusted Rand Index, Normalized Mutual Information, and Homogeneity Score for STWave and comparator methods on the mouse embryo E16.5 dataset. **b.** Bar plots of Adjusted Rand Index, Normalized Mutual Information, and Homogeneity Score for STWave on the mouse embryo E13.5, E14.5, and E15.5 datasets. **c.** Dot plot of genes with high expression in the spatial domain identified by STWave. **d.** Dot plot of genes with high expression in the dorsal forebrain domain identified by STWave.

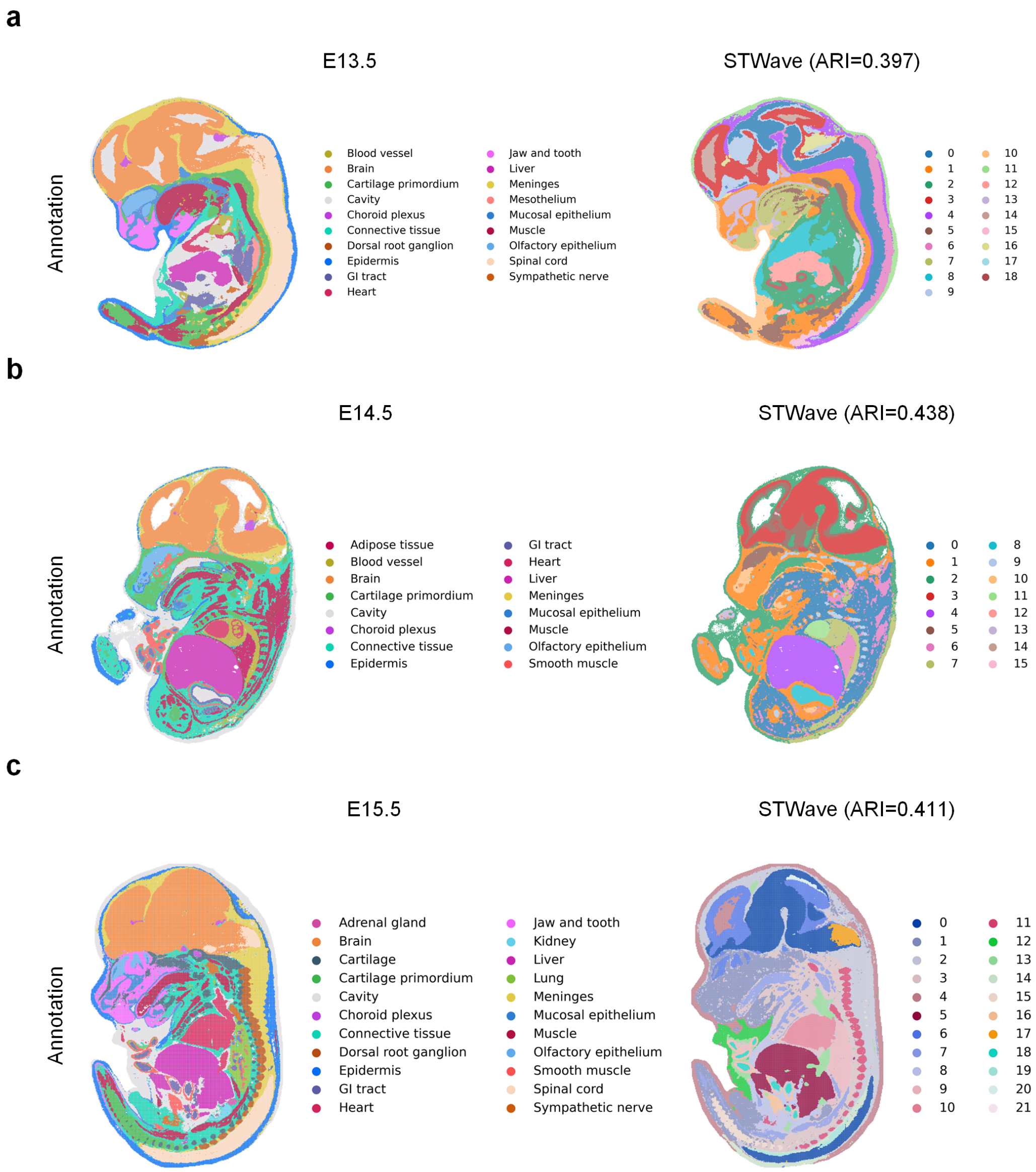

Figure S. 18 Spatial clustering visualizations produced by STWave, together with reference annotation visualizations, were generated on three mouse embryo datasets. a. Spatial clustering visualizations produced by STWave, together with reference annotation visualizations, were generated on the mouse embryo E13.5 dataset. **b.** Spatial clustering visualizations produced by STWave, together with reference annotation visualizations, were generated on the mouse embryo E14.5 dataset. **c.** Spatial clustering visualizations produced by STWave, together with reference annotation visualizations, were generated on the mouse embryo E15.5 dataset.

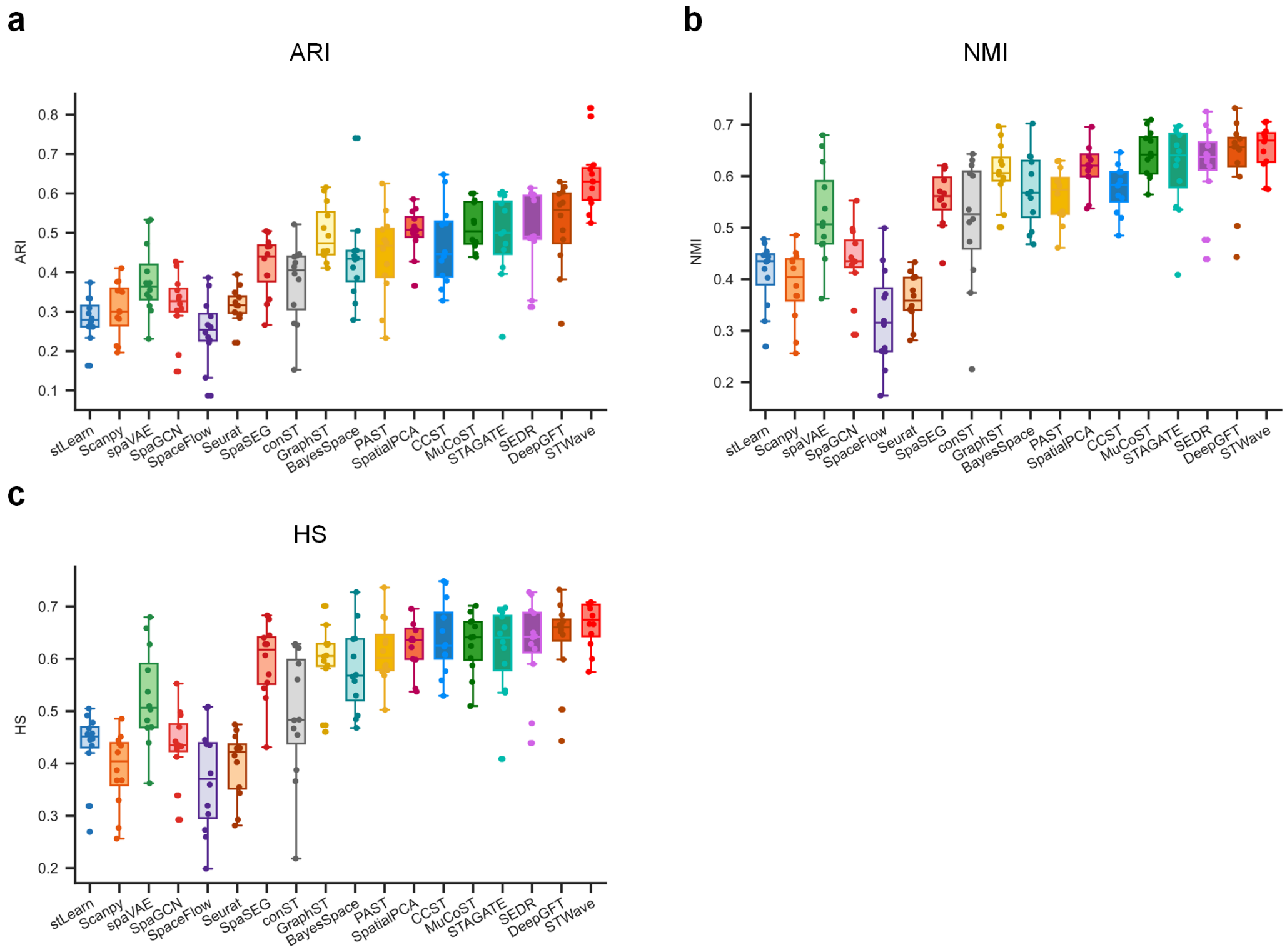

Figure S. 19 Clustering accuracy metrics on the 10x Visium DLPFC dataset. a. Box plots of Adjusted Rand Index. **b.** Box plots of Normalized Mutual Information. **c.** Box plots of Homogeneity Score for STWave and comparator methods across twelve human DLPFC slices.

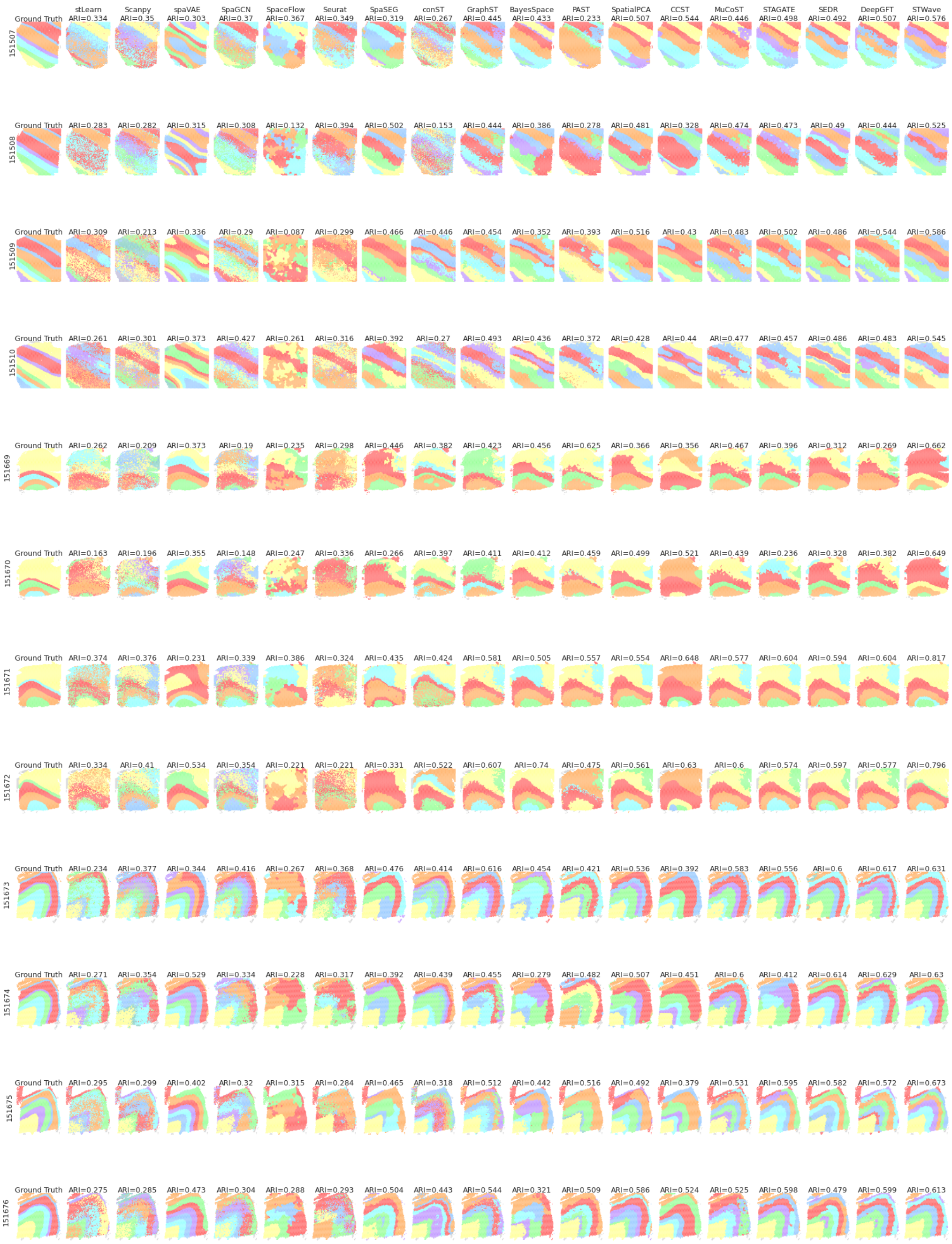

Figure S. 20 Spatial clustering visualizations on the human DLPFC dataset were generated for STWave and other methods.

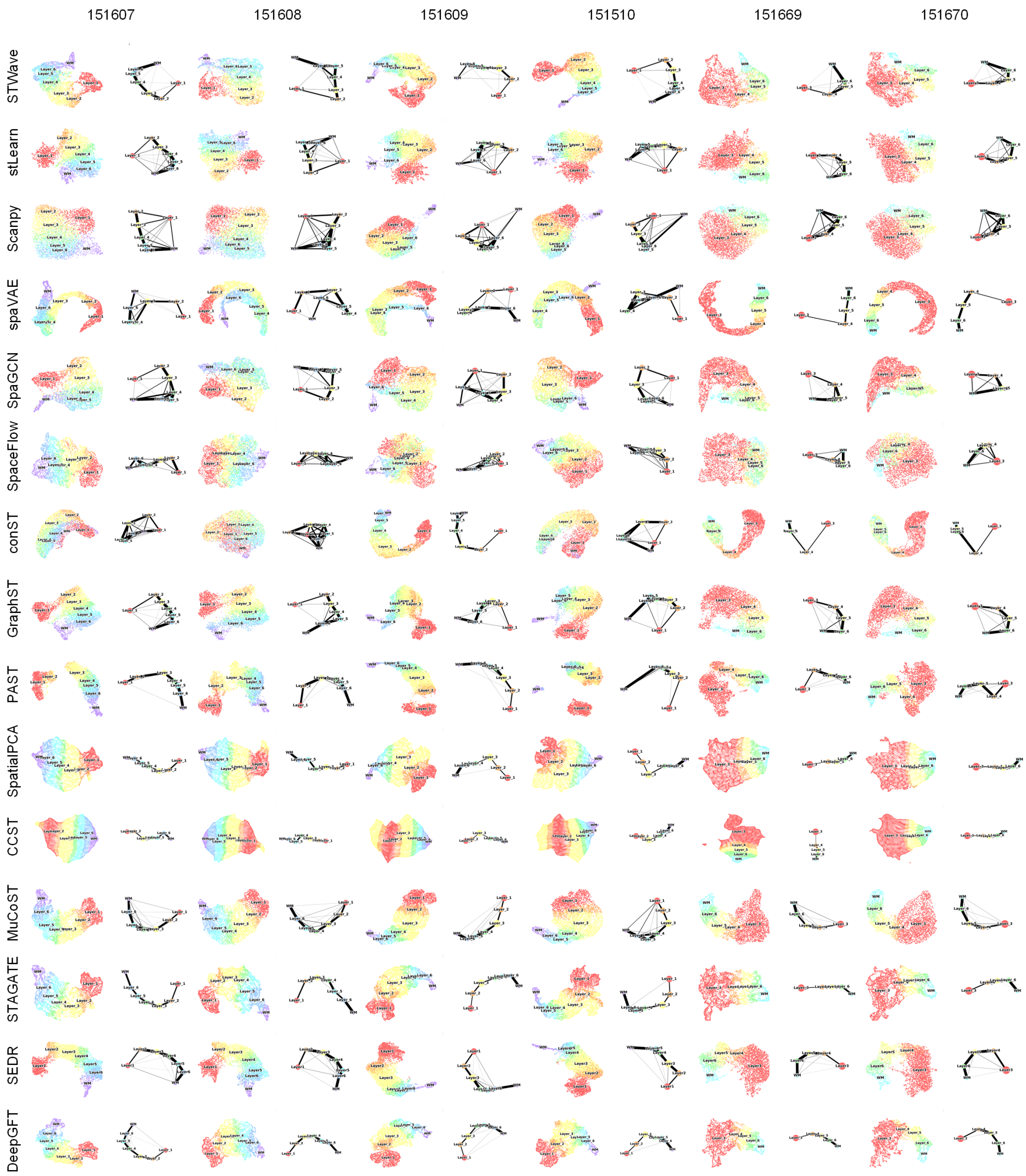

Figure S. 21 PAGA graphs and UMAP visualizations on the first six human DLPFC slices were generated for STWave and other methods.

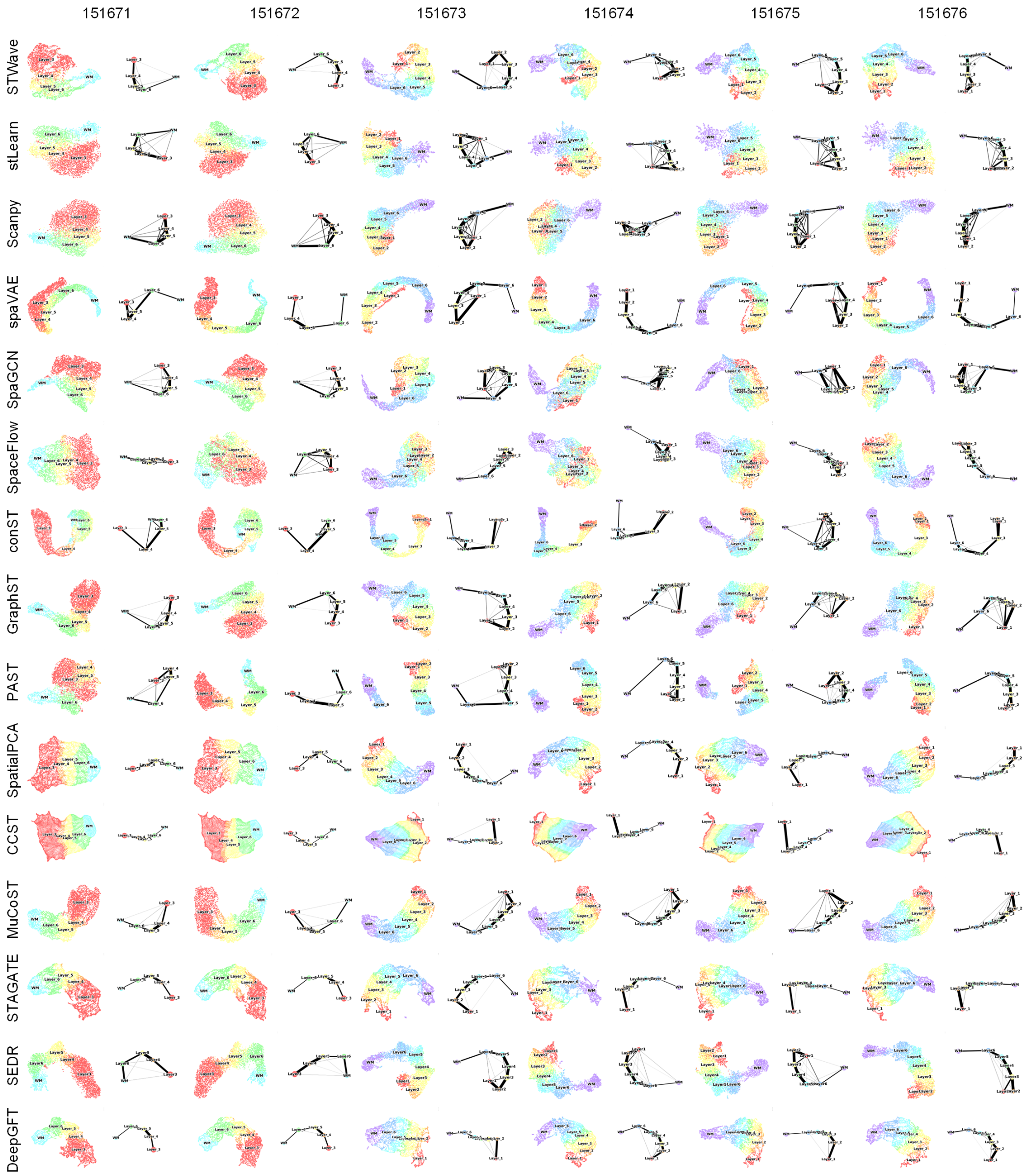

Figure S. 22 PAGA graphs and UMAP visualizations on the last six human DLPFC slices were generated for STWave and other methods.

a

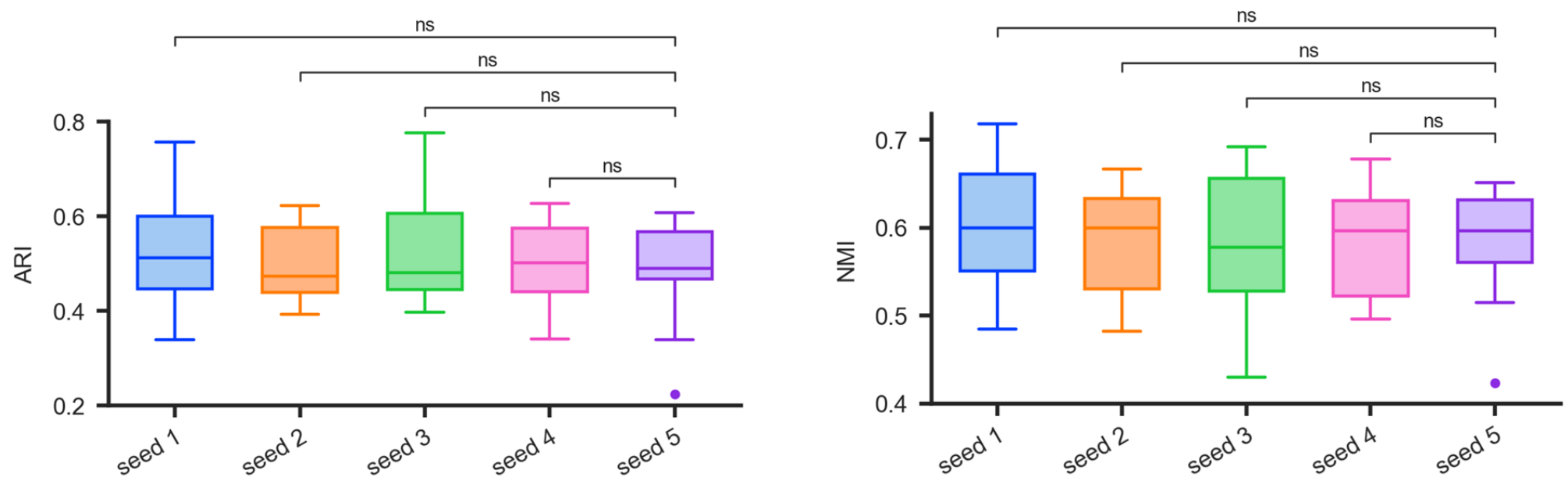

Figure S. 23 Distributions of ARI and NMI on the DLPFC dataset under different randomized gene orders. a. Boxplots show the distributions of ARI and NMI on the DLPFC dataset across five randomized gene orders. Each box summarizes the ARI or NMI values across 12 DLPFC slices for one randomized gene order generated using the indicated random seed, and "ns" indicates no statistically significant difference between groups.

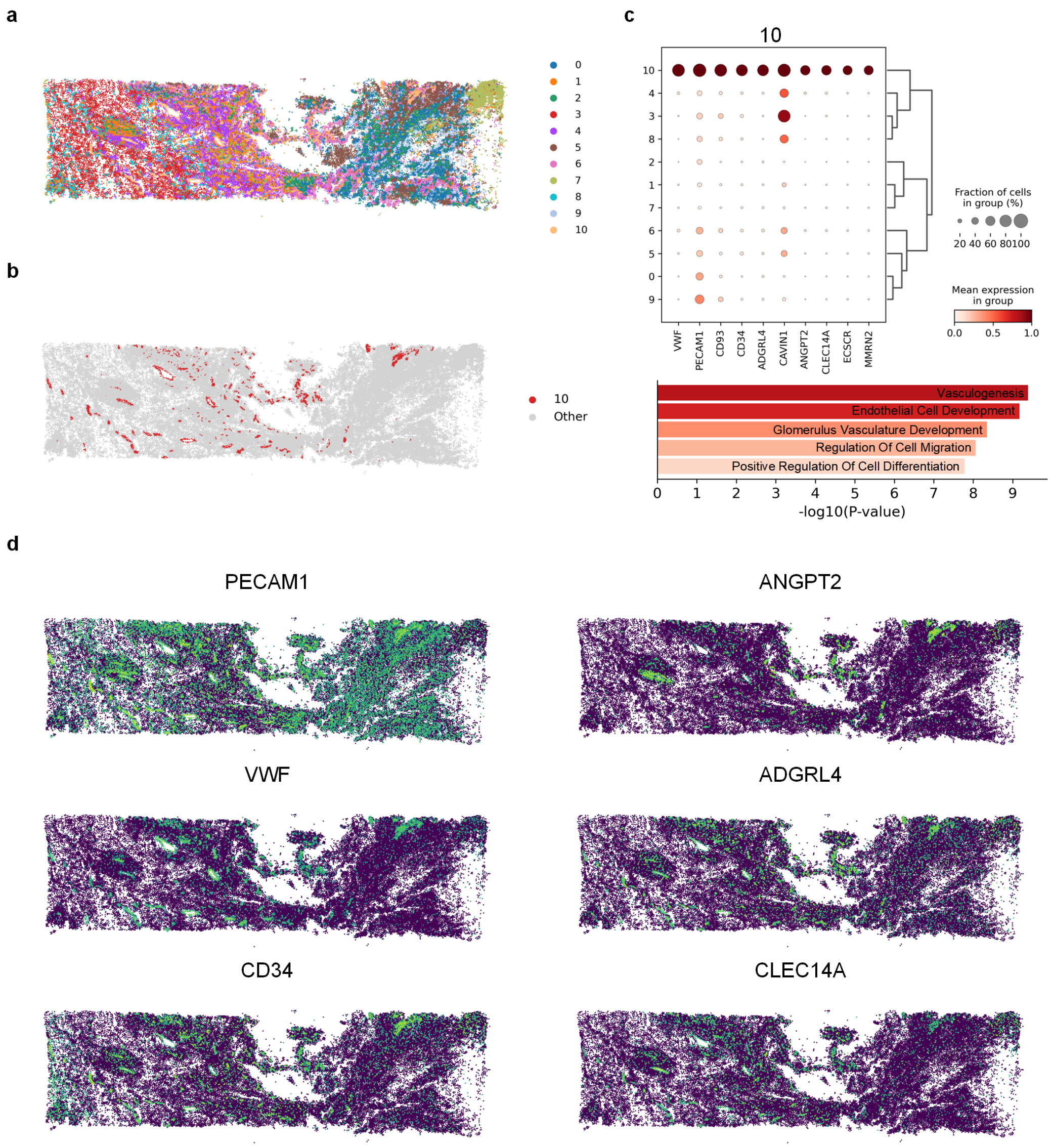

Figure S. 24 Spatial visualization and spatial expression analysis on the Xenium human kidney cancer dataset. a. Spatial visualization of the domains identified from the Xenium human kidney cancer tissue section. Each color denotes one spatial domain. **b.** Spatial visualization of Domain 10. Spots assigned to Domain 10 are highlighted in red, while spots from the remaining domains are shown in gray. **c.** Marker gene expression and Gene Ontology enrichment results for Domain 10. The dot plot shows the expression of selected marker genes across all domains. Dot size indicates the fraction of spots expressing the gene, and color intensity indicates the mean expression level. The bar plot shows the top enriched Gene Ontology biological process terms for Domain 10 marker genes, ranked by $-\log_{10}(P\text{-value})$. **d.** Spatial expression analysis of selected vascular and endothelial marker genes, including PECAM1, VWF, CD34, ANGPT2, ADGRL4, and CLEC14A.

a

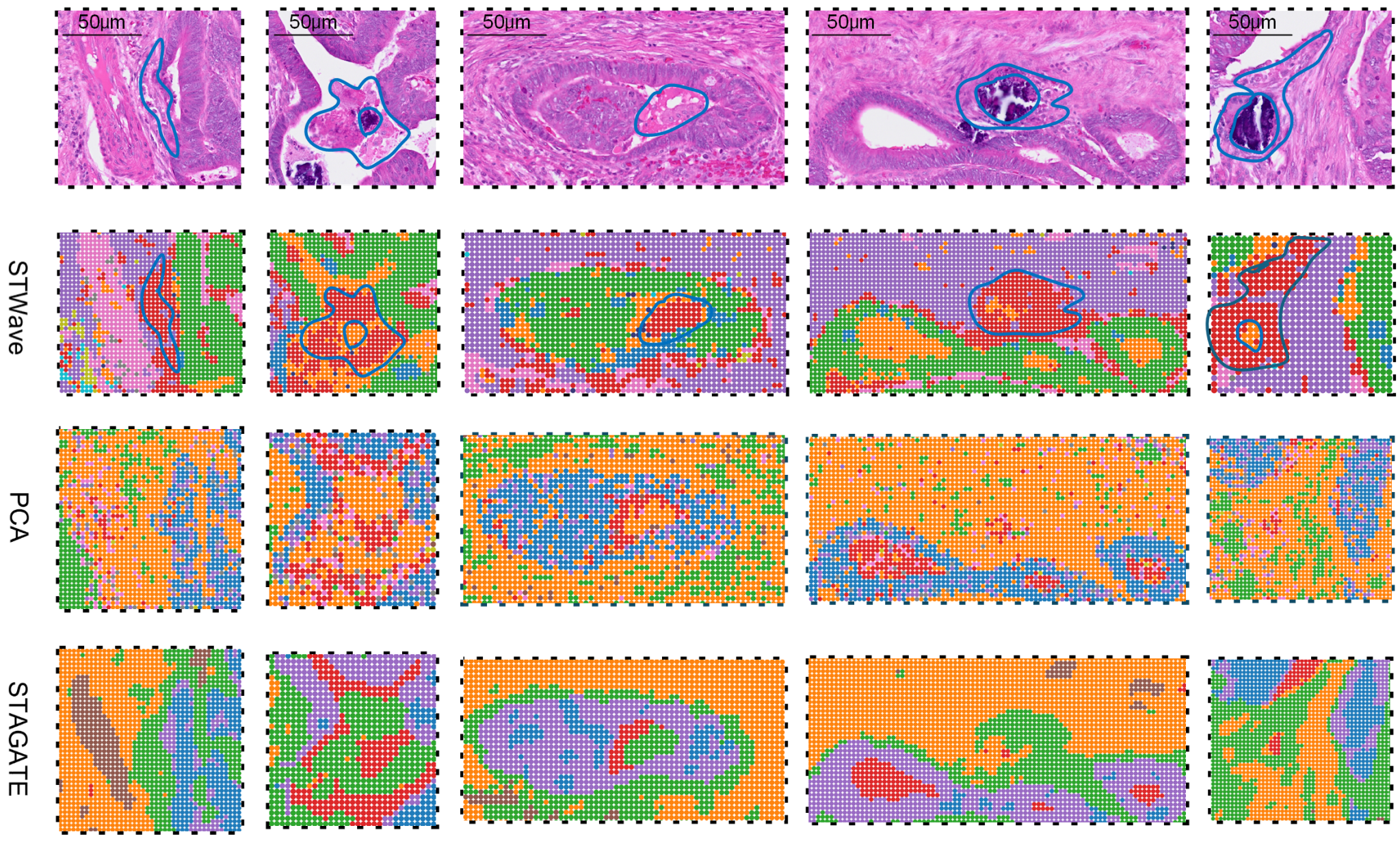

Figure S. 25 Microenvironmental comparison of calcified regions in the Visium HD human colorectal cancer dataset. a. H&E-stained images and the corresponding spatial clustering visualizations generated by STWave, PCA, and STAGATE are shown for the selected colorectal cancer regions. Blue outlines mark the calcified regions in the H&E images and the corresponding spatial domains identified by STWave, enabling direct comparison with the matched regions in PCA and STAGATE.

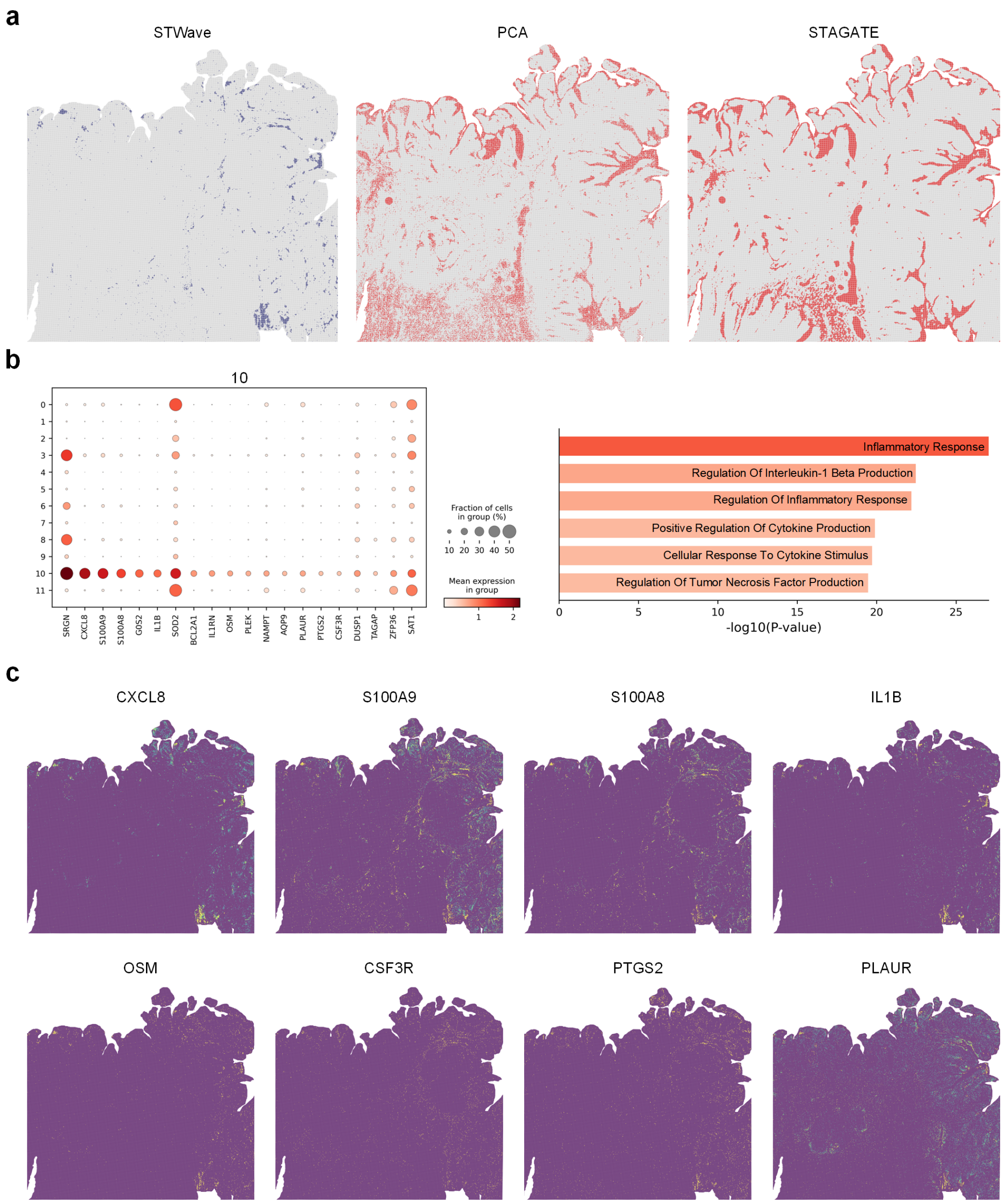

Figure S. 26 Spatial and functional characterization of the $CXCL8$-high inflammatory region in the Visium HD human colorectal cancer dataset. a. Spatial visualization of STWave Domain 10 and the spatially matched domains identified by PCA and STAGATE. **b.** Marker gene expression and Gene Ontology enrichment analysis for STWave Domain 10. The dot plot shows the expression of selected marker genes across domains. The bar plot shows enriched Gene Ontology biological process terms for Domain 10 marker genes, ranked by $-\log_{10}(P\text{-value})$. **c.** Spatial expression maps of representative genes highly expressed in STWave Domain 10, including $CXCL8$, $S100A9$, $S100A8$, $IL1B$, OSM, $CSF3R$, $PTGS2$, and $PLAUR$.

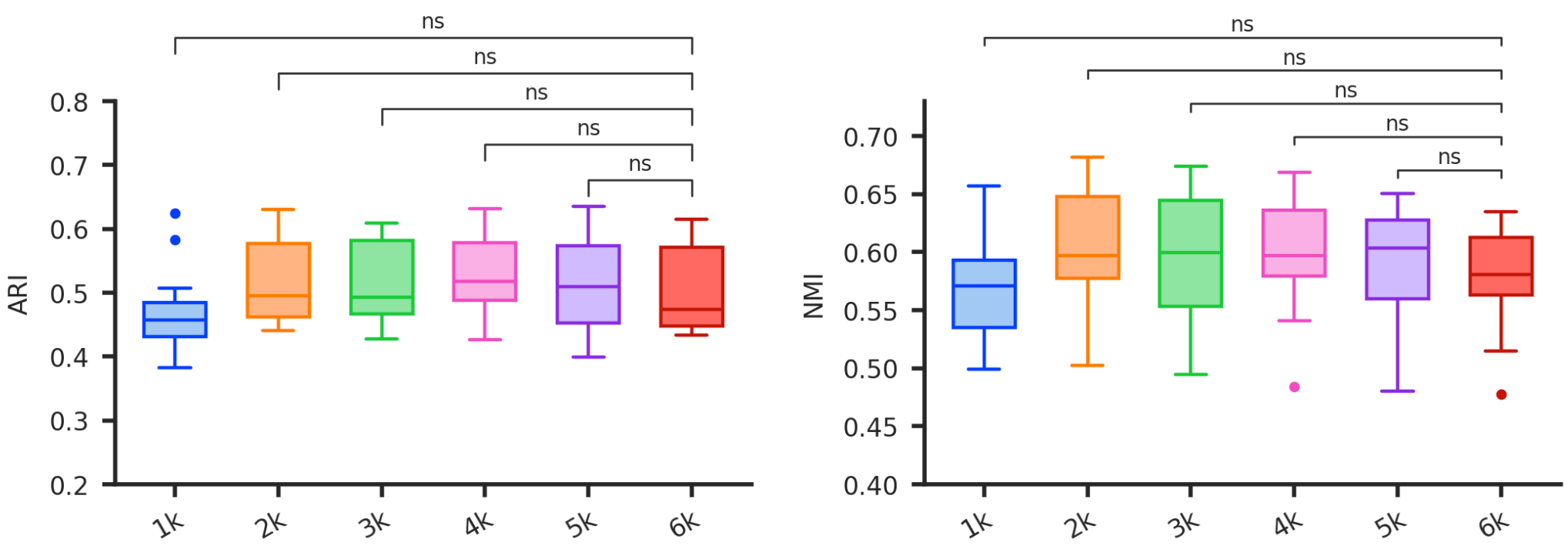

Figure S. 27 Sensitivity of STWave to the number of highly variable genes on the DLPFC dataset. Box plots show the Adjusted Rand Index and Normalized Mutual Information for STWave on the DLPFC dataset when using 1,000, 2,000, 3,000, 4,000, 5,000, and 6,000 highly variable genes. Each box summarizes the results across 12 DLPFC slices for the indicated number of highly variable genes. "ns" indicates no statistically significant difference between the corresponding settings.

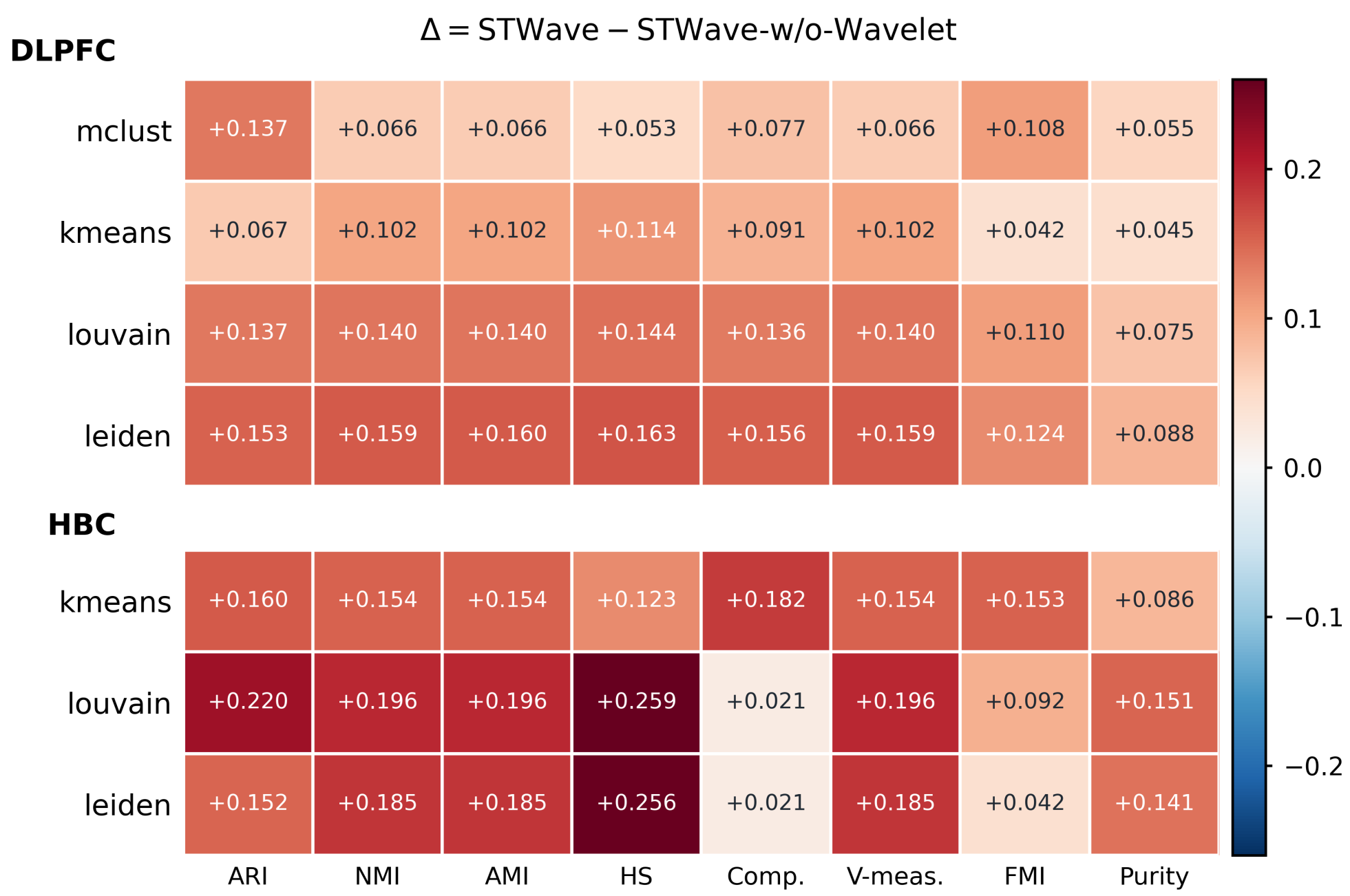

Figure S. 28 Difference heatmap between STWave and STWave without the wavelet transform on the DLPFC and Xenium human breast cancer datasets. The heatmap reports the average difference between STWave and STWave without the wavelet transform across multiple clustering metrics, where positive values indicate better performance of STWave.

Method	Clustering metrics							
	ARI	NMI	AMI	HS	Comp.	V-meas.	FMI	Purity
**STWave**	.64±.09	.65±.04	.70±.05	.70±.05	.71±.05	.70±.05	.73±.08	.81±.05
**stLearn**	.28±.05	.41±.06	.43±.06	.43±.06	.43±.07	.43±.06	.45±.05	.64±.07
**Scanpy**	.30±.07	.39±.07	.42±.06	.46±.05	.39±.07	.42±.06	.45±.05	.67±.07
**spaVAE**	.38±.09	.53±.09	.55±.10	.58±.11	.53±.09	.55±.10	.52±.07	.71±.10
**SpaGCN**	.32±.08	.44±.07	.47±.06	.51±.06	.44±.07	.47±.06	.46±.05	.68±.09
**SpaceFlow**	.25±.09	.33±.10	.35±.09	.34±.10	.37±.10	.35±.09	.44±.07	.58±.09
**Seurat**	.32±.04	.36±.05	.37±.05	.36±.04	.39±.07	.37±.05	.48±.05	.60±.07
**SpaSEG**	.42±.08	.56±.06	.58±.05	.58±.05	.59±.07	.58±.05	.56±.05	.71±.06
**conST**	.37±.10	.51±.13	.51±.13	.54±.13	.49±.12	.51±.13	.51±.10	.66±.09
**GraphST**	.50±.07	.61±.06	.61±.06	.62±.05	.60±.07	.61±.06	.62±.06	.76±.07
**BayesSpace**	.43±.12	.57±.07	.59±.07	.61±.07	.58±.08	.60±.07	.56±.11	.72±.10
**PAST**	.44±.11	.56±.05	.59±.05	.57±.06	.61±.06	.59±.05	.59±.10	.71±.08
**SpatialPCA**	.50±.06	.62±.05	.64±.04	.65±.04	.63±.05	.64±.04	.62±.04	.76±.06
**CCST**	.47±.10	.57±.05	.61±.05	.58±.05	.64±.07	.61±.05	.61±.10	.70±.05
**MuCoST**	.52±.06	.64±.05	.64±.05	.66±.04	.63±.06	.64±.05	.63±.05	.78±.06
**STAGATE**	.49±.11	.62±.09	.64±.07	.66±.06	.62±.09	.64±.07	.61±.07	.77±.07
**SEDR**	.50±.10	.62±.08	.64±.07	.65±.05	.63±.09	.64±.07	.62±.06	.77±.06
**DeepGFT**	.52±.11	.63±.08	.65±.07	.67±.06	.64±.08	.65±.07	.63±.06	.78±.06

Figure S. 29 Comprehensive clustering metric comparison on the human DLPFC dataset. Multiple clustering metrics were summarized across competing methods on the DLPFC benchmark, with larger circles and darker colors indicating higher mean performance.

a

Region-Gene	Method	Evaluation subset: Detected cells	Regional gain: RSG	Cellwise concordance ↑: PCC	Cellwise concordance ↑: Spearman	Reconstruction error ↓: RMSE	Reconstruction error ↓: MAE	Reconstruction error ↓: NRMSE	Reconstruction error ↓: NMAE
**R1-EPCAM**	w/o Wavelet	562 (66.0%)	6.204	-0.680	-0.603	3.833	3.716	1.109	1.075
	STWave	562 (66.0%)	4.423	**0.403**	**0.414**	**2.612**	**2.476**	**0.755**	**0.716**
**R1-CDH1**	w/o Wavelet	609 (71.5%)	2.616	-0.665	-0.609	4.317	4.222	1.312	1.283
	STWave	609 (71.5%)	2.080	**0.247**	**0.259**	**3.368**	**3.260**	**1.024**	**0.991**
**R1-CEACAM6**	w/o Wavelet	685 (80.4%)	4.155	-0.631	-0.621	3.902	3.776	1.132	1.095
	STWave	685 (80.4%)	2.918	**0.417**	**0.422**	**3.715**	**3.629**	**1.077**	**1.052**
**R2-CDH1**	w/o Wavelet	4,372 (61.2%)	2.789	-0.016	0.001	4.183	4.137	1.539	1.522
	STWave	4,372 (61.2%)	2.425	**0.225**	**0.241**	**3.098**	**3.026**	**1.140**	**1.113**
**R2-ERBB2**	w/o Wavelet	6,650 (93.0%)	3.504	0.407	0.351	4.362	4.311	1.429	1.412
	STWave	6,650 (93.0%)	2.385	**0.465**	**0.451**	**4.232**	**4.172**	**1.387**	**1.367**
**R3-ERBB2**	w/o Wavelet	7,197 (85.4%)	4.036	**0.417**	0.445	**4.270**	**4.205**	**1.311**	**1.291**
	STWave	7,197 (85.4%)	2.457	0.415	**0.455**	4.339	4.265	1.332	1.309
**R3-CEACAM6**	w/o Wavelet	4,860 (57.7%)	3.019	-0.003	0.043	4.175	4.083	1.235	1.208
	STWave	4,860 (57.7%)	2.228	**0.512**	**0.546**	**3.930**	**3.858**	**1.163**	**1.141**

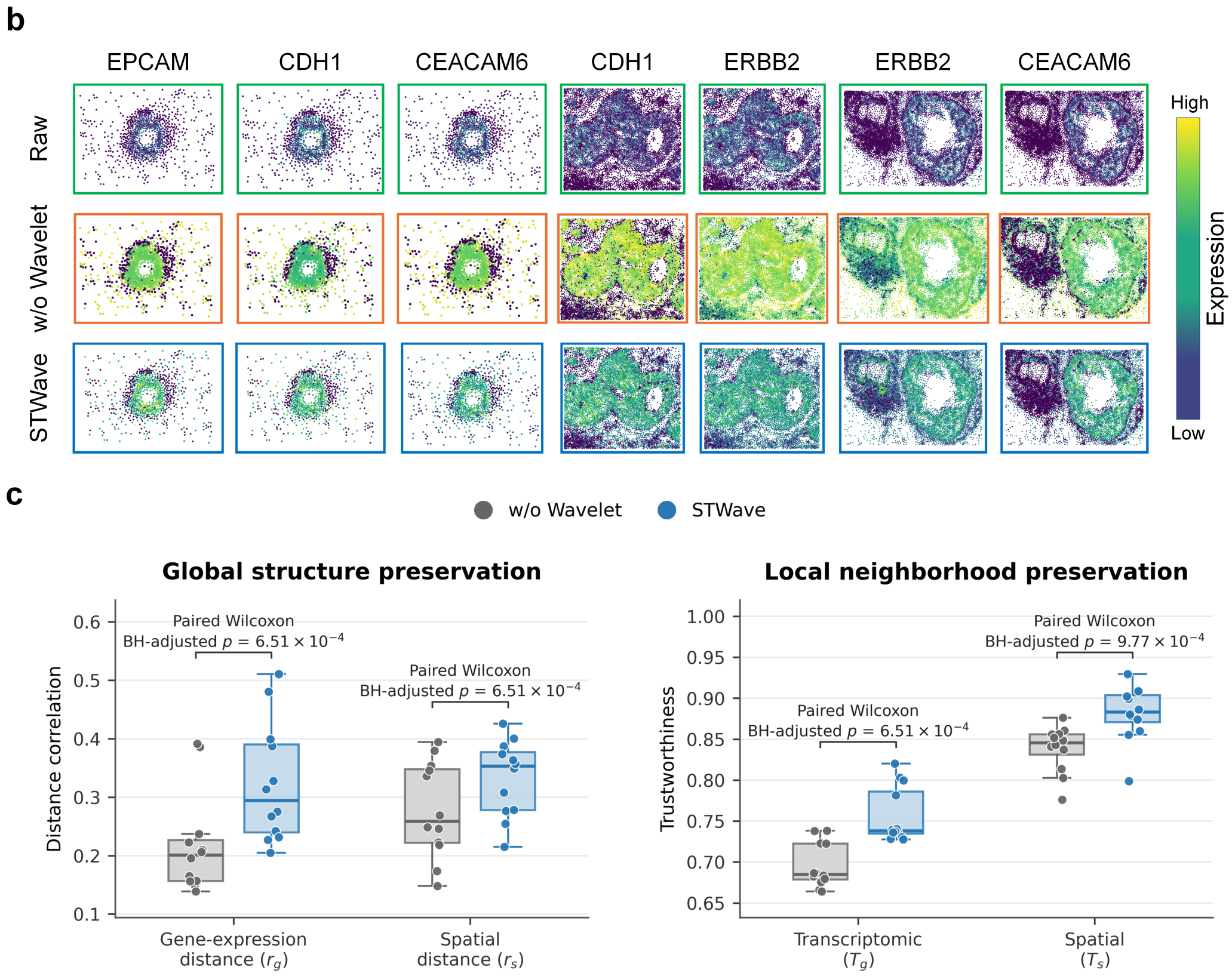

Figure S. 30 Clustering-agnostic evaluation of the wavelet-transform module on the Xenium human breast cancer and human DLPFC datasets. a. Comparison of region-specific relative marker signal gain (RSG) and reconstruction fidelity between STWave and STWave without the wavelet-transform module for seven region-of-interest (ROI)–marker pairs from three Xenium human breast cancer regions. RSG was calculated using all cells within each ROI, whereas the reconstruction-fidelity metrics were calculated among cells with detected raw expression of the corresponding marker. **b.** Spatial expression maps of the corresponding markers in the raw data and the expression reconstructed by STWave and STWave without the wavelet-transform module. **c.** Comparison of gene-expression distance preservation (r_g), spatial-distance preservation (r_s), transcriptomic Trustworthiness (T_g), and spatial Trustworthiness (T_s) in the embeddings generated by STWave and STWave without the wavelet-transform module across all 12 human DLPFC slices. Each point represents one matched DLPFC slice ($n = 12$); adjusted p values were obtained using two-sided Wilcoxon signed-rank tests followed by Benjamini–Hochberg correction across the four metrics.

Table S. 1 ST datasets used in our study. N/A: not available

Tissue	Platform	Resolution	Section ID	# of spots	# of genes	# of annotations
Human DLPFC	10x Visium	55 µm-diameter	151507	4266	33538	7
			151508	4384		7
			151509	4789		7
			151510	4634		7
			151669	3611		5
			151670	3498		5
			151671	4110		5
			151672	4015		5
			151673	3639		7
			151674	3673		7
			151675	3592		7
			151676	3460		7
Human colorectal cancer	Visium HD	8 µm × 8 µm	P2	545913	18085	N/A
Human breast cancer	Xenium	5 µm-diameter	Sample 1 replicate 1	167780	313	20
Human lung cancer	NanoString CosMx	N/A	Lung-9-1	87589	960	18
Mouse embryo	Stereo-seq	Bin50	E13.5_E1S2	64584	28208	19
			E14.5_E1S3	92928	18582	16
			E15.5_E1S2	109811	28154	22
			E16.5_E1S3	155741	28579	23
Simulate	N/A	N/A	N/A	5000	31007	10
				10000		
				20000		
				40000		
				80000		
				160000		
				240000		
				320000		
				480000		
				640000		
Human kidney cancer	Xenium	5 µm-diameter	Kidney Cancer (PRCC)	56510	377	N/A

Table S. 2 GPU memory usage of STWave and comparator methods across simulated datasets at multiple scales (GB).

Spots	STWave	SpaceFlow	PAST	SpaSEG	STAGATE	GraphST	SEDR	conST
5000	0.80	1.39	1.98	0.25	1.01	**0.79**	1.04	2.43
10000	1.41	1.52	3.12	**0.43**	1.81	1.80	2.24	6.01
20000	0.77	1.79	3.12	**0.72**	3.48	4.96	5.06	17.58
40000	1.63	2.15	3.12	**1.35**	6.95	15.90	13.08	62.19
80000	**1.60**	3.42	3.12	2.03	13.80	55.69	40.15	99.62
160000	**2.00**	5.63	3.12	3.94	27.45	N/A	N/A	N/A
240000	**2.08**	8.08	3.12	7.46	41.12	N/A	N/A	N/A
320000	**2.06**	10.44	3.12	7.76	54.82	N/A	N/A	N/A
480000	**2.14**	14.95	3.12	14.79	82.11	N/A	N/A	N/A
640000	**2.47**	19.17	3.12	19.67	N/A	N/A	N/A	N/A

Note: N/A indicates that the method was not executed on the device due to constraints on CPU, GPU, or other system resources.

Table S. 3 CPU memory usage of STWave and comparator methods across simulated datasets at multiple scales (GB).

Spots	STWave	SpaceFlow	PAST	SpaSEG	STAGATE	GraphST	SEDR	conST
5000	1.62	**1.26**	1.69	1.53	1.38	1.87	1.36	1.65
10000	1.85	**1.32**	2.33	1.84	1.58	4.17	2.67	2.40
20000	2.28	**1.59**	2.52	2.76	2.42	12.94	8.03	5.04
40000	2.92	**1.95**	2.76	3.79	3.64	45.78	27.87	14.69
80000	4.64	**3.82**	4.59	6.98	6.80	104.68	106.12	N/A
160000	8.28	**7.20**	10.01	15.04	13.84	255	255	N/A
240000	10.91	**9.89**	12.40	18.17	18.48	N/A	N/A	N/A
320000	15.89	**13.51**	18.96	27.77	26.41	N/A	N/A	N/A
480000	20.76	**18.37**	22.14	32.56	42.86	N/A	N/A	N/A
640000	30.34	**27.46**	39.88	75.76	N/A	N/A	N/A	N/A

Table S. 4 Runtime of STWave and comparator methods across simulated datasets at multiple scales (minutes).

Spots	STWave	SpaceFlow	PAST	SpaSEG	STAGATE	GraphST	SEDR	conST
5000	**0.138**	0.460	4.03	0.176	0.204	0.754	0.340	1.25
10000	0.260	0.600	3.95	**0.264**	0.379	1.80	0.451	3.67
20000	0.537	1.65	4.75	**0.353**	0.576	4.25	1.22	8.64
40000	1.05	2.85	5.03	**0.647**	0.807	13.6	14.3	32.4
80000	3.26	4.43	7.17	**1.93**	1.57	42.1	39.0	N/A
160000	4.36	13.4	9.16	**2.45**	3.14	N/A	N/A	N/A
240000	6.47	33.3	17.8	**3.68**	4.65	N/A	N/A	N/A
320000	8.80	25.7	35.6	**5.95**	7.06	N/A	N/A	N/A
480000	13.3	55.9	68.1	**7.71**	10.2	N/A	N/A	N/A
640000	18.2	108	102	**11.2**	N/A	N/A	N/A	N/A

Table S. 5 Spatial clustering performance was evaluated by comparing STWave with other methods using the Adjusted Rand Index on ten simulated datasets.

Spots	STWave	SpaceFlow	PAST	SpaSEG	STAGATE	GraphST	SEDR	conST
5000	**0.547**	0.230	0.130	0.028	0.150	0.423	0.210	0.155
10000	**0.514**	0.263	0.110	0.000	0.340	0.317	0.158	0.463
20000	**0.665**	0.259	0.164	0.199	0.520	0.334	0.216	0.462
40000	**0.751**	0.395	0.150	0.139	0.410	0.362	0.118	0.321
80000	**0.618**	0.401	0.372	0.167	0.406	0.341	0.272	N/A
160000	**0.638**	0.412	0.453	0.389	0.636	N/A	N/A	N/A
240000	**0.713**	0.517	0.620	0.547	0.575	N/A	N/A	N/A
320000	**0.571**	0.490	0.377	0.353	0.562	N/A	N/A	N/A
480000	**0.670**	0.350	0.258	0.312	0.538	N/A	N/A	N/A
640000	**0.565**	0.298	0.209	0.322	N/A	N/A	N/A	N/A
Mean	**0.625 ± 0.076**	0.361 ± 0.099	0.284 ± 0.167	0.246 ± 0.170	0.460 ± 0.150	0.356 ± 0.041	0.195 ± 0.059	0.350 ± 0.146

Table S. 6 Spatial clustering performance was evaluated by comparing STWave with other methods using the Normalized Mutual Information on ten simulated datasets.

Spots	STWave	SpaceFlow	PAST	SpaSEG	STAGATE	GraphST	SEDR	conST
5000	**0.640**	0.328	0.191	0.0972	0.289	0.478	0.385	0.142
10000	**0.630**	0.398	0.278	0.158	0.450	0.471	0.373	0.395
20000	**0.665**	0.414	0.332	0.347	0.548	0.553	0.463	0.567
40000	**0.741**	0.536	0.309	0.395	0.546	0.565	0.328	0.493
80000	**0.728**	0.551	0.531	0.447	0.571	0.561	0.530	N/A
160000	**0.794**	0.600	0.590	0.578	0.754	N/A	N/A	N/A
240000	**0.730**	0.635	0.626	0.630	0.718	N/A	N/A	N/A
320000	**0.759**	0.632	0.589	0.547	0.728	N/A	N/A	N/A
480000	**0.710**	0.559	0.481	0.563	0.642	N/A	N/A	N/A
640000	**0.612**	0.533	0.478	0.537	N/A	N/A	N/A	N/A
Mean	**0.701 ± 0.061**	0.519 ± 0.104	0.440 ± 0.152	0.430 ± 0.182	0.583 ± 0.150	0.525 ± 0.047	0.416 ± 0.080	0.399 ± 0.186

Table S. 7 GPU usage, CPU usage, Training time, and Adjusted Rand Index performance of STWave were evaluated on three simulated datasets across multiple patch configurations with varying spot counts.

Metric	Data scales	1250	2500	5000	10000	20000	40000	Mean	Std	Median
GPU	160000	0.4	0.72	1.14	2.15	3.58	7.41	2.57	2.64	1.65
	240000	0.49	0.68	1.1	2.15	3.13	4.83	2.06	1.68	1.63
	320000	0.53	0.79	1.01	2.1	2.68	7.56	2.45	2.64	1.56
CPU	160000	10.48	9.67	9.95	9.83	9.85	9.81	9.93	0.283	9.84
	240000	24.76	16.17	13.01	10.95	9.70	8.60	13.87	5.97	11.98
	320000	20.24	18.74	19.04	19.08	19.26	19.35	19.29	0.513	19.17
Train time	160000	22.74	11.86	9.56	7.12	5.99	5.47	10.46	6.475	8.34
	240000	31.78	10.14	9.19	9.33	9.35	8.78	13.10	9.16	9.34
	320000	41.19	23.51	18.74	15.34	14.56	11.39	20.79	10.81	17.04
ARI	160000	0.628	0.589	0.616	**0.638**	0.428	0.461	0.560	0.091	0.602
	240000	0.563	0.571	0.528	**0.713**	0.575	0.478	0.571	0.079	0.567
	320000	0.543	0.563	0.574	**0.571**	0.527	0.531	0.551	0.020	0.553

Note: GPU usage and CPU usage are reported in GB, and training time is reported in minutes. The experiments were conducted on an NVIDIA GeForce RTX 3090 with 24 GB GPU memory and a CPU with 60 GB system memory.

Table S. 8 Spatial clustering performance was evaluated by comparing STWave with other methods using the Adjusted Rand Index on twelve human DLPFC slices.

Method	151507	151508	151509	151510	151669	151670	151671	151672	151673	151674	151675	151676	Mean
STWave	**0.576**	**0.525**	**0.586**	**0.545**	**0.662**	**0.649**	**0.817**	**0.796**	**0.631**	**0.630**	**0.673**	**0.613**	**0.642 ± 0.089**
BayesSpace	0.433	0.386	0.352	0.436	0.456	0.412	0.505	0.740	0.454	0.279	0.442	0.321	0.435 ± 0.115
CCST	0.544	0.328	0.430	0.440	0.356	0.521	0.648	0.630	0.392	0.451	0.379	0.524	0.470 ± 0.104
conST	0.267	0.153	0.446	0.270	0.382	0.397	0.424	0.522	0.414	0.439	0.318	0.443	0.373 ± 0.102
DeepGFT	0.507	0.444	0.544	0.483	0.269	0.382	0.604	0.577	0.617	0.629	0.572	0.599	0.519 ± 0.109
MuCoST	0.446	0.474	0.483	0.477	0.467	0.439	0.577	0.600	0.583	0.600	0.531	0.525	0.517 ± 0.061
GraphST	0.445	0.444	0.454	0.493	0.423	0.411	0.581	0.607	0.616	0.455	0.512	0.544	0.499 ± 0.072
PAST	0.233	0.278	0.393	0.372	0.625	0.459	0.557	0.475	0.421	0.482	0.516	0.509	0.443 ± 0.112
SEDR	0.492	0.490	0.486	0.486	0.312	0.328	0.594	0.597	0.600	0.614	0.582	0.479	0.505 ± 0.102
Seurat	0.349	0.394	0.299	0.316	0.298	0.336	0.324	0.221	0.368	0.317	0.284	0.293	0.317 ± 0.044
SpaceFlow	0.367	0.132	0.087	0.261	0.235	0.247	0.386	0.221	0.267	0.228	0.315	0.288	0.253 ± 0.085
SpaGCN	0.370	0.308	0.290	0.427	0.190	0.148	0.339	0.354	0.416	0.334	0.320	0.304	0.317 ± 0.081
SpatialPCA	0.507	0.481	0.516	0.428	0.366	0.499	0.554	0.561	0.536	0.507	0.492	0.586	0.503 ± 0.060
spaVAE	0.303	0.315	0.336	0.373	0.373	0.355	0.231	0.534	0.344	0.529	0.402	0.473	0.381 ± 0.091
STAGATE	0.498	0.473	0.502	0.457	0.396	0.236	0.604	0.574	0.556	0.412	0.595	0.598	0.492 ± 0.108
Scanpy	0.350	0.282	0.213	0.301	0.209	0.196	0.376	0.410	0.377	0.354	0.299	0.285	0.304 ± 0.071
SpaSEG	0.319	0.502	0.466	0.392	0.446	0.266	0.435	0.331	0.476	0.392	0.465	0.504	0.416 ± 0.077
stLearn	0.334	0.283	0.309	0.261	0.262	0.163	0.374	0.334	0.234	0.271	0.295	0.275	0.283 ± 0.054

Table S. 9 Spatial clustering performance was evaluated by comparing STWave with other methods using the Normalized Mutual Information on twelve human DLPFC slices.

Method	151507	151508	151509	151510	151669	151670	151671	151672	151673	151674	151675	151676	Mean
STWave	**0.667**	**0.629**	0.648	0.575	**0.624**	**0.576**	**0.683**	0.678	0.687	0.704	**0.706**	0.672	**0.654 ± 0.045**
BayesSpace	0.627	0.530	0.566	0.570	0.558	0.485	0.604	**0.702**	0.639	0.468	0.638	0.492	0.573 ± 0.072
CCST	0.646	0.519	0.623	**0.608**	0.529	0.485	0.591	0.584	0.582	0.558	0.563	0.608	0.575 ± 0.046
conST	0.374	0.225	0.605	0.418	0.535	0.517	0.510	0.643	0.601	0.631	0.473	0.621	0.513 ± 0.125
DeepGFT	0.657	0.626	**0.652**	0.599	0.443	0.503	0.656	0.665	**0.703**	**0.732**	0.672	0.682	0.632 ± 0.083
MuCoST	0.644	0.632	0.639	0.597	0.601	0.565	0.673	0.683	0.702	0.710	0.669	0.606	0.643 ± 0.045
GraphST	0.622	0.585	0.609	0.597	0.501	0.525	0.658	0.680	0.697	0.593	0.603	0.629	0.608 ± 0.057
PAST	0.461	0.525	0.532	0.527	0.588	0.503	0.630	0.566	0.590	0.617	0.582	0.629	0.562 ± 0.053
SEDR	0.659	0.628	0.634	0.590	0.477	0.439	0.643	0.641	0.687	0.725	0.699	0.619	0.620 ± 0.085
Seurat	0.433	0.404	0.348	0.403	0.293	0.341	0.343	0.281	0.415	0.368	0.337	0.379	0.362 ± 0.047
SpaceFlow	0.499	0.261	0.174	0.312	0.319	0.259	0.437	0.223	0.365	0.267	0.371	0.417	0.325 ± 0.095
SpaGCN	0.493	0.434	0.427	0.498	0.339	0.293	0.436	0.442	0.553	0.413	0.433	0.470	0.436 ± 0.069
SpatialPCA	0.632	0.600	0.639	0.599	0.543	0.537	0.622	0.655	0.695	0.611	0.619	**0.696**	0.621 ± 0.049
spaVAE	0.482	0.468	0.469	0.440	0.537	0.503	0.362	0.659	0.510	0.680	0.578	0.628	0.526 ± 0.094
STAGATE	0.648	0.621	0.632	0.591	0.540	0.409	0.680	0.660	0.689	0.535	0.698	0.694	0.616 ± 0.086
Scanpy	0.438	0.368	0.330	0.368	0.256	0.277	0.387	0.421	0.486	0.451	0.444	0.434	0.388 ± 0.072
SpaSEG	0.504	0.614	0.592	0.554	0.570	0.431	0.544	0.509	0.617	0.556	0.567	0.621	0.557 ± 0.055
stLearn	0.436	0.350	0.478	0.434	0.319	0.269	0.447	0.454	0.403	0.420	0.470	0.444	0.410 ± 0.065

Table S. 10 Adjusted Rand Index performance of STWave was evaluated on twelve human DLPFC slices across different values of the loss weight α.

Section	5	10	15	20	25	30
151507	0.500	0.554	0.567	**0.576**	0.565	0.451
151508	0.504	0.413	0.444	**0.525**	0.439	0.407
151509	0.350	0.473	0.351	**0.586**	0.500	0.354
151510	0.444	0.447	0.450	**0.545**	0.471	0.480
151669	0.464	0.472	0.449	**0.662**	0.449	0.466
151670	0.470	0.457	0.456	**0.649**	0.382	0.458
151671	0.578	0.590	0.591	0.817	**0.821**	0.586
151672	0.577	0.575	0.578	**0.796**	0.574	0.573
151673	0.566	0.580	0.599	**0.631**	0.553	0.601
151674	0.542	0.545	0.404	**0.630**	0.477	0.620
151675	0.504	0.550	0.634	**0.673**	0.544	0.640
151676	0.542	0.588	0.530	**0.613**	0.531	0.461
Mean	0.503 ± 0.066	0.520 ± 0.063	0.504 ± 0.090	**0.642 ± 0.089**	0.525 ± 0.110	0.508 ± 0.092

Table S. 11 Normalized Mutual Information performance of STWave was evaluated on twelve human DLPFC slices across different values of the loss weight α.

Section	5	10	15	20	25	30
151507	0.644	0.658	0.650	**0.667**	0.647	0.555
151508	0.616	0.527	0.586	**0.629**	0.585	0.518
151509	0.525	0.613	0.537	**0.648**	0.568	0.519
151510	**0.578**	0.568	0.572	0.575	0.562	0.567
151669	0.535	0.538	0.531	**0.624**	0.533	0.534
151670	0.492	0.485	0.495	**0.576**	0.502	0.526
151671	0.596	0.606	0.603	0.683	**0.690**	0.600
151672	0.640	0.637	0.637	**0.678**	0.641	0.641
151673	0.680	0.675	0.686	**0.687**	0.657	0.686
151674	0.647	0.652	0.509	**0.704**	0.600	0.679
151675	0.596	0.634	0.699	**0.706**	0.630	0.688
151676	0.647	0.654	0.637	**0.672**	0.641	0.565
Mean	0.600 ± 0.058	0.604 ± 0.061	0.595 ± 0.068	**0.654 ± 0.045**	0.605 ± 0.056	0.590 ± 0.067

Table S. 12 Mean and standard deviation of ARI and NMI for STWave on the Xenium human breast cancer dataset across different α values from three independent runs with different random seeds.

Metric	5	10	15	20	25	30
ARI	0.415 ± 0.025	0.401 ± 0.013	0.402 ± 0.027	**0.441 ± 0.026**	0.396 ± 0.058	0.367 ± 0.030
NMI	**0.554 ± 0.013**	0.548 ± 0.007	0.544 ± 0.004	0.549 ± 0.001	0.544 ± 0.007	0.540 ± 0.006

Table S. 13 Mean and standard deviation of ARI and NMI for STWave on the mouse embryo dataset across different α values from three independent runs with different random seeds.

Metric	5	10	15	20	25	30
ARI	0.381 ± 0.017	0.377 ± 0.007	0.395 ± 0.003	**0.415 ± 0.018**	0.394 ± 0.015	0.392 ± 0.013
NMI	0.604 ± 0.006	0.604 ± 0.003	0.606 ± 0.004	**0.612 ± 0.001**	0.601 ± 0.004	0.605 ± 0.010

Table S. 14 Clustering performance on boundary and interior regions under different boundary ranges using the Xenium human breast cancer dataset.

Boundary range	Boundary spots (%)	ARI Boundary	ARI Interior	NMI Boundary	NMI Interior
0.5	0.1771	0.4693 ± 0.03875	0.4476 ± 0.002878	0.6267 ± 0.02441	0.5646 ± 0.009425
1.0	0.3372	0.4302 ± 0.01085	0.4478 ± 0.002877	0.5951 ± 0.0119	0.5646 ± 0.009431
1.5	0.5206	0.4608 ± 0.008447	0.4476 ± 0.002890	0.5863 ± 0.009022	0.5647 ± 0.009426
2.0	0.6895	0.4430 ± 0.008491	0.4477 ± 0.002913	0.5778 ± 0.009250	0.5647 ± 0.009443
2.5	0.8742	0.4442 ± 0.006867	0.4477 ± 0.002939	0.5752 ± 0.009183	0.5647 ± 0.009441

Table S. 15 Quantification of region-specific relative marker signal gain on the Xenium human breast cancer dataset. Raw and amplified expression maps were independently normalized to $[0, 1]$ using robust min–max scaling based on the 1st and 99th percentiles. The relative signal gain was calculated as the ratio of the mean normalized marker signal in the amplified map to that in the raw map within the corresponding H&E-defined region.

Region	Gene	n spots	Raw signal	Amplified signal	Relative gain	Δ signal
0	EPCAM	852	0.094	0.416	4.42×	0.322
0	CDH1	852	0.233	0.485	2.08×	0.252
0	CEACAM6	852	0.167	0.489	2.92×	0.321
1	CDH1	7149	0.186	0.451	2.43×	0.265
1	ERBB2	7149	0.220	0.524	2.39×	0.305
2	ERBB2	8426	0.172	0.423	2.46×	0.251
2	CEACAM6	8426	0.156	0.349	2.23×	0.192

Table S. 16 Number of clusters produced by each method on the CosMx SMI human lung cancer dataset under different Leiden resolution parameters.

Resolution	SpaSEG	PAST	STAGATE	PCA	SpaceFlow	SEDR	HERGAST	STWave
0.05	3	2	3	2	1	2	2	2
0.10	3	2	3	3	2	3	3	3
0.20	73	5	7	4	4	6	7	4
0.30	147	6	8	6	4	7	8	5
0.50	60	9	10	9	10	11	13	7
1.00	204	16	22	16	27	18	21	15
1.50	296	25	28	21	47	26	29	24
2.00	379	34	39	27	74	34	36	31

Table S. 17 Evaluation of clustering metrics for STWave and STWave without the wavelet transform on the DLPFC and Xenium human breast cancer datasets. The DLPFC results were summarized across 12 slices and three random trials, and the Xenium human breast cancer results were summarized across three random trials.

Dataset	Method	Metric	STWave	STWave w/o wavelet	Δ
**DLPFC**					
DLPFC	Mclust	ARI	0.593 ± 0.074	0.456 ± 0.080	$+0.137 \pm 0.095$
DLPFC	Mclust	NMI	0.658 ± 0.041	0.592 ± 0.055	$+0.066 \pm 0.060$
DLPFC	Mclust	AMI	0.657 ± 0.041	0.592 ± 0.056	$+0.066 \pm 0.061$
DLPFC	Mclust	HS	0.659 ± 0.042	0.607 ± 0.056	$+0.053 \pm 0.067$
DLPFC	Mclust	Completeness	0.658 ± 0.045	0.581 ± 0.063	$+0.077 \pm 0.065$
DLPFC	Mclust	V-measure	0.658 ± 0.041	0.592 ± 0.055	$+0.066 \pm 0.060$
DLPFC	Mclust	FMI	0.689 ± 0.076	0.581 ± 0.076	$+0.108 \pm 0.074$
DLPFC	Mclust	Purity	0.793 ± 0.053	0.738 ± 0.085	$+0.055 \pm 0.063$
DLPFC	K-means	ARI	0.337 ± 0.065	0.269 ± 0.068	$+0.067 \pm 0.068$
DLPFC	K-means	NMI	0.484 ± 0.051	0.382 ± 0.074	$+0.102 \pm 0.051$
DLPFC	K-means	AMI	0.483 ± 0.051	0.380 ± 0.074	$+0.102 \pm 0.051$
DLPFC	K-means	HS	0.496 ± 0.039	0.382 ± 0.065	$+0.114 \pm 0.053$
DLPFC	K-means	Completeness	0.474 ± 0.065	0.383 ± 0.085	$+0.091 \pm 0.055$
DLPFC	K-means	V-measure	0.484 ± 0.051	0.382 ± 0.074	$+0.102 \pm 0.051$
DLPFC	K-means	FMI	0.487 ± 0.057	0.445 ± 0.043	$+0.042 \pm 0.057$
DLPFC	K-means	Purity	0.666 ± 0.059	0.622 ± 0.056	$+0.045 \pm 0.048$
DLPFC	Louvain	ARI	0.391 ± 0.059	0.254 ± 0.073	$+0.137 \pm 0.085$
DLPFC	Louvain	NMI	0.510 ± 0.043	0.370 ± 0.075	$+0.140 \pm 0.058$
DLPFC	Louvain	AMI	0.509 ± 0.043	0.369 ± 0.075	$+0.140 \pm 0.058$
DLPFC	Louvain	HS	0.529 ± 0.040	0.386 ± 0.070	$+0.144 \pm 0.061$
DLPFC	Louvain	Completeness	0.493 ± 0.049	0.357 ± 0.081	$+0.136 \pm 0.057$
DLPFC	Louvain	V-measure	0.510 ± 0.043	0.370 ± 0.075	$+0.140 \pm 0.058$
DLPFC	Louvain	FMI	0.527 ± 0.058	0.417 ± 0.046	$+0.110 \pm 0.068$
DLPFC	Louvain	Purity	0.690 ± 0.070	0.615 ± 0.058	$+0.075 \pm 0.058$
DLPFC	Leiden	ARI	0.396 ± 0.054	0.244 ± 0.066	$+0.153 \pm 0.064$
DLPFC	Leiden	NMI	0.524 ± 0.038	0.364 ± 0.074	$+0.159 \pm 0.059$
DLPFC	Leiden	AMI	0.523 ± 0.038	0.363 ± 0.073	$+0.160 \pm 0.059$
DLPFC	Leiden	HS	0.542 ± 0.037	0.379 ± 0.066	$+0.163 \pm 0.063$
DLPFC	Leiden	Completeness	0.508 ± 0.046	0.352 ± 0.081	$+0.156 \pm 0.058$
DLPFC	Leiden	V-measure	0.524 ± 0.038	0.364 ± 0.074	$+0.159 \pm 0.059$
DLPFC	Leiden	FMI	0.532 ± 0.043	0.409 ± 0.034	$+0.124 \pm 0.050$
DLPFC	Leiden	Purity	0.701 ± 0.066	0.612 ± 0.058	$+0.088 \pm 0.049$
**Xenium human breast cancer**					
HBC	K-means	ARI	0.340 ± 0.057	0.180 ± 0.006	$+0.160 \pm 0.059$
HBC	K-means	NMI	0.501 ± 0.014	0.347 ± 0.007	$+0.154 \pm 0.021$
HBC	K-means	AMI	0.501 ± 0.014	0.347 ± 0.007	$+0.154 \pm 0.021$
HBC	K-means	HS	0.508 ± 0.026	0.385 ± 0.009	$+0.123 \pm 0.018$
HBC	K-means	Completeness	0.498 ± 0.048	0.316 ± 0.007	$+0.182 \pm 0.055$
HBC	K-means	V-measure	0.501 ± 0.014	0.347 ± 0.007	$+0.154 \pm 0.021$
HBC	K-means	FMI	0.421 ± 0.053	0.267 ± 0.006	$+0.153 \pm 0.056$
HBC	K-means	Purity	0.604 ± 0.034	0.518 ± 0.007	$+0.086 \pm 0.027$
HBC	Louvain	ARI	0.477 ± 0.041	0.257 ± 0.005	$+0.220 \pm 0.044$
HBC	Louvain	NMI	0.554 ± 0.006	0.359 ± 0.008	$+0.196 \pm 0.010$
HBC	Louvain	AMI	0.554 ± 0.006	0.359 ± 0.008	$+0.196 \pm 0.010$
HBC	Louvain	HS	0.521 ± 0.003	0.262 ± 0.002	$+0.259 \pm 0.002$
HBC	Louvain	Completeness	0.593 ± 0.018	0.572 ± 0.030	$+0.021 \pm 0.039$
HBC	Louvain	V-measure	0.554 ± 0.006	0.359 ± 0.008	$+0.196 \pm 0.010$
HBC	Louvain	FMI	0.549 ± 0.039	0.456 ± 0.014	$+0.092 \pm 0.049$
HBC	Louvain	Purity	0.603 ± 0.022	0.452 ± 0.005	$+0.151 \pm 0.022$
HBC	Leiden	ARI	0.436 ± 0.046	0.283 ± 0.026	$+0.152 \pm 0.034$
HBC	Leiden	NMI	0.557 ± 0.009	0.372 ± 0.007	$+0.185 \pm 0.003$
HBC	Leiden	AMI	0.557 ± 0.009	0.372 ± 0.007	$+0.185 \pm 0.003$
HBC	Leiden	HS	0.536 ± 0.010	0.279 ± 0.014	$+0.256 \pm 0.013$
HBC	Leiden	Completeness	0.581 ± 0.014	0.560 ± 0.030	$+0.021 \pm 0.044$
HBC	Leiden	V-measure	0.557 ± 0.009	0.372 ± 0.007	$+0.185 \pm 0.003$
HBC	Leiden	FMI	0.510 ± 0.043	0.468 ± 0.009	$+0.042 \pm 0.041$
HBC	Leiden	Purity	0.602 ± 0.011	0.461 ± 0.011	$+0.141 \pm 0.013$

References

[1] Satija, R., Farrell, J.A., Gennert, D., Schier, A.F., Regev, A.: Spatial reconstruction of single-cell gene expression data. Nature biotechnology **33**(5), 495–502 (2015)

[2] Wolf, F.A., Angerer, P., Theis, F.J.: Scanpy: large-scale single-cell gene expression data analysis. Genome biology **19**(1), 15 (2018)

[3] Pham, D., Tan, X., Xu, J., Grice, L.F., Lam, P.Y., Raghubar, A., Vukovic, J., Ruitenberg, M.J., Nguyen, Q.: stlearn: integrating spatial location, tissue morphology and gene expression to find cell types, cell-cell interactions and spatial trajectories within undissociated tissues. biorxiv, 2020–05 (2020)

[4] Hu, J., Li, X., Coleman, K., Schroeder, A., Ma, N., Irwin, D.J., Lee, E.B., Shinohara, R.T., Li, M.: Spagcn: Integrating gene expression, spatial location and histology to identify spatial domains and spatially variable genes by graph convolutional network. Nature methods **18**(11), 1342–1351 (2021)

[5] Zhao, E., Stone, M.R., Ren, X., Guenthoer, J., Smythe, K.S., Pulliam, T., Williams, S.R., Uytingco, C.R., Taylor, S.E., Nghiem, P., *et al.*: Spatial transcriptomics at subspot resolution with bayesspace. Nature biotechnology **39**(11), 1375–1384 (2021)

[6] Dong, K., Zhang, S.: Deciphering spatial domains from spatially resolved transcriptomics with an adaptive graph attention auto-encoder. Nature communications **13**(1), 1739 (2022)

[7] Li, J., Chen, S., Pan, X., Yuan, Y., Shen, H.-B.: Cell clustering for spatial transcriptomics data with graph neural networks. Nature Computational Science **2**(6), 399–408 (2022)

[8] Ren, H., Walker, B.L., Cang, Z., Nie, Q.: Identifying multicellular spatiotemporal organization of cells with spaceflow. Nature communications **13**(1), 4076 (2022)

[9] Shang, L., Zhou, X.: Spatially aware dimension reduction for spatial transcriptomics. Nature communications **13**(1), 7203 (2022)

[10] Zong, Y., Yu, T., Wang, X., Wang, Y., Hu, Z., Li, Y.: const: an interpretable multi-modal contrastive learning framework for spatial transcriptomics. BioRxiv, 2022–01 (2022)

[11] Li, Z., Chen, X., Zhang, X., Jiang, R., Chen, S.: Latent feature extraction with a prior-based self-attention framework for spatial transcriptomics. Genome Research **33**(10), 1757–1773 (2023)

[12] Long, Y., Ang, K.S., Li, M., Chong, K.L.K., Sethi, R., Zhong, C., Xu, H., Ong, Z., Sachaphibulkij, K., Chen, A., *et al.*: Spatially informed clustering, integration, and deconvolution of spatial transcriptomics with graphst. Nature Communications **14**(1), 1155 (2023)

[13] Tian, T., Zhang, J., Lin, X., Wei, Z., Hakonarson, H.: Dependency-aware deep generative models for multitasking analysis of spatial omics data. Nature Methods **21**(8), 1501–1513 (2024)

[14] Xu, H., Fu, H., Long, Y., Ang, K.S., Sethi, R., Chong, K., Li, M., Uddamvathanak, R., Lee, H.K., Ling, J., *et al.*: Unsupervised spatially embedded deep representation of spatial transcriptomics. Genome Medicine **16**(1), 12 (2024)

[15] Zhang, L., Liang, S., Wan, L.: A multi-view graph contrastive learning framework for deciphering spatially resolved transcriptomics data. Briefings in Bioinformatics **25**(4), 255 (2024)

[16] Bai, Y., Guo, X., Liu, K., Zheng, B., Wei, Y., Wang, Y., Zhang, W., Luo, Q., Yin, J., Wu, L., *et al.*: Spaseg: unsupervised deep learning for multi-task analysis of spatially resolved transcriptomics. Genome Biology **26**(1), 230 (2025)

[17] Sun, S., Liu, J., Li, G., Liu, B.: Deepgft: identifying spatial domains in spatial transcriptomics of complex and 3d tissue using deep learning and graph fourier transform. Genome Biology **26**(1), 153 (2025)

[18] Gong, Y., Yuan, X., Jiao, Q., Yu, Z.: Unveiling fine-scale spatial structures and amplifying gene expression signals in ultra-large st slices with hergast. Nature Communications **16**(1), 1–14 (2025)

[19] Rand, W.M.: Objective criteria for the evaluation of clustering methods. Journal of the American Statistical association **66**(336), 846–850 (1971)

[20] Hubert, L., Arabie, P.: Comparing partitions. Journal of classification **2**(1), 193–218 (1985)

[21] Strehl, A., Ghosh, J.: Cluster ensembles—a knowledge reuse framework for combining multiple partitions. Journal of machine learning research **3**(Dec), 583–617 (2002)

[22] Romano, S., Vinh, N.X., Bailey, J., Verspoor, K.: Adjusting for chance clustering comparison measures. Journal of Machine Learning Research **17**(134), 1–32 (2016)

[23] Rosenberg, A., Hirschberg, J.: V-measure: A conditional entropy-based external cluster evaluation measure. In: Proceedings of the 2007 Joint Conference on Empirical Methods in Natural Language Processing and Computational Natural Language Learning (EMNLP-CoNLL), pp. 410–420. Association for Computational Linguistics, Prague, Czech Republic (2007). https://aclanthology.org/D07-1043/

[24] Fowlkes, E.B., Mallows, C.L.: A method for comparing two hierarchical clusterings. Journal of the American statistical association **78**(383), 553–569 (1983)

[25] Manning, C.D., Raghavan, P., Schütze, H.: Introduction to Information Retrieval. Cambridge University Press, Cambridge (2008)

[26] Zhou, Y., Tang, C., Xiao, X., Zhan, X., Wang, T., Xiao, G., Xu, L.: Dimensionality reduction for visualizing spatially resolved profiling data using spasne. GigaScience **14**, 002 (2025)

[27] Venna, J., Kaski, S.: Neighborhood preservation in nonlinear projection methods: An experimental study. In: International Conference on Artificial Neural Networks, pp. 485–491 (2001). Springer

[28] Venna, J., Kaski, S.: Local multidimensional scaling. Neural Networks **19**(6-7), 889–899 (2006)

[29] Wang, L., Maletic-Savatic, M., Liu, Z.: Region-specific denoising identifies spatial co-expression patterns and intra-tissue heterogeneity in spatially resolved transcriptomics data. Nature Communications **13**(1), 6912 (2022)

[30] Yang, S.-T., Zhang, X.-F.: Engep: advancing spatial transcriptomics with accurate unmeasured gene expression prediction. Genome Biology **24**(1), 293 (2023)

[31] Song, T., Broadbent, C., Kuang, R.: Gntd: reconstructing spatial transcriptomes with graph-guided neural tensor decomposition informed by spatial and functional relations. Nature communications **14**(1), 8276 (2023)
